# Constitutive natural killer cell features associated with post-treatment control of SIV infection in the pVISCONTI study

**DOI:** 10.1038/s41467-026-76699-7

**Published:** 2026-08-22

**Authors:** Anaïs Chapel, Luis Romero-Martín, Caroline Passaes, Valérie Monceaux, Stevenn Volant, Delphine Desjardins, Adeline Melard, Annie David, Nathalie Dereuddre-Bosquet, Christine Rouzioux, Véronique Avettand-Fenoel, Michaela Müller-Trutwin, Roger Le Grand, Asier Sáez-Cirión

**Affiliations:** 1 Institut Pasteur, Université Paris Cité, Viral Reservoirs and Immune Control Unit, Paris, France; 2 Institut Pasteur, Université Paris Cité, HIV, Inflammation and Persistence Unit, Paris, France; 3 Institut Pasteur, Université Paris Cité, Bioinformatics and Biostatistics Hub, Paris, France; 4 Université Paris-Saclay, CEA, INSERM, UMR1184, Immunology of Viral Auto-immune, Hematological and Bacterial diseases (IMVA-HB/ IDMIT Department), Fontenay-aux-Roses/Le Kremlin-Bicêtre, France; 5 Université Paris Cité, INSERM, U1016; CNRS, UMR8104, Paris, France; 6 Université Paris Cité, Paris, France; 7 CHU d’Orléans, Orléans, France; 8 Université d’Orléans, Orléans, France

**Keywords:** Innate lymphoid cells, HIV infections, Viral host response

## Abstract

Post-treatment HIV controllers (PTC) offer a unique opportunity to uncover the determinants of durable remission after antiretroviral therapy (ART) interruption. Here, using six PTC and six post-treatment non-controllers from the pVISCONTI study in cynomolgus macaques, we identify associations between Natural Killer (NK) cell subsets and outcomes following ART interruption. NKG2Ahigh NK cells lacking NKp30/NKp46 (NKG2AhighNKp30-NKp46-), shaped by the MHC backgrounds of the animals, were linked to improved post-treatment control; this NK subset displayed tissue-homing potential and preserved functionality despite infection, characterized by cytokine production and degranulation. Conversely, NKG2AlowNKp30 + NKp46+ NK cells exhibited reduced cytotoxic potential and elevated IL-10/IL-17A production, and their pre-infection levels were associated with higher viremia and larger viral reservoirs after ART interruption. Our study suggests that constitutive NK cell features, preserved by early ART initiation, may provide a favorable immune environment for post-treatment control. Our findings identify the NKG2A axis as a potential target to improve outcomes after ART interruption

## Abstract (web summary)

Early ART initiation enables durable HIV remission but the underlying immune mechanisms remain unclear. Here, Chapel, Romero-Martin and colleagues present evidence that constitutive NK cell features are associated with outcomes following ART interruption in cynomolgus macaques.

## Introduction

Despite the effectiveness of antiretroviral therapy (ART) in suppressing HIV replication, the virus cannot be eradicated from the organism, and most individuals experience rapid viral rebound following analytical treatment interruption (ATI). However, a rare subset of individuals, known as post-treatment controllers (PTC), durably maintain low-level viremia after discontinuing ART. These individuals serve as important models for investigating the mechanisms underlying durable viral remission.

Several studies support that early and prolonged ART increases the likelihood of post-treatment control^1–4^. Further support comes from multiple meta-analyses of ATI studies, which show that the time to viral rebound correlates with the delay between HIV acquisition and ART initiation^5,6^. Although early treatment reduces the size of the viral reservoir^4,7^, preserves mucosal immune cell populations^8^ and immune response^9^, it remains unclear whether a specific window of time exists to develop effective immune responses that would support long-term control after ATI. Notably, only a small proportion -approximately 6%, according to some studies^6^-of individuals treated early achieve post-treatment control, though definitions vary among cohorts.

Distinct features identified in PTCs include low levels of HIV DNA in peripheral blood mononuclear cells (PBMCs)^2,10^, higher CD4+ nadir and CD4/CD8 ratio^6^, and lower T cell activation and exhaustion. Intact, replication-competent proviruses have been found in PTCs, pointing to a critical role of immune factors in these individuals^2,11,12^. However, post-treatment control is likely multifactorial, and the underlying mechanisms remain elusive.

We have recently identified an immunogenetic signature related to enhanced NK cell education via specific killer-cell immunoglobulin-like receptors (KIRs), which was highly prevalent among PTCs from the VISCONTI study and associated with higher probability of post-treatment control among people who started ART during primary infection^13^. This signature is typically accompanied by low HLA-E levels and elevated NK cell expression of NKG2A^14^. Other studies have reported the presence of NK cells with robust antiviral activities among PTCs^15,16^. These findings point to a critical role for NK cells in maintaining viral suppression after ART discontinuation, although the links remain unclear.

NK cells are highly heterogeneous and participate in diverse immune defense mechanisms. In the context of HIV infection, several NK cell-mediated mechanisms have been proposed to contribute to viral suppression, including (1) direct and antibody-dependent cytotoxicity^17,18^ (2) secretion of antiviral cytokines and chemokines that recruit other immune cells and inhibit viral replication^19^, and (3) modulation of the adaptive immune response through dendritic cell (DC) regulation^20^ or elimination of activated T cells^21,22^.

In the non-pathogenic African green monkey model of SIV infection, NK cells upregulate CXCR5, migrate to mesenteric lymph nodes, and help preserve germinal center integrity and SIV-specific IgA levels^23^. Additionally, NK cells with adaptive features and increased MHC-E-restricted activity have been implicated in SIV control in peripheral lymph nodes^24^. Together, these observations underscore the central role of NK cells in both direct viral clearance and in orchestrating effective adaptive immunity while mitigating chronic immune activation.

Previous analyses of NK cells in the context of post-treatment HIV control have been limited to a period of well-established control, preventing the exploration of their implication before and during ART interruption. We recently implemented a model of SIVmac_251_ infection of cynomolgus macaque (CyMs) to assess the impact of early (week 4 post-infection) versus late (week 24 post-infection) ART initiation on post-treatment control after 2 years of treatment (the p(rimate)VISCONTI study)^25^. The pVISCONTI study showed that early ART initiation strongly favored durable post-treatment SIV control in this model and provided a unique opportunity to explore longitudinally the underlying immune mechanisms.

Here, we study the role of NK cells in viral control after ART interruption by comparing the phenotype, function, and dynamics of NK cells in six PTC macaques and six post-treatment non-controller (PTNC) macaques from the pVISCONTI study. We show that constitutive NK cell features, shaped before SIV infection, correlate with sustained post-treatment control and reduced viral reservoir at the study endpoint. Moreover, we identified two opposing NK cell subsets that either negatively or positively influenced outcomes after ART interruption.

## Results

### The presence of specific NK cell subsets since baseline associated with outcome after treatment discontinuation

The objective of this study was to evaluate the role of NK cells in the achievement of SIV control following treatment discontinuation. We studied 12 animals from the pVISCONTI study. All macaques except one experienced a viral rebound after treatment interruption (7.18 × 10^6^ [6.19 × 10^6^, 11.46 × 10^6^] median and IQR peak SIV RNA copies/mL). However, 5 macaques (4 treated at W4 post-infection, p.i., and 1 treated at W24 p.i.) rapidly decreased those levels. In total, 6 animals maintained stable and controlled pVL after ATI (68 [24, 89] SIV RNA copies/mL at last follow-up), achieving the status of post-treatment controller (PTC). The other 6 macaques (1 treated at W4 p.i. and 5 treated at W24) maintained high pVL (2.41 × 10^4^ [1.01 × 10^4^, 4.37 × 10^4^] SIV RNA copies/mL at last follow-up) and were defined as post-treatment non-controllers (PTNC).

**Fig. 1:**
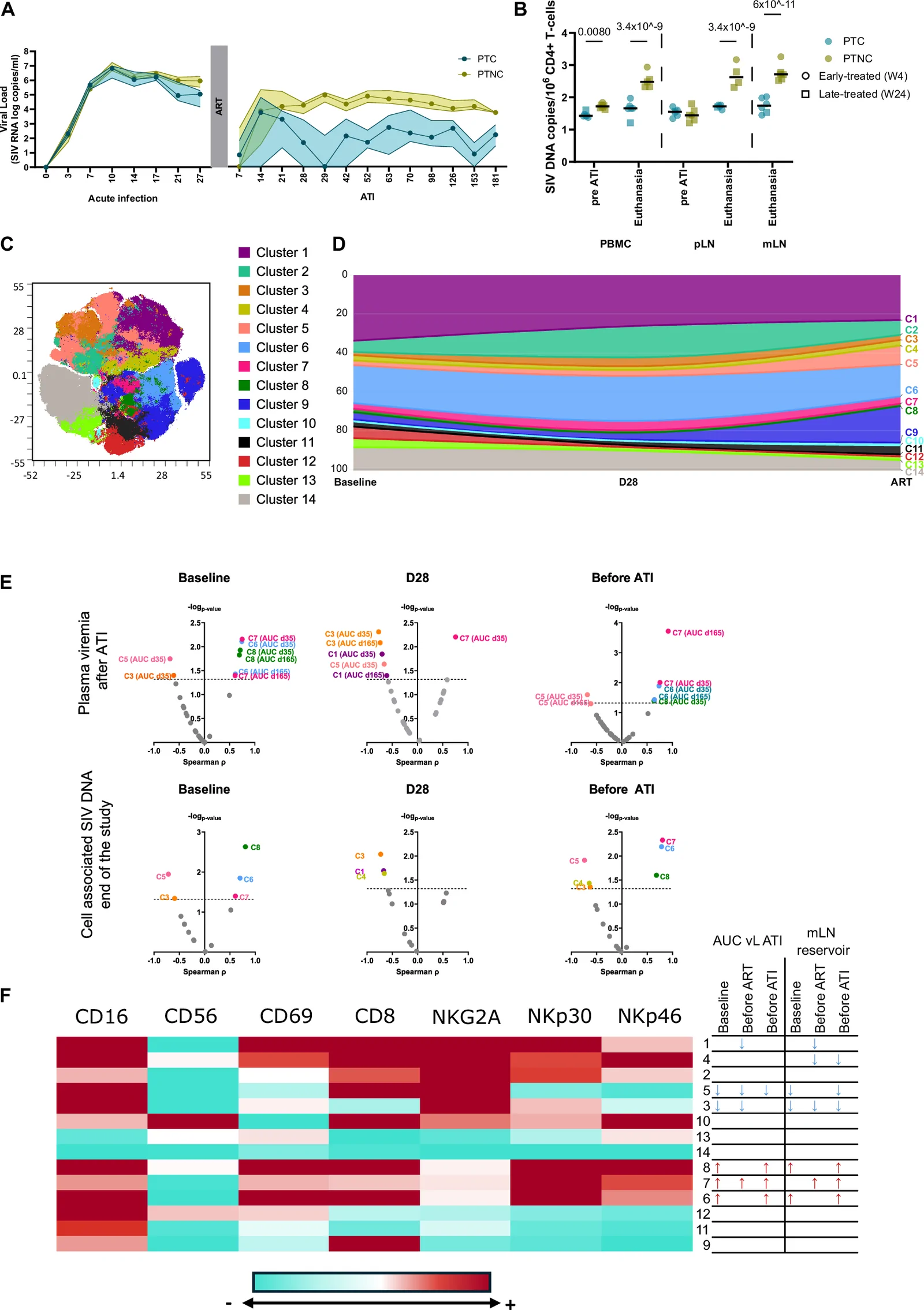
Unsupervised analysis of NK cell dynamics during the pVISCONTI study (*n* = 12 macaques). **A** Viral load (SIV RNA copies/mL) during the study (medians and IQR). **B** Viral reservoir (SIV DNA copies/10^6^ CD4^+^ T-cells). Each data point represents results with samples from one individual macaque (*n* = 12 individuals), horizontal lines represent the median. **C** Dimensional reduction and unsupervised on CD3^−^CD14^−^CD20^−^NKp80^+^ cells identified 14 different clusters of NK cells based on the expression of CD16, CD56, CD69, CD8, NKG2A, NKp30, and NKp46. **D** Relative abundance of the 14 clusters in PBMC at the Baseline, before ART, and before ATI. **E** Volcano plots showing *p*-value and Spearman ρ of the correlation between cluster abundance and AUC of viral load during ATI (top) or total SIV DNA copies/10^6^ CD4^+^ T-cells in mesenteric lymph nodes (mLN) at euthanasia (bottom). **F** Relative expression of the different markers that defined the 14 clusters. Dimensional reduction was performed by Fit-tSNE and clustering by FlowSOM. Multiple comparison was performed by Two-way ANOVA and False Discovery Rate (FDR) correction (Benjamini, Krieger and Yekutieli). Correlations were assessed using two-sided Spearman’s rank method. Significant values were considered when *p*-value < 0.05 and *q*-value < 0.05.

There were no differences in pVL between PTC and PTNC during the acute infection (Fig. 1A), and all animals had indetectable pVL at the time of ATI. In contrast, a difference was observed in CD4^+^ T-cells-associated total SIV-DNA levels pre-ATI (*p*-value = 0.0080). The frequency of cells carrying SIV-DNA strongly increased in PTNC after ATI, and higher levels than in PTCs were found in CD4^+^ T-cells from PBMC, peripheral and mesenteric lymph nodes at the end of the study (*p*-value < 0.0001) (Fig. 1B).

NK cell phenotype was longitudinally analyzed in fresh samples in blood at baseline, acute infection, during ART, and after ATI. NK cells were defined as CD3^-^CD14^-^CD19^-^NKp80^+^^26–28^ (Fig. S2), and dimensional reduction was set at 4000 NK cells/sample (total > 870.000 cells considered) by Fit-tSNE. Fourteen clusters were identified in an unsupervised manner by FlowSOM (Elbow Plot determination) based on the expression of CD16, CD56, CD69, CD8, NKG2A, NKp30 and NKp46 (Fig. 1C). Overall, the distribution of the NK cell clusters remained relatively stable before ATI, except for a decrease in cluster 12 (CD16^+^CD56^+^CD69^+^) at day 28 p.i. (*p* < 0.0001) and maintained after 2 years of ART (*p* = 0.0001) and an expansion of cluster 9 (CD16^+^CD56^−^) at the end of the ART period (*p* = 0.004), which recapitulates previous observations in the context of HIV/SIV infections^29^ (Fig. 1D).

To assess whether, individually, NK cell distribution at different timepoints might have influenced outcome after ATI, we performed a correlation analysis between the proportion of the 14 defined clusters and three virological parameters: the cumulative plasma viral load (as determined by the area under the curve, AUC) during viral rebound (first 35 days) or extended follow up (165 days) after ATI, and the cell-associated total SIV-DNA in the mLN at the end of the study. The frequency of four clusters (Clusters 1, 3, 4 and 5) at the baseline, at day 28 p.i. and/or before ATI showed negative correlations with cumulative viremia and/or viral reservoir, pointing to a beneficial impact of the presence of these cells on the level of viral control after ATI. Conversely, three clusters (Clusters 6, 7 and 8) showed positive correlations with the same virological parameters (Fig. 1E). Notably, all the clusters associated with better post-treatment control were characterized by a high expression of NKG2A and low to intermediate expression of NKp30 and/or NKp46. In contrast, clusters negatively linked to viral control showed low levels of NKG2A and medium to high levels of NKp30 and NKp46 (Fig. 1F).

In another longitudinal analysis, we used a panel that included DNAM-1, NKG2D, and CD161 (Fig. S3), activating and inhibitory receptors that have been highlighted in the context of HIV-1 control^30–32^, and CD215 (IL-15Rα), which is essential for NK cell development and function^33^. An unsupervised clustering did not reveal baseline or acute-infection associations between baseline clusters containing cells expressing CD161, CD215, DNAM-1, or NKG2D and the outcome after ATI (Fig. S3). We detected receptor-specific associations with post-ATI viremia in a limited number of clusters pre-ATI, including DNAM-1^+^ clusters that correlated positively and a CD215^+^ cluster (not expressing DNAM-1 or NKG2D) that correlated negatively with viremia. Notably, these associations emerged only at late time points on ART and reflected delay to treatment initiation (early vs late) rather than controller potential per se. Manual gating confirmed no significant differences in DNAM-1, NKG2D, CD161, or CD215 expression between PTC and PTNC during acute infection or prior to treatment interruption.

These findings suggest that the intrinsic characteristics of NK cell populations associated with NKG2A expression, already present at the baseline before infection, may influence the ability to control viral rebound after interruption of 2 years-long ART.

### Opposing effects of NKG2A^high^NKp30^−^NKp46^−^ and NKG2A^low^NKp30^+^NKp46^+^ cells overtime on the outcome after treatment discontinuation

**Fig. 2:**
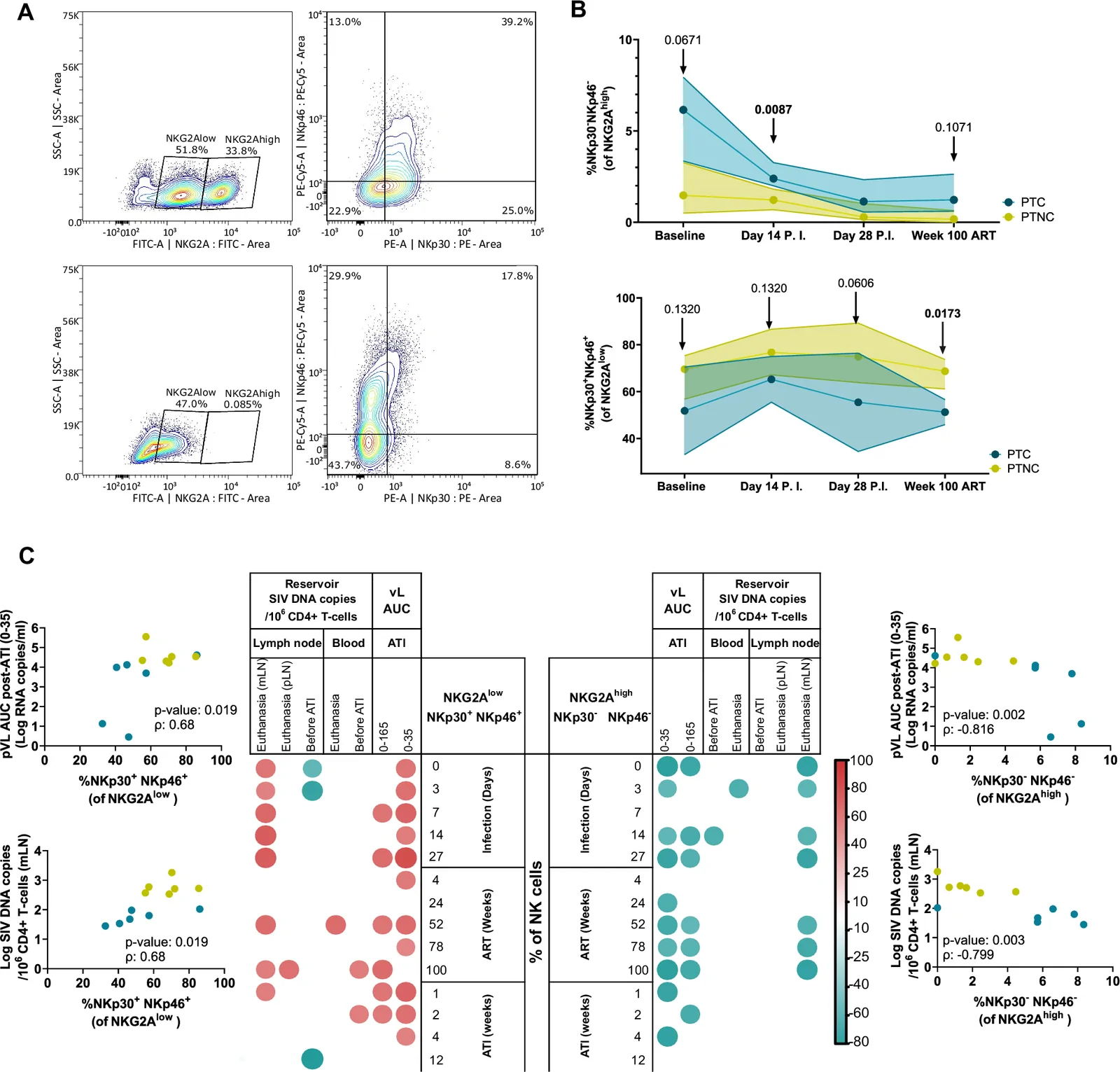
NKG2A^high^NKp30^−^NKp46^−^ and NKG2A^low^NKp30^+^NKp46^+^ dynamics (*n* = 12 macaques). **A** Gating strategy for NKG2A^high^NKp30^−^NKp46^−^ (top) and NKG2A^low^NKp30^+^NKp46^+^ (bottom) cells. **B** Frequency of NKG2A^high^NKp30^−^NKp46^−^ (top) or NKG2A^low^NKp30^+^NKp46^+^ (bottom) cells during acute infection and before ATI in PTC and PTNC. The darker line represents the median per group (areas indicate the IQR). **C** Correlations of baseline frequency of NKG2A^low^NKp30^+^NKp46^+^ cells and AUC of viral load during ATI and SIV reservoir in the mesenteric lymph node at Euthanasia (left). Correlogram showing the positive and negative correlations between NKG2A^high^NKp30^−^NKp46^−^ cells (left) and NKG2A^low^NKp30^+^NKp46^+^ cells (right) with the AUC of viral load during ATI and total SIV DNA copies/10^6^ CD4^+^ T-cells (center). Correlations of baseline frequency of NKG2A^high^NKp30^−^NKp46^−^ cells and AUC of viral load during ATI and SIV reservoir in the mesenteric lymph node at Euthanasia (right). Group comparisons were performed by two-tailed Mann-Whitney test. Correlations were assessed using two-sided Spearman’s rank method. Significant values were considered when *p*-value < 0.05 and *q*-value < 0.05. Only significant correlations are depicted in the correlogram.

The unsupervised analysis revealed diametrical opposed effects of cell subsets with a NKG2A^high^NKp30^−/low^NKp46^−/low^ vs NKG2A^low^NKp30^low/high^NKp46^low/high^ phenotypes. We therefore manually defined NKG2A^high^NKp30^-^NKp46^−^ and NKG2A^low^NKp30^+^NKp46^+^ NK cells to further explore their dynamics and potential implication on post-treatment control (Fig. 2A). These NK cell populations followed different dynamics following infection (Figs. 2B and S3). The proportion of NKG2A^high^NKp30^-^NKp46^-^ cells decreased following infection in all animals during the first weeks of primary infection, in line with previous studies^34^. ART initiation appeared to stop this drop but did not restore the frequency of these cells, even after 2 years of treatment. In contrast, the frequency of NKG2A^low^NKp30^+^NKp46^+^ cells increased during acute infection, but frequencies were similar to baseline after two years on ART (Fig. S4). Of note, PTC tended to have a higher proportion of NKG2A^high^NKp30^-^NKp46^−^ cells since the baseline than PTNC (Fig. 2B), a difference that was maintained until the time of ART interruption. At this time PTCs were in contrast, characterized by a lower frequency of NKG2A^low^NKp30^+^NKp46^+^ cells than PTNC. We observed the same trend when evaluating the proportion of NKG2A^high^NKp30^−^NKp46^−^ and NKG2A^low^NKp30^+^NKp46^+^ within the whole live lymphocyte compartment (Fig. S5). Accordingly, a strong negative correlation was found between baseline NKG2A^high^NKp30^−^NKp46^−^ cells and the cumulated plasma viral loads during viral rebound (days 0–35 post ATI) (*ρ* = −0.8161, *p*-value = 0.0019) and extended follow-up (days 0–165 post ATI) (*ρ* = −0.7426, *p*-value = 0.0075), or with the SIV reservoir in the mLN at Euthanasia (*ρ* = −0.7986, *p*-value = 0.0027) (Fig. 2C). Of note, negative correlations with the cumulated viremia after ATI (especially during viral rebound) and/or the cell-associated SIV-DNA levels in mLN at the end of the study were consistently found when we considered the frequency of NKG2A^high^NKp30^-^NKp46^-^ cells present during acute infection (days 0, 3, 14 and 27), after ART initiation (weeks 24, 52, 78 and 100) and following ATI (weeks 1, 4) (Fig. 2C). On the contrary, positive correlations were found between the frequency of NKG2A^low^NKp30^+^NKp46^+^ at baseline and at multiple time points during acute infection, after ART initiation and upon ATI with the cumulated viremia after ATI and the viral reservoir in mLN at the end of the study.

These data comforted the unsupervised analyses and identified two NK cell subsets which frequency at the time of infection and their trajectories throughout acute infection, ART, and ATI might differently influence the ATI outcome. A higher baseline frequency and sustained levels of NKG2A^high^NKp30^-^NKp46^-^ cells were associated with reduced viral rebound and better post-treatment control, while elevated levels of NKG2A^low^NKp30^+^NKp46^+^ cells were associated with higher viremia and SIV reservoir after ART interruption. Hereby, an intrinsic composition of NK cell subpopulations and the dynamics during the infection may decisively determine the capacity to contain the viral rebound after treatment discontinuation.

### Opposed tissue dynamics for NKG2A^high^NKp30^−^NKp46^−^ and NKG2A^low^NKp30^+^NKp46^+^ cells during acute infection and treatment

**Fig. 3:**
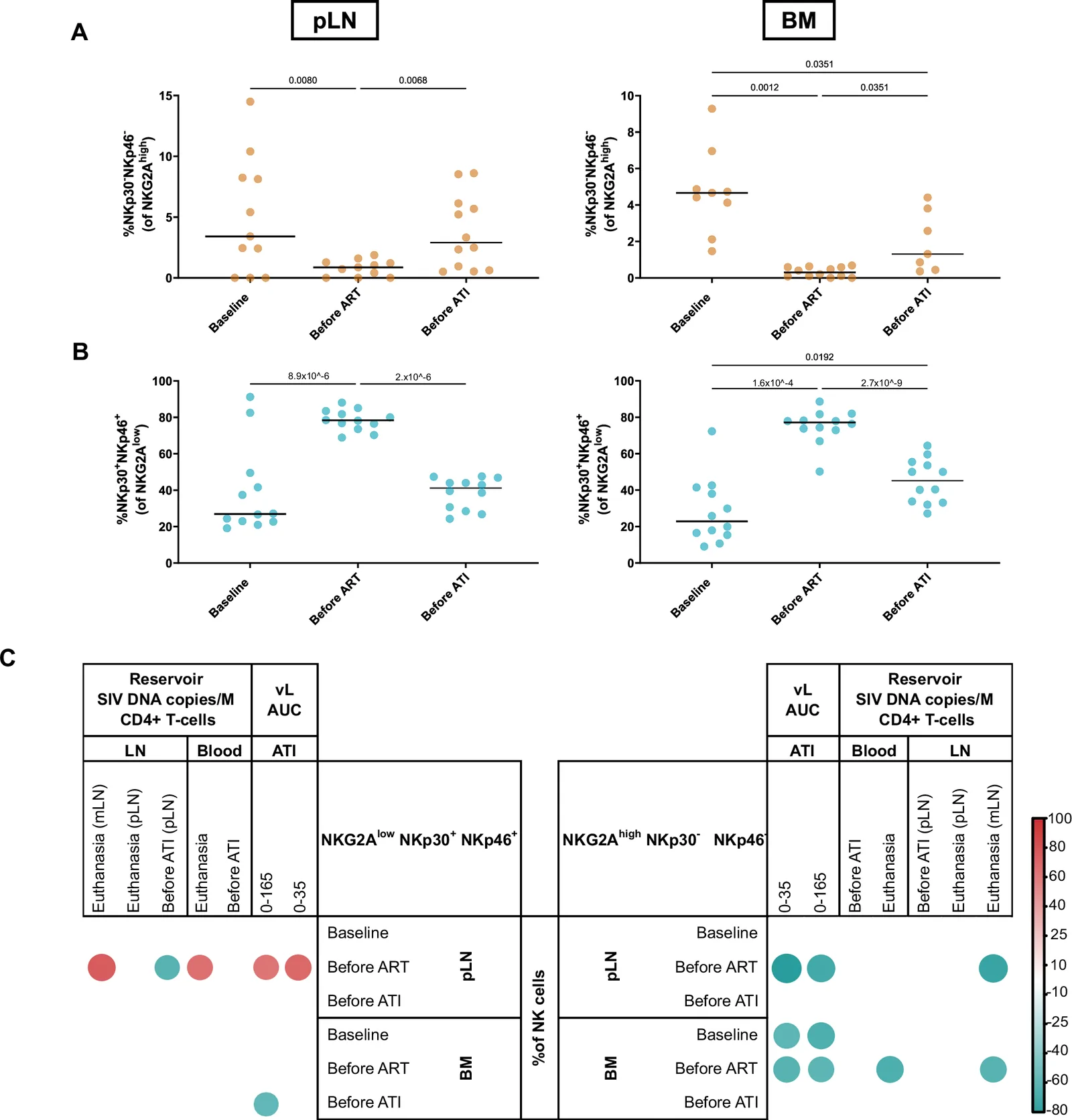
NKG2A^high^NKp30^−^NKp46^−^ and NKG2A^low^NKp30^+^NKp46^+^ dynamics in tissues (*n* = 12 macaques). **A** Frequency of NKG2A^high^NKp30^-^NKp46^-^ cells in the pLN and BM at the baseline, before ART and before ATI. **B** Frequency of NKG2A^low^NKp30^+^NKp46^+^ cells in the pLN and BM at the baseline, before ART and before ATI. **A**, **B** Each data point represents results with samples from one individual macaque (*n* = 12 individuals), horizontal lines represent the median. **C** Correlogram showing the positive and negative correlations between NKG2A^high^NKp30^−^NKp46^−^ cells (right) and NKG2A^low^NKp30^+^NKp46^+^ cells (left) with the AUC of viral load during ATI and total SIV DNA copies/10^6^ CD4^+^. Multiple comparisons were performed by Two-way ANOVA and False Discovery Rate (FDR) correction (Benjamini, Krieger, and Yekutieli). Correlations were assessed using two-sided Spearman’s rank method. Significant values were considered when *p*-value < 0.05 and *q*-value < 0.05. Only significant correlations are depicted in the correlogram.

The NK cell subsets of interest (NKG2A^high^NKp30^-^NKp46^-^ and NKG2A^low^NKp30^+^NKp46^+^) were not restrained to the blood, and we found them in multiple tissues, including the spleen, the liver, bronchoalveolar lavage (BAL) and the gut, at the end of the study (Fig. S6). We evaluated their distribution longitudinally in two different lymphoid tissues (pLN and bone marrow, BM). Consistent with the dynamics of circulating NKG2A^high^NKp30^-^NKp46^-^ cells, there was a sharp decrease in the frequency of this cell subset in both lymphoid tissues during the acute infection (Baseline vs Before ART, *p*-value = 0.008 and 0.0012 for pLN and BM, respectively). Levels recovered after ART, reaching basal levels in pLN but not in BM (*p* = 0.0351) (Fig. 3A). NKG2A^low^NKp30^+^NKp46^+^ cells followed opposite dynamics (Fig. 3B), increasing following infection in both pLN and BM (*p*-value < 0.0001). Their frequency decreased after 2 years of ART to almost-basal levels in pLN but remained elevated in BM (*p*-value = 0.0192) (Fig. 3B).

As in the case of circulating cells, the baseline frequency of NKG2A^high^NKp30^−^NKp46^−^ cells in BM correlated negatively with the cumulated viremia after ATI (days 0-35 and 0–165), and their frequency before ART in pLN and BM showed a negative correlation with both cumulated viremia after ATI and SIV reservoir levels in blood and mLN at the end of the study. In contrast, NKG2A^low^NKp30^+^NKp46^+^ cells in pLN showed positive correlations with cumulated viremia (days 0–35 and 0–165) and with SIV reservoir in PBMC and mLN at the end of the study.

Our results showed contrasting dynamics of NKG2A^high^NKp30^-^NKp46^-^ cells (which decreased during acute infection) and NKG2A^low^NKp30^+^NKp46^+^ cells (which accumulated during the same period), which were more marked in pLNs and BM than in blood. Of note, higher tissue levels of NKG2A^high^NKp30^−^NKp46^−^ and lower levels of NKG2A^low^NKp30^+^NKp46^+^ were associated with better viral control after ART interruption, mirroring the results found in blood.

### NKG2A^high^/NKG2A^low^ subsets are constitutive in each animal and are partly determined by their immunogenetic background

The NK cell subsets of interest were characterized by different expression levels of NKG2A, NKp30 and NKp46. The overall expression of NKp30 and NKp46 on NK cells was modulated by SIV infection and treatment. A reduction in the expression of NKp46 was observed at the end of the ART period, while NKp30 expression on NK cells increased during primary infection but returned to baseline levels after 2 years of ART. Overall, lower levels of NK cells expressing NKp30 were observed in PTCs at baseline (*p*-value = 0.029) and at the time of ART interruption (*p*-value = 0.0197) when compared to non-PTCs (Fig. S7).

**Fig. 4:**
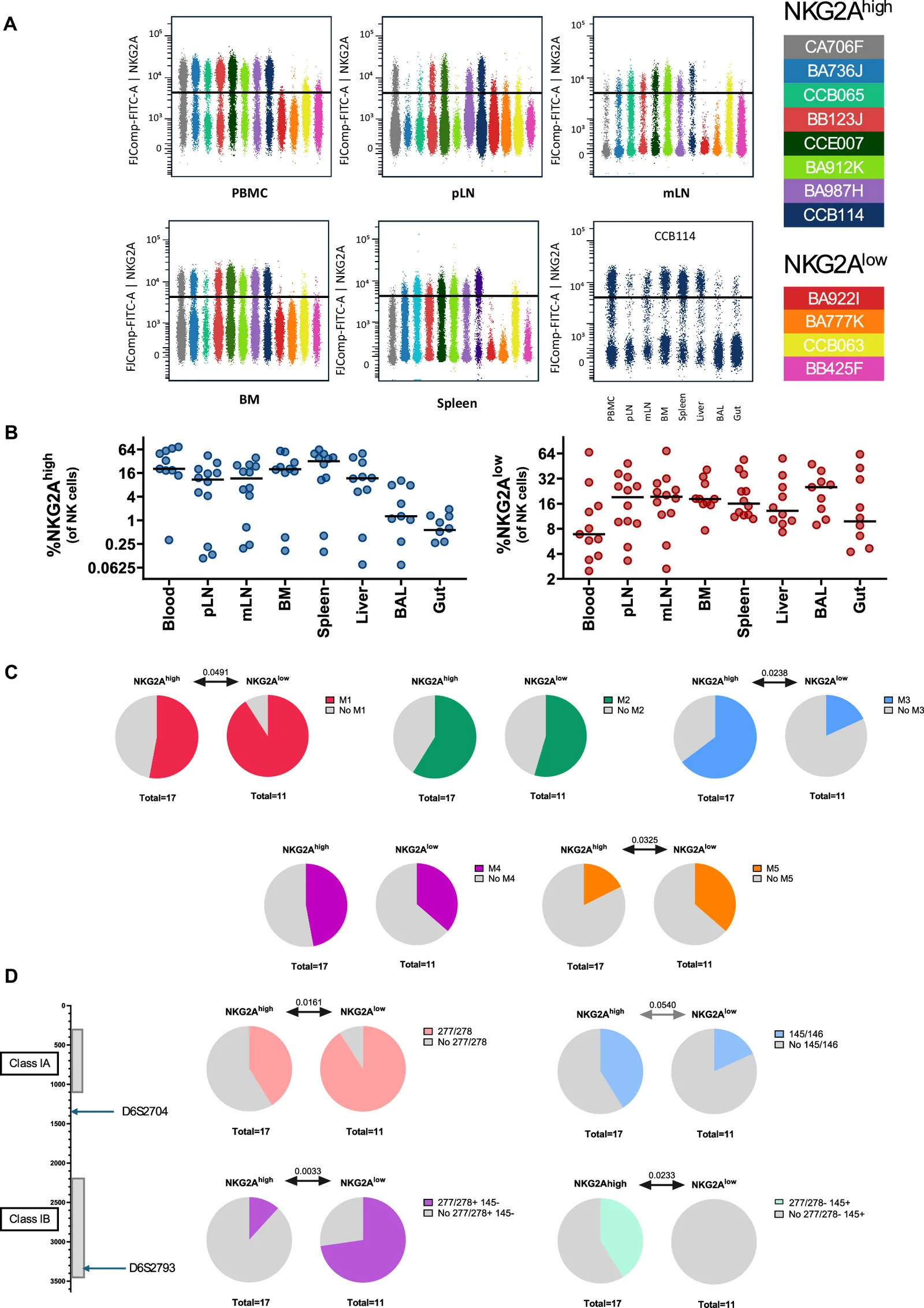
Physiological determinants of NKG2A^high^ expression. **A** The presence or absence of NKG2A^high^ NK cells is constitutive and observed across different lymphoid tissues. The last graph is representative of all tissues in one animal (CCB114) **B** Frequency of NKG2A^high^ and NKG2A^low^ cells in different tissues. Each data point represents results with samples from one individual macaque (*n* = 12 individuals), horizontal lines represent the median. **C** Absolute frequency of macaques harboring or not NKG2A^high^ cells based on M1, M2, M3, M4, or M5 MHC haplotypes (*n* = 28 macaques). **D** Localization and properties of the 277 and 145 microsatellite markers in the MHC region of cynomolgus macaques (based on information reported in Wiseman et al.). **E** Absolute frequency of macaques harboring or not NKG2A^high^ cells based on the 277 and/or 145 satellites (*n* = 28 macaques). Two-sided Fisher’s exact test was applied to evaluate absolute frequencies. Significant values were considered when *p*-value < 0.05.

In contrast to the other markers, the frequency of NKG2A^high^ NK cells remained stable during the complete follow-up. However, different constitutive levels were found among the macaques at the baseline: while all animals presented a NKG2A^low^ NK cell population, four of the animals studied here had negligible levels of NKG2A^high^ NK cells. This different constitutive presence of NKG2A^high^ cells was observed in blood and, at different rates, in lymphoid tissues, including pLN, mLN, BM and spleen (Fig. 4A), and non-lymphoid tissues such as liver, gut and BAL (Fig. 4B). We therefore evaluated possible determinants of the presence or absence of NKG2A^high^ NK cell subsets. To increase the statistical power, we analyzed samples from 16 additional non-infected macaques (Table S2, median age = 3.9 years, IQR 1.88 years). In total, seventeen macaques were characterized by the presence of NKG2A^high^ NK cells, while eleven (termed NKG2A^low^) did not carry the NKG2A^high^ NK cell subset. The abundance of NKG2A^high^ cells did not correlate with the age (*ρ* = −0.01674, *p*-value = 0.9326) nor the weight (*ρ* = −0.2251, *p*-value = 0.9326) of the animals (Fig. S8A). Accordingly, we did not observe any correlation either between the frequency of NKG2A^high^NKp30^−^NKp46^−^ cells and age (*ρ* = 0.08, *p*-value = 0.69) or the weight (*ρ* = 0.07, *p*-value = 0.71) (Fig. S8B).

We then explored the potential impact of the immunogenetic background on the constitutive presence of NKG2A^high^ cells. Due to their restricted diversity, Mauritian CyMs can be classified in a limited number of haplotypes based on microsatellite genotyping and allele-specific PCR^35^. We found the M1 (*p*-value = 0.0491) and M5 haplotypes (*p*-value = 0.0325) were more frequent among animals with the NKG2A^low^ profile, while the M3 haplotype (*p*-value = 0.0238) was more frequent in macaques with the NKG2A^high^ profile (Fig. 4C). We further explored whether these differences could be linked to specific microsatellites in the MHC region that defined the haplotypes. We found that the presence of the 277/278 alleles of the D6S2793 microsatellite, located within the MHC-B gene cluster^36^, was significantly higher in macaques with a NKG2A^low^ profile (*p*-value = 0.0161). In contrast, we observed a trend for 145/146 alleles in the D6S2704 microsatellite, located between the MHC-A and MHC-B region, to be more frequent among macaques with the NKG2A^high^ phenotype (*p*-value = 0.054). When combined, the presence of the 277/278+ D6S2793 allele in absence of 145/146− D6S2704 allele was much higher in NKG2A^low^ macaques (*p*-value = 0.0033), while the opposite (277/278− 145/146+) was observed exclusively in animals with the NKG2A^high^ profile (*p*-value = 0.0233) (Fig. 4D). We did not observe any significant difference regarding other alleles.

Overall, our results indicate that the presence or absence of NKG2A^high^ cells, associated with enhanced post-treatment control, might be constitutively influenced by the MHC-class I-related immunogenetic background of the animals, mirroring the previous observations of NKG2A expression in humans^14^.

### Homing potential of NKG2A^high^ and NKG2A^low^ cells before ATI correlates with post-treatment control

**Fig. 5:**
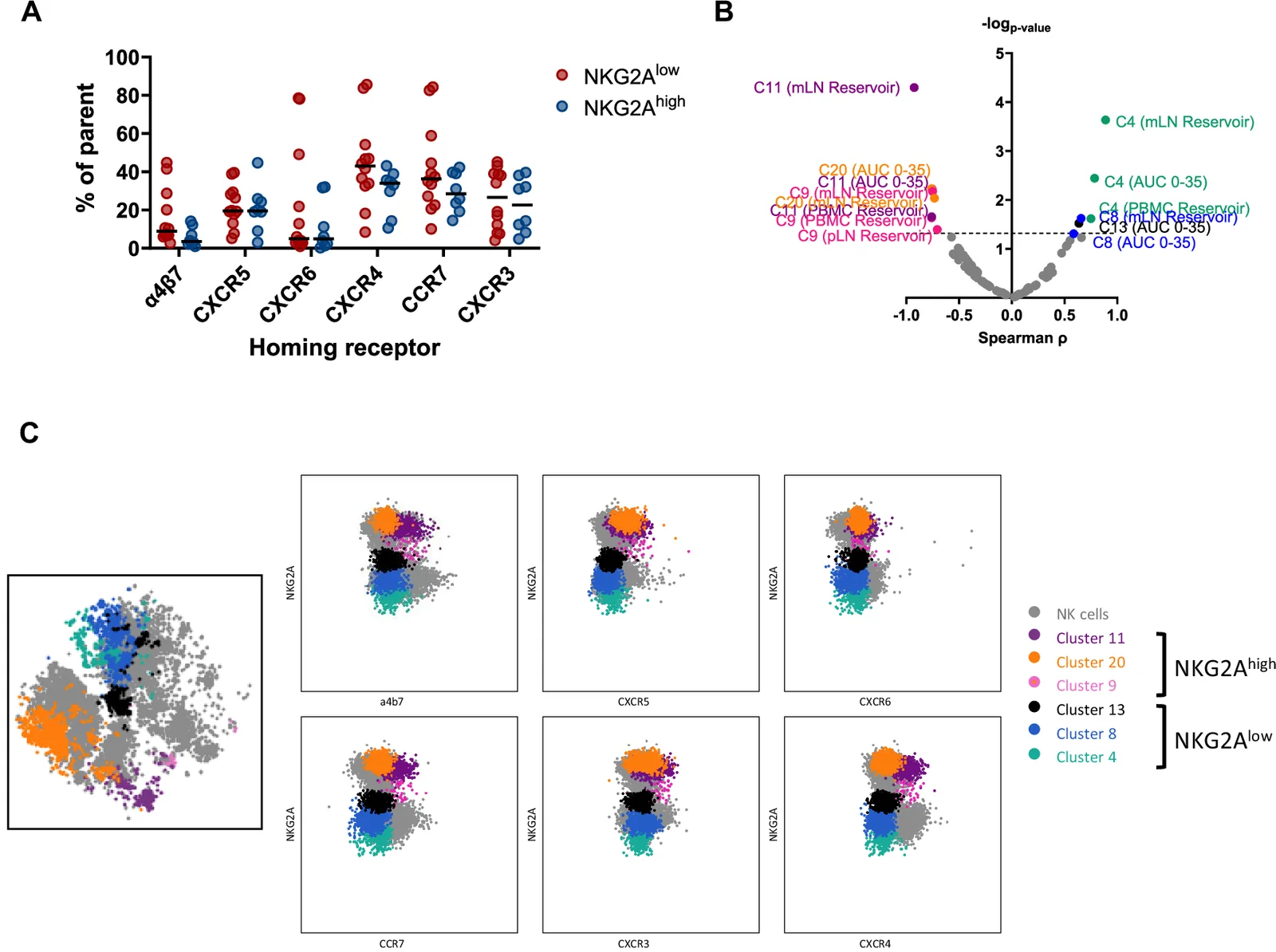
Expression of homing markers in NK cells before ATI (*n* = 12 macaques). **A** Expression of homing markers in NKG2A^low^ and NKG2A^high^ cells. Each data point represents results with samples from one individual macaque (*n* = 12 individuals), horizontal lines represent the median. Dimensional reduction and unsupervised on CD3^−^CD14^−^CD20^−^NKp80^+^ cells acquired before ATI identified 20 different clusters of NK cells based on the expression of NKG2A, Integrin α4β7, CXCR5, CXCR6, CCR7, CXCR3 and CXCR4 of which 6 (Clusters 4, 8, 9, 11, 13 and 20) showed positive or negative correlation with the AUC during ATI and/or total SIV DNA. **B** Volcano plots showing p-value and Spearman ρ of the correlation between cluster abundance in PBMC AUC of viral load during ATI and total SIV DNA copies/10^6^ CD4^+^ T-cells. **C** Expression of homing markers Integrin α4β7, CXCR5, CXCR6, CCR7, CXCR3 and CXCR4 in clusters 4, 8, 9, 11, 13 and 20. Multiple comparison was performed by Two-way ANOVA and False Discovery Rate (FDR) correction (Benjamini, Krieger and Yekutieli). Correlations were assessed using two-sided Spearman’s rank method. Dimensional reduction was performed by Fit-tSNE and clustering by FlowSOM. Significant values were considered when *p*-value < 0.05.

The different relative distribution of NKG2A^high^ and NKG2A^low^ cell subsets in tissues suggest that these cells may possess different trafficking capacities. We therefore analyzed the expression of various homing markers in NKG2A^high^ and NKG2A^low^ NK cells in blood prior to ATI (Fig. S9). No significant differences were found regarding the individual expression of homing markers in each subset (Fig. 5A). Dimensional reduction was set at 4000 NK cells/sample by Fit-tSNE. Twenty clusters were identified in an unsupervised manner by FlowSOM (Elbow Plot determination) based on the expression of NKG2A and homing markers (Integrin α4β7, CXCR5, CXCR6, CCR7, CXCR3 and CXCR4) (Fig. S10). Six clusters (4, 8, 9, 11, 13 and 20, Fig. 5B) showed significant positive or negative correlations with either the cumulated viremia after ATI or the SIV DNA levels in PBMC, pLN or mLN at the end of the study. Clusters 9, 11 and 20 were negatively correlated with cumulated viremia or the viral reservoir after ATI, whereas clusters 4, 8 and 13 were positively correlated with these virological markers. Notably, all NK cell subsets associated with improved viral control after ATI corresponded to NKG2A^high^ cells expressing varying combinations of homing markers (Cluster 9: α4β7, CXCR5, CCR7, CXCR3, CXCR4; Cluster 11: α4β7, CXCR5, CXCR6, CCR7, CXCR3 and CXCR4; Cluster 20: CXCR6 and CXCR3) (Fig. 5C). In contrast, all clusters that positively correlated with markers of viremia and viral reservoir were composed of cells with intermediate or low levels of NKG2A that lacked homing markers (Fig. 5C).

These findings suggest that NK cells associated with post-treatment control are characterized both by NKG2A^high^ expression and homing potential, indicating that the capacity of NKG2A^high^ cells to localize in tissues may contribute to the containment of the viral rebound following ATI.

### Functional profiling of distinct NKG2A^high^ and NKG2A^low^ NK cell subsets

Because of their strong positive and negative association with viral control after ATI, we hypothesized that NKG2A^high^NKp30^-^NKp46^-^ and NKG2A^low^NKp30^+^NKp46^+^ cells may have different functional capacities. We therefore measured at baseline, at week 52 after treatment initiation, and after ATI, the basal content of cytotoxic molecules in NK cells, and their capacity to release these molecules and several soluble factors after 4 h culture with K562 target cells (Fig. S11).

**Fig. 6:**
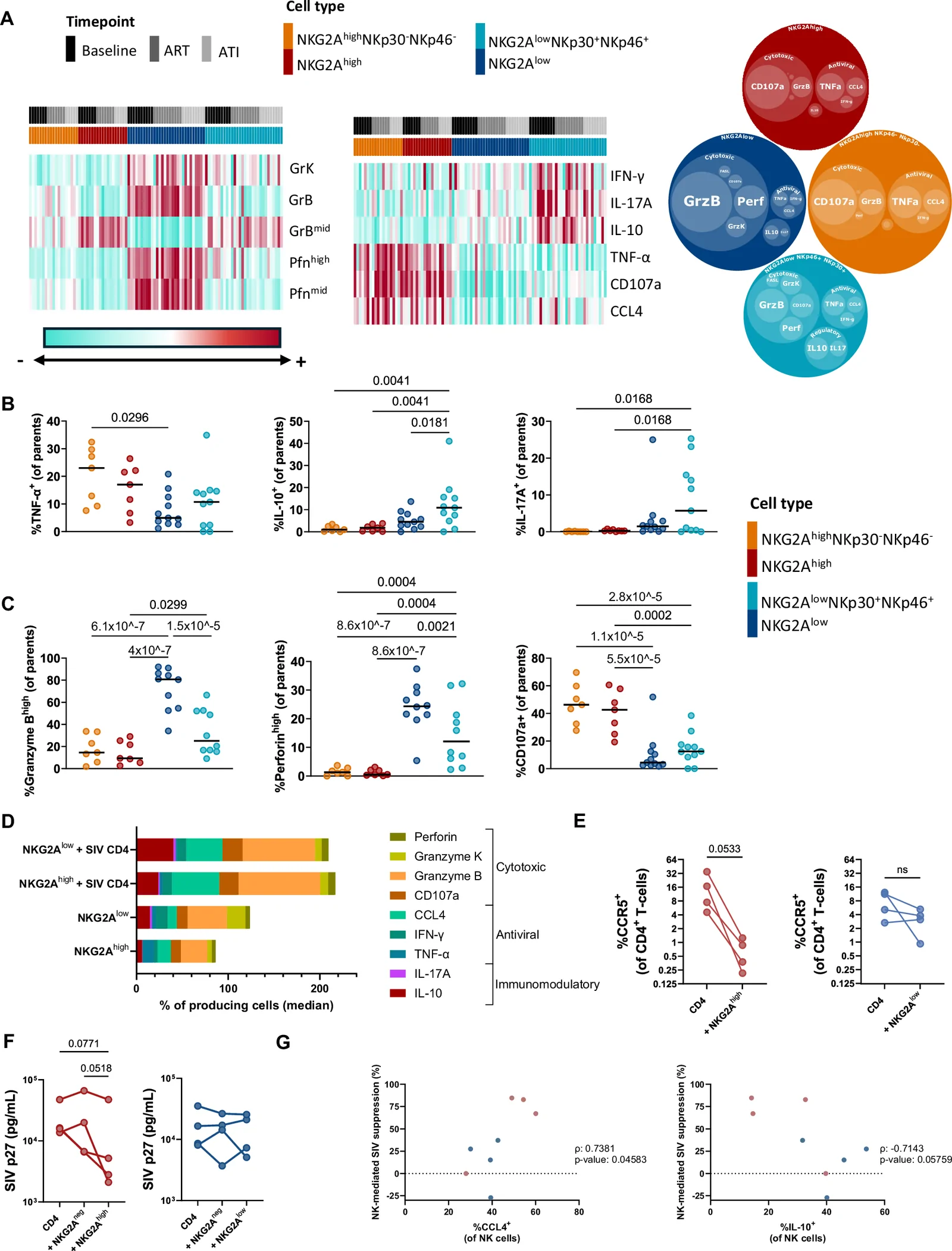
Functional properties of NKG2A^high^, NKG2A^high^NKp30^-^NKp46^-^, NKG2A^low^ and NKG2A^low^NKp30^+^NKp46^+^. **A** Cytotoxic potential of unstimulated NK cells (left) and cytokine production in NK cells stimulated with K562 target cells in an effector:target ratio = 2:1 for 4 h (right) (*n* = 12 macaques). **B** Comparison of TNF-α (top, left), IL-10 (top, middle) and IL-17A (top, right) production in NKG2A^high^, NKG2A^high^NKp30^-^NKp46^-^, NKG2A^low^ and NKG2A^low^NKp30^+^NKp46^+^ (*n* = 12 macaques). Each data point represents results with samples from one individual macaque; horizontal lines represent the median. **C** Granzyme B^high^ (bottom, left) and Perforin^high^ (bottom, middle) and CD107a (bottom, right) production in NKG2A^high^, NKG2A^high^NKp30^-^NKp46^-^, NKG2A^low^ and NKG2A^low^NKp30^+^NKp46^+^ (*n* = 12 macaques). Each data point represents results with samples from one individual macaque; horizontal lines represent the median. **D** NK cell effector functions cultured alone or cocultured with autologous SIV-infected CD4^+^ T-cells (*n* = 8). **E** %CCR5+ of SIV-infected CD4^+^ T-cells alone (CD4) or cocultured with NKG2Ahigh NK cells (+NKG2Ahigh, left, *n* = 4 macaques) or NKG2Alow NK cells (+NKG2Alow, right, *n* = 4 macaques). **F** p27 levels in supernatants of SIV-infected CD4^+^ T-cells cultured alone or cocultured with NKG2A^neg^ NK cells, NKG2A^high^ NK cells (left, *n* = 4 macaques) or NKG2A^low^ NK cells (right, *n* = 4 macaques). **G** Correlation between NK cell-mediated SIV suppression and the percentage of cells producing CCL4 (left) or IL-10 (right) (*n* = 8 macaques). Horizontal lines represent the median. Multiple comparisons were performed by One-way ANOVA and False Discovery Rate (FDR) correction (Benjamini, Krieger and Yekutieli). Correlations were assessed using two-sided Spearman’s rank method. Paired comparisons were made by two-sided Wilcoxon signed-rank test. Significant values were considered when *p*-value < 0.05 and *q*-value < 0.05.

The NKG2A^low^ cells exhibited higher cytotoxic potential compared to NKG2A^high^ cells (as shown by higher expression of perforin, granzymes B and K in basal conditions) (Figs. 6A and S12), which might indicate a more mature phenotype. However, the NKG2A^low^NKp30^+^NKp46^+^ cells carried less frequently perforin and Granzyme K, and had lower Granzyme B content, indicating that these cells represent a specific and comparatively less cytotoxic subset within the NKG2A^low^ population. NKG2A^high^NKp30^-^NKp46^-^ and the broader NKG2A^high^ cells displayed relatively similar cytotoxic profiles, characterized by lower frequencies of Granzyme B^high^/Granzyme K^+^ cells. In contrast, upon co-culture with target cells, NKG2A^high^ and NKG2A^high^NKp30^-^NKp46^-^ cells showed a higher capacity to degranulate and to produce TNF-α than NKG2A^low^ and NKG2A^low^NKp30^+^NKp46^+^ cells (Fig. 6B), which were characterized, in particular the latter, by the production of higher levels of IL-17A and IL-10 in response to target cells (Fig. 6C). The different functional profiles of the NK cell subsets were defined since the baseline and no major changes were observed at the three timepoints analyzed (Baseline, week 52 on ART or week 4 after ATI) except a trend for further loss of granzyme B and perforin content in NKG2A^low^NKp30^+^NKp46^+^ upon infection when compared to the total NKG2A^low^ compartment (Fig. S12).

Our analysis identified profound functional differences between the NKG2A^high^ and the NKG2A^low^ compartments. Moreover, NKG2A^low^NKp30^+^NKp46^+^ cells showed, since the baseline, decreased cytotoxic potential and elevated capacity to produce IL-17A and IL-10 in response to target cells, distinguishing them from the broader NKG2A^low^ population, and this dysfunctionality was exacerbated by infection. In contrast, NKG2A^high^NKp30^-^NKp46^-^ NK cells were highly reactive, and their functionality was not impacted by infection.

To more directly evaluate the anti-SIV potential of NKG2A^high^ and NKG2A^low^ NK cells, we cocultured these sorted NK-cell populations with autologous CD4^+^ T cells infected with SIVmac251 in vitro, and measured SIV p27 levels, CD4^+^ T-cell activation and CCR5 expression, and NK cell effector molecules (Fig. S14). Several antiviral and immunomodulatory readouts (including CCL4, granzyme B, CD107a, perforin, and IL-10) increased in the presence of SIV-infected CD4^+^ T cells. Compared with NKG2A^low^ NK cells, NKG2A^high^ NK cells produced higher levels of CCL4 and lower levels of IL-10 (Fig. 6D), in line with the previously observed response to K562 target cells. Coculture with both NK cell subsets induced the expression of HLA-DR on CD4^+^ T cells, but only NKG2A^high^ cells induced Ki-67 on CD4^+^ T cells (Fig. S15). Notably, CD4^+^ T cells cocultured with NKG2A^high^ NK cells, but not with NKG2A^low^ cells, showed a marked reduction in CCR5 expression (Fig. 6E).

Co-culture of SIV-infected CD4^+^ T cells with NKG2A^high^ NK cells consistently led to lower p27 production than control conditions (CD4^+^ T cells alone) (Fig. 6F), while no difference was observed between CD4^+^ T cells cultured alone and those cocultured with either NKG2A^neg^ or NKG2A^low^ NK cells. Overall, NK-cell–mediated viral suppression was positively associated with the capacity of NKG2A^high^ and NKG2A^low^ NK cells to produce CCL4 (ρ = 0.7381, *p* = 0.04583) (Fig. 6G) and negatively with their capacity to produce IL-10 (ρ = −0.7143, *p* = 0.05759).

In summary, functional differences between NK cell subsets were consistently observed in response to K562 target cells and autologous infected CD4^+^ T cells and resulted in a higher anti-SIV activity by NKG2A^high^ cells. Our results suggest that this may be related, at least in part, to β-chemokine–mediated downregulation of CCR5.

## Discussion

In this study, we have identified two NK cell subsets which relative abundance in CyMs before SIVmac251 infection was associated with the outcome of ART interruption more than 2 years later. We exposed genetic influences, phenotype, trafficking potential, and effector functions of these cell subsets that may help to understand the factors underlying post-treatment control of SIV infection in this model. Our results suggest that constitutive characteristics of NK cells influence the acquisition of PTC status.

The two NK cell subsets characterized here differ in their expression of NKG2A, NKp30, and NKp46, and show opposite associations with post-treatment control. While NK-cell antiviral activity is shaped by a broader set of activating and inhibitory receptors, including DNAM-1^31^ and NKG2D^37^, we did not find evidence in this study that these markers constitutively influenced the outcome after ATI. In contrast, NK cells expressing high levels of NKG2A were associated with improved post-treatment control. NKG2A is an inhibitory receptor, typically expressed on immature NK cells, that dampens NK cell activity upon interaction with its ligand, MHC-E, on target cells. However, NKG2A contributes to NK cell education, and both its expression level and the functional reactivity of NKG2A^+^ NK cells are influenced by leader peptides from conventional MHC class-I molecules, which modulate MHC-E surface expression. Intriguingly, the presence of NK cells expressing high levels of NKG2A was restricted to a subset of animals in the study. This pattern appeared to be shaped by the MHC background of the animals. Indeed, the absence of MHC M1 and/or M5 haplotypes, or the presence of M3, was associated with the detection of the NKG2A^high^ NK cell subset. We further traced this influence to specific allelic variants within two microsatellite loci in the MHC class-I region, although a mechanistic link remains to be formally established. It is worth noting that we have recently identified an immunogenetic fingerprint (HLA-B*35 Bw4TTC2) that is overrepresented among HIV-1 PTC from the VISCONTI cohort^13^. This genotype is associated with NK cell education and characterized by homozygosity for a threonine at the −21 residue of the leader sequence of HLA-B alleles, associated with low HLA-E surface expression and, consequently, higher expression of NKG2A^14^. Combined, these observations support that NK cells, in particular through the NKG2A axis, play a key role in post-treatment control of infection.

On the other hand, the higher frequency of NKp46^-^NKp30^-^ NK cells may reflect a less inflamed environment, a finding consistent with recent observations in PTCs from the pVISCONTI study, where reduced inflammation was associated with preserved intestinal homeostasis^38^. NKp30 and NKp46 are activating receptors expressed by NK cells that promote cytotoxicity upon engagement with self or pathogen-induced ligands^39^, and their expression is enhanced by pro-inflammatory cytokines such as IL-12, IL-15 and IL-18^40^ or type-I interferons. This may explain the distinct trajectories of NKG2A^high^NKp30^−^NKp46^−^ and NKG2A^low^NKp30^+^NKp46^+^ NK cells, with the former declining in blood, pLN and BM upon infection, whereas the latter accumulated in all these compartments during acute infection. In this context, the early initiation of ART may have contributed to preserve the NKG2A^high^NKp30^−^NKp46^−^ NK cell subset. A partial recovery of this population was observed in the tissues, particularly in pLN, but not in blood, after 2 years of ART, and their frequency in lymphoid tissues prior to ATI inversely correlated with the size of the SIV reservoir in pLN/mLN. This may reflect the capacity of the cells to traffic to infected tissues. Although NKG2A^low^ and NKG2A^high^ cells did not differ significantly in their expression of chemokine receptors, we observed a negative correlation between the homing potential of NKG2A^high^ cells and viremia during ATI, suggesting that tissue localization is critical. Notably, specific subsets of NKG2A^high^ cells expressed CXCR5 and CCR7, consistent with the potential to infiltrate lymph nodes and B follicles.

We also found profound differences in the effector capacities of NKG2A^low^ and NKG2A^high^ NK cells. NKG2A^low^ NK cells possessed a broad cytotoxic arsenal, carrying higher levels of both soluble (Granzyme B, Granzyme K, Perforin) and membrane-bound (FasL) cytolytic molecules. However, the NKG2A^low^NKp30^+^NKp46^+^ subset displayed significantly lower levels of Perforin and Granzyme at baseline compared to the broader NKG2A^low^ compartment, and these levels were further reduced upon infection. NKG2A^low^NKp30^+^NKp46^+^ cells also showed limited responsiveness to target cells and produced IL-17A and IL-10 in a bystander manner. By contrast, NKG2A^high^ NK cells were characterized by the production of antiviral cytokines (TNF-α, CCL4) and exhibited stronger degranulation upon stimulation with target cells. NKG2A^high^NKp30^−^NKp46^−^ NK cells shared a similar functional profile with the other NKG2A^high^ NK cells, suggesting that the former is the reflection of a particular immunological setting, and their function was not affected by SIV infection. The cytokine-producing profile of these cells is consistent with the NK cells residing in secondary lymphoid tissues in healthy and SIV-infected macaques, rather than the highly cytotoxic subsets typically found in blood and mucosal tissues^41^. Therefore, NKG2A^low^NKp30^+^NKp46^+^ NK cells represent a functionality-limited NK cell subset that is further negatively impacted by infection, reflecting the impaired NK cell response previously reported in HIV/SIV infections^42–44^. In contrast, NKG2A^high^NKp30^-^NKp46^-^ cells represent a subset in which functional integrity is preserved despite infection. Although we were limited by the available samples to test, similar functional differences were observed when sorted NKG2A^high^ or NKG2A^low^ NK cell subsets were cultured ex vivo with autologous SIV-infected CD4^+^ T-cells, and in this setting, NKG2A^high^ NK cells showed stronger direct antiviral activity.

Nevertheless, the exact mechanism that links NKG2A^high^NKp30^-^NKp46^-^ NK cells with post-treatment control remains to be established. Consistent with our observations, several studies have shown that NKG2A^+^ cells possess an enhanced capacity to respond to autologous HIV-infected CD4^+^ T cells^45^, particularly in the case of −21T homozygous individuals^46^, and that elite controllers carry particularly high frequencies of NKG2A^+^ NK cells^43,47^. Moreover, in African green monkeys, natural hosts of SIV, NK cell responses within lymph nodes are mediated through the MHC-E/NKG2A axis^24^. Therefore, it is possible that NKG2A^high^ cells can migrate to lymph and recognize HIV- or SIV-infected cells through MHC-E-dependent (via antigen presentation^48,49^ or the “missing-self” axis upon MHC-E downregulation)^50^ or independent mechanisms, leading to the production of antiviral soluble molecules such as TNF-α and CCL4^19^. Our coculture assays point to a relevant role of the β-chemokine signature of NKG2A^high^ NK cells, as higher CCL4 production was accompanied by reduction in CCR5 expression on CD4^+^ T cells, which could reflect β-chemokine-induced CCR5 internalization and/or downmodulation^51,52^, thereby potentially limiting viral entry and/or spread, and it was indeed associated with enhanced antiviral activity by this NK cell subset. However, the exact mechanisms linking the NKG2A/MHC-E axis to NKG2Ahigh NK cells' control of viral infection still need to be deciphered.

In addition to a direct antiviral role, NK cells may contribute to the orchestration of other immune responses. We previously reported that SIV PTC from the pVISCONTI study were characterized by the presence of enhanced CD8 T-cells with memory traits in secondary lymph nodes, capable of exerting a superior antiviral pressure to counteract viral rebound after ATI^25^. β-chemokines produced by NK cells could promote the recruitment of T-cells to the site of infection^53^. Moreover, reduced expression of NKp30, and at a lesser extent NKp46, may protect dendritic cells from NK cell-mediated killing^54^, thereby favoring the priming of such CD8^+^ T cells. In contrast, IL-10 producing-NKG2A^low^NKp30^+^NKp46^+^ NK cells may contribute to dendritic cell depletion^55^, CD8^+^ T cell dysfunction, and to the establishment and persistence of the SIV reservoir^56^.

Overall, these findings suggest that a superior MHC-driven NKG2A^high^ vs NKG2A^low^ balance, coupled with low baseline inflammation, sets a favorable stage for an improved response against SIV/HIV during ART interruption. They further indicate that the early preservation of specific NK cell subsets through therapeutic intervention may be critical for controlling viral rebound following ATI. Together, these results underscore the pivotal role of the NKG2A pathway in shaping NK cell responses during infection and treatment and highlight the potential of harnessing this axis in novel therapeutic strategies aimed at achieving durable HIV remission.

## Methods

### Ethic statement

Male Cynomolgus macaques *(Macaca fascicularis)* of Mauritian geographic origin were housed at the Infectious Disease Models and Innovative Therapies (IDMIT) center (CEA site, Fontenay-aux-Roses, France). All nonhuman primate research at IDMIT adhered to French national regulations under the oversight of veterinary inspectors (CEA Permit No. D92-032-02) and met the Office for Laboratory Animal Welfare’s care and use standards (Assurance Nos. A5826-01 and F20-00448). Procedures complied with European Directive 2010/63 (Recommendation No. 9). The pVISCONTI study received approval from the Comité d’Éthique en Expérimentation Animale du CEA (statement A15 035) and was registered with the French Ministry of Education and Research (No. 2453-2015102713323361v3). Animal handling, viral inoculations, and sample collection occurred under ketamine chlorhydrate sedation (Imalgene 1000®, 10 mg/kg IV, Merial). Tissues were obtained during follow-up and at necropsy. Euthanasia followed initial ketamine sedation with an intravenous bolus of sodium pentobarbital (Doléthal, 180 mg/kg, Laboratoire Vetoquinol).

### Animal and experimental conditions

This study includes a subgroup of 12 male CyMs (6 classified as PTCs and 6 as PTNCs) from the pVISCONTI study^25^ (median age = 4.73 years, IQR = 0.93 years), which were dedicated to in-depth immunological characterization (Table S1). Infection was performed at an animal infectious dose (AID)_50_ of 1000 by intravenous inoculation with uncloned SIVmac_251_ kindly provided by Anne-Marie Aubertin (Université Louis Pasteur, Strasbourg, France)^57^.

ART containing emtricitabine (FTC, 40 mg/kg, Gilead), dolutegravir (DTG, 25 mg/kg, ViiV Healthcare) and tenofovir-disoproxil-fumarate (TDF, 5.1 mg/kg, Gilead) was coformulated as a daily subcutaneous injection. ART was initiated either at week 4 (*n* = 6) or week 24 (*n* = 6) and was continued for 24 months before following ATI. Animals were classified as PTC if they reached at least one pVL<400 SIV RNA copies/mL (similar to the viral load threshold for PTCs in the HIV-1 VISCONTI cohort^2^) after viral rebound or if they did not experience viral rebound. The endpoint was 48 weeks after ATI, except for PTNC showing progression to disease before this time. All animals were monitored for at least 6 months post ATI.

### Blood collection and processing

Venous blood was drawn into either Vacutainer Plus Plastic K3EDTA Tubes or Vacutainer CPT Mononuclear Cell Preparation Tubes containing sodium heparin (BD Biosciences). Plasma was isolated from the K3EDTA by centrifugation for 10 min at 480 g. PBMCs were isolated from the CPT tubes following the manufacturer’s instructions (BD Biosciences), and residual red blood cells were lysed in ammonium-chloride-potassium (ACK) buffer (0.15 M NH₄Cl, 10 mM KHCO₃, 0.1 mM EDTA, pH 7.4).

### Tissue collection and processing

Samples from axillary or inguinal lymph nodes (peripheral pLNs), bone marrow (BM), and bronchoalveolar lavage (BAL) were collected over time. At necropsy, the spleen, mesenteric lymph nodes (mLNs), colon, and liver were also harvested. All tissues were kept in RPMI medium at 2–8 °C. Lymph node cells were obtained by mechanically dissociating the nodes with a gentleMACS Dissociator (Miltenyi Biotec), filtering the suspension through a 70 µm mesh, then lysing red blood cells in ACK buffer. Bone marrow cells were isolated using Lymphocyte Separation Medium (Eurobio Scientific) diluted to 90% in DPBS, centrifuged for 20 min at 350 × *g*, and residual erythrocytes removed by ACK lysis. Spleen cells underwent a similar process: mechanical disruption in RPMI with the gentleMACS Dissociator, density separation as for BM cells, and ACK-mediated red cell lysis. For colonic lymphocytes, mucosa from roughly 10 cm of colon was extensively washed in PBS and R10 (RPMI with 10% fetal calf serum and penicillin/streptomycin), then digested for 45 min with collagenase II before mechanical dissociation; lymphocytes were finally isolated over a 67/44 Percoll gradient (Sigma-Aldrich). Liver tissue was likewise dissociated mechanically, the suspension sequentially filtered through 300 µm, 100 µm, and 70 µm meshes, and lymphocytes recovered via an OptiPrep gradient (Sigma-Aldrich).

### Quantification of plasma viral load

Longitudinal monitoring of plasma viral load was performed by quantitative RT-PCR (limit of detection = 12.3 SIV RNA copies/mL). Viral RNA was prepared from 100 μL of cell-free plasma. The assay was performed using a SuperScript III Platinum One-Step qRT-PCR Kit (Thermo Fisher Scientific) with a CFX96 Touch Real-Time PCR Detection System (Bio-Rad). Each tube contained 12.5 μL of 2× reaction mixture, 0.5 μL of RNaseOUT (40 U/μL), 0.5 μL of Superscript III Reverse Transcriptase/Platinum Taq DNA Polymerase, 1 μL of each primer (125 μM), 0.5 μL of the fluorogenic probe (135 μM), and 10 μL of eluted RNA. Primer/probe sequences were designed to amplify a region of the SIVmac_251_ gag gene. The forward (F) primer sequence was 5′-GCAGAGGAGGAAATTACCCAGTAC-3′ (24 bp), and the reverse (R) primer sequence was 5′-CAATTTTACCCAGGCATTTAATGTT-3′ (25 bp). The probe sequence was 5′-FAM-TGTCCACCTGCCATTAAGCCCGA-BHQ1-3′ (23 bp). This probe had a fluorescent reporter dye, FAM (6-carboxyfluorescein), attached to its 5′ end and a quencher, BHQ1 (black hole quencher 1), attached to its 3′ end (TaqMan, Applied Biosystems). Samples were heated for 30 min at 56 °C and 5 min at 95 °C, followed by 50 thermocycles, each comprising 15 s at 95 °C and 1 min at 60 °C.

### Quantification of SIV-DNA

Total DNA was extracted from purified CD4^+^ T-cells from PBMC, pLN or mLN cells with an AllPrep DNA/RNA Mini kit (Qiagen). Two or three samples were used per tissue and disrupted separately with a MagNA Lyser (GmbH, Roche Diagnostics, Brussels, Belgium) to minimize the differences in viral distribution within an organ. The total SIV-DNA was quantified by ultrasensitive real-time PCR targeting the gag region^58^. The cycling conditions were 2 min at 50 °C and 10 min at 95 °C, followed by cycles at 95 °C for 15 s and 60 °C for 1 min. The limit of detection was 1 copy/PCR. To normalize SIV-DNA per million cells, the CCR5 gene was quantified by real-time PCR with the same cycling conditions described above (forward primers: an equimolar mix of the primers 5′-CAACATGCTGGTCGATCCTCAT-3′ and 5′-CAACATACTGGTCGTCCTCA TCC-3′, reverse primer: 5′-CAGCATAGTGAGCCCAGAAG-3′, probe: 5′-HEX-CTGACA TCTACCTGCTCAACCTG-BHQ1-3′). For both PCRs, the reaction volumes were 50 μL, containing 25 μL of 2× qPCR Mastermix Plus (Eurogentec, Seraing, Belgium), 0.4 µM each primer and 0.2 µM probe. SIV-DNA levels were reported as copies per 10^6^ CD4^+^ T cells or as threshold values when the SIV-DNA level was under the detection threshold, or SIV-DNA was not detected.

### MHC genotype

The MHC genotype was determined with 20 microsatellites scattered across the MHC region as described before^59^. The microsatellite markers and primer sequences are described in Table S3. Genotypes were determined using DNA Size-kit-600 *Beckman Coulter) after denaturation and separation of the amplification products by capillary electrophoresis using a CEQ800 analyser and scored with the software CEQ8000 Genetic Analysis System v8.0 (Beckman Coulter).

### Analysis of NK cell phenotype

NK cell phenotype was assessed longitudinally using fresh PBMCs and tissue cell suspensions. Cells were first stained with the LIVE/DEAD Fixable Aqua Dead Cell Stain Kit (Thermo Fisher Scientific) and then stained for surface expression of: anti-CD3 V450 (Clone SP34-2, BD Biosciences), anti-CD14 V450 (clone M5E2, BD Biosciences), anti-CD20 V450 (Clone L27, BD Biosciences), anti-HLA-DR Pacific Blue (clone G46-6, BD Biosciences), anti NKG2A FITC (Clone REA110, Miltenyi), anti-NKp46 PC7 (Clone BAB281, Beckman Coulter), anti-CD8 PerCP-Vio700 (Clone BW135/80, Miltenyi), anti-CD56 PE-CF594 (Clone NCAM16.2, BD Biosciences), anti-NKp30 PE (Clone AF29-4D12, Miltenyi), anti-CD69 APC-Cy7 (Clone FN50, Biolegend), anti-CD16 AF700 (Clone 3G80, Biolegend), anti-NKp80 APC (Clone 4A4.D10, Miltenyi). A second panel was used longitudinally to further assess NK cell phenotype. NK cells were stained LIVE/DEAD Fixable Aqua Dead Cell Stain kit and anti-CD3, anti-CD20, anti-CD14, anti-HLA-DR, anti-NKp80, anti-CD16, anti-CD8 and anti-CD56 and anti-CD215 FITC (Clone eBioJM7A4, Thermofisher), anti-NKG2D PE-Vio770 (Clone BAT221, Miltenyi), anti-DNAM-1 PE (Clone DX11, Miltenyi) and anti-CD161 APC-Cy7 (Clone HP3G10, Biolegend). To evaluate the homing potential, cells were stained with anti-CD3, anti CD14, anti-CD20, anti-HLA-DR, anti-NKG2A and anti-NKp80 as above, and anti-CXCR4 PECy7 (Clone 12G5, BD), anti-CXCR3 PerCP-Cy5.5 (Clone 1C6, BD), anti-CCR7 PE-Dazzle594 (Clone G043H7, BioLegend), anti-CXCR6 PE (Clone 568, R&D), anti- α4/β7 APC-Fire750 (Clone FIB504, BioLegend) and anti-CXCR5 APC-R700 (Clone RF8B2, BD). Cells were then fixed with PFA 4% solution and acquired using an LSRII flow cytometer (BD Biosciences). NK cells were gated as Viable+Lineage-(CD3-CD14-CD20-HLADR-)NKp80+^26–28^.

### Analysis of NK cell function

NK cells were isolated from 10 million cryopreserved PBMCs by negative magnetic bead separation using a custom NHP NK cell isolation kit (Miltenyi) (Fig. S1). This custom NHP NK cell isolation kit was developed in collaboration with Miltenyi Biotec and is available from Miltenyi Biotec upon request. After 2 h of resting at 37 °C, 200,000 NK cells were seeded in a 96-well culture plate with/without 50,000 K562 target cells (human, female; ATCC, Cat# CCL-243) (effector:target ratio = 2:1) for 4 h in the presence of Brefeldin A (5 μg/mL, Sigma-Aldrich) and GolgiStop (1 μL/mL, BD Biosciences) and anti-CD107a BV421 (Clone H4A3, Biolegend). Viability was determined by the Fixable Viability Stain 570 (BD Biosciences). Extracellular staining was performed as described above, including anti-FasL BUV737 (Clone NOK-1, BD Biosciences). Cells were fixed and permeabilized (Fix&Perm Cell Permeabilization Kit, Thermofisher) and stained anti-CCL4 AF350 (Clone 24006, R&D), anti-TNF-α BV785 (Clone MAb11, Biolegend), anti-IL-10 BV650 (Clone JES3-9D7, BD Biosciences), anti-Granzyme-B BV510 (Clone GB11, Biolegend), anti-Granzyme K PerCP-eFluor710 (Clone G3H69, Thermofisher), anti-Perforin BV711 (Clone dG9, Biolegend), anti-IL-17A PE-Vio615 (Clone CZ8-23G1, Miltenyi) and anti-IFN-γ Spark NIR 685 (Clone 4S.B3, Biolegend). Samples were acquired in a Sony ID7000 spectral analyzer.

### Analysis of NK cell SIV suppressive capacity

Approximately 10^6^ CD4^+^ T-cells were purified from 20 M cryopreserved spleen cells by positive selection (CD4 Microbeads non-human primate, Miltenyi) and stimulated for 3 days with concanavalin A (5 μ/mL, Sigma-Aldrich) and IL-2 (100 U/mL, Miltenyi). On day 3, additional 20 M cryopreserved spleen cells were thawed and stained for viability, anti-CD3, anti-CD20, anti-CD14, anti-HLA-DR, anti-NKp80, and anti NKG2A as described before. 1.5 × 10^5^ NKG2A^neg^, NKG2A^high^ or NKG2A^low^ cells were sorted in a FACSymphony S6. Superinfection was carried in 5 × 10^4^ CD4^+^ T-cells in U-bottom 96-wells plates with SIVmac_251_ in the presence (1:1 effector-to-target-cell ration) of autologous NK cell subsets by spinoculation for 1 h (1200 × *g* at room temperature) and incubated for 1 h at 37 °C. Cells were then washed and cultured in R10 medium containing IL-2 (100 U/mL, Miltenyi). The antiviral activity was calculated by measuring the levels of p27 in the supernatant as day 3 by using an SIV p27 Antigen ELISA kit (Xpress Bio) and determined as % of p27 levels in the CD4-NK cell coculture compared to CD4 alone. Cells were then exposed for 16 h to Brefeldin A, Monensin, and anti-CD107a PE-Cy7 (Clone H4A3, BD Biosciences) in order to evaluate NK cell function at day 4. Viability was determined by the Fixable Viability Stain 780 (BD Biosciences). Extracellular staining included anti-CD3 BUV395 (Clone SP34-2, BD Biosciences), anti-CD14 V450 (clone M5E2, BD Biosciences), anti-CD20 V450 (Clone L27, BD Biosciences) anti-CD4 RB545 (Clone L200, BD Biosciences), anti NKG2A FITC (Clone REA110, Miltenyi), anti-NKp46 PC7 (Clone BAB281, Beckman Coulter), anti-NKp30 PE (Clone AF29-4D12, Miltenyi), anti-NKp80 APC (Clone 4A4.D10, Miltenyi), anti-CCR5 RB613 (Clone 3A9, BD Biosciences) and anti-HLA-DR RY703 (Clone L203.rMAb, BD Biosciences). Cells were fixed and permeabilized (Fix&Perm Cell Permeabilization Kit, Thermofisher) and stained anti-CCL4 R718 (Clone D21-1351, BD Biosciences), anti-TNF-α BV786 (Clone MAb11, Biolegend), anti-IL-10 BV650 (Clone JES3-9D7, BD Biosciences), anti-Granzyme-B BV510 (Clone GB11, Biolegend), anti-Granzyme K PerCP-eFluor710 (Clone G3H69, Thermofisher), anti-IL-17A PE-Vio615 (Clone CZ8-23G1, Miltenyi) and anti-IFN-γ RB670 (Clone B27, BD Biosciences). Samples were analyzed in a Sony ID7000 spectral analyzer.

### Data analysis, statistics and visualization

Flow cytometry data were analyzed with OMIQ software (Dotmatics). Normalization was performed on the longitudinal staining by CyCombine in order to minimize batch effects. High-dimensional data were analyzed with Qlucore software. Graphs and statistical analysis were performed using Prism v10.4 (GraphPad Software) or R. *χ*^2^ analysis was used to evaluate absolute frequencies of MHC variants. Multiple comparisons were performed by two-way ANOVA with *post-hoc* false discovery rate correction (FDR). Correlation analysis was performed by non-parametric Spearman rank test. Statistical significance was established at *p*-value < 0.05 and *q*-value < 0.1. Flourish was used for data visualization.

### Reporting summary

Further information on research design is available in the Nature Portfolio Reporting Summary linked to this article.

## Supplementary information

Supplementary Information

Reporting Summary

Transparent Peer Review file

## Source data

Source Data

## Data Availability

The data that support the findings of this study are presented in the main figures and supplemental material of this article. Source data for all figures are provided with this article. Further information and requests for resources and reagents should be directed to the lead contact. Source data are provided with this paper.
