# Supplementary material for: Constitutive natural killer cell features associated with post-treatment control of SIV infection in the pVISCONTI study
Source: Nat Commun. 2026 Aug 22;17:10059. doi: 10.1038/s41467-026-76699-7 (PMC13594178; doi:10.1038/s41467-026-76699-7)
Supplement: Supplementary file 1 [file 41467_2026_76699_MOESM1_ESM.pdf]

Supplementary material

Figure S1. Validation of the custom NHP NK isolation kit.

Figure S2. Gating strategy for NKG2A^{high} cells and NKG2A^{low} on NKp80^{+} cells

Figure S3. Longitudinal phenotyping of additional receptors on NK cells

Figure S4. NKG2A^{high}NKp30^{-}NKp46^{-} and NKG2A^{low}NKp30^{+}NKp46^{+} dynamics in macaques

Figure S5. NKG2A^{high}NKp30^{-}NKp46^{-} and NKG2A^{low}NKp30^{+}NKp46^{+} dynamics as % of total live cells.

Figure S6. Abundance of NKG2A^{high}NKp30^{-}NKp46^{-} and NKG2A^{low}NKp30^{+}NKp46^{+} in different tissues at euthanasia

Figure S7. Frequency of NKG2A^{high}, NKp46^{+} and NKp30^{+} cells during the study in PTC and PTNC at the baseline, during acute infection and before ATI.

Figure S8. Correlation between the frequency of NKG2A^{high} cells and NKG2A^{high}NKp30^{-}NKp46^{-} at the baseline with age and weight

Figure S9. Gating strategy for homing markers on NKG2A^{high} cells and NKG2A^{low}.

Figure S10. tSNE and expression of homing markers in NK cells before ATI.

Figure S11. Gating strategy for cytokine and cytotoxic response to target cells regarding NKG2A expression.

Figure S12. Comparison of IFN-γ, CCL4, FasL, Granzyme K and Granzyme B^{low} and Perforin^{low} levels in NKG2A^{high}, NKG2A^{high}NKp30^{-}NKp46^{-}, NKG2A^{low} and NKG2A^{low}NKp30^{+}NKp46^{+}.

Fig. S13. Comparison of Granzyme B^{high} and Perforin^{high} levels in NKG2A^{low} and NKG2A^{low}NKp30^{+}NKp46^{+} cells over time.

Fig. S14. Gating strategy for NK cell function and CD4^{+} T-cell activation in the coculture system.

Fig. S15. Changes in activation markers on $CD4^+$ T cells.

Table S1. Characteristics of Cynomolgus macaques included in the sub-study

Table S2. Characteristics of additional Cynomolgus macaques included for MHC analyses

Table S3. Primers used to amplify the 20 MHC microsatellites

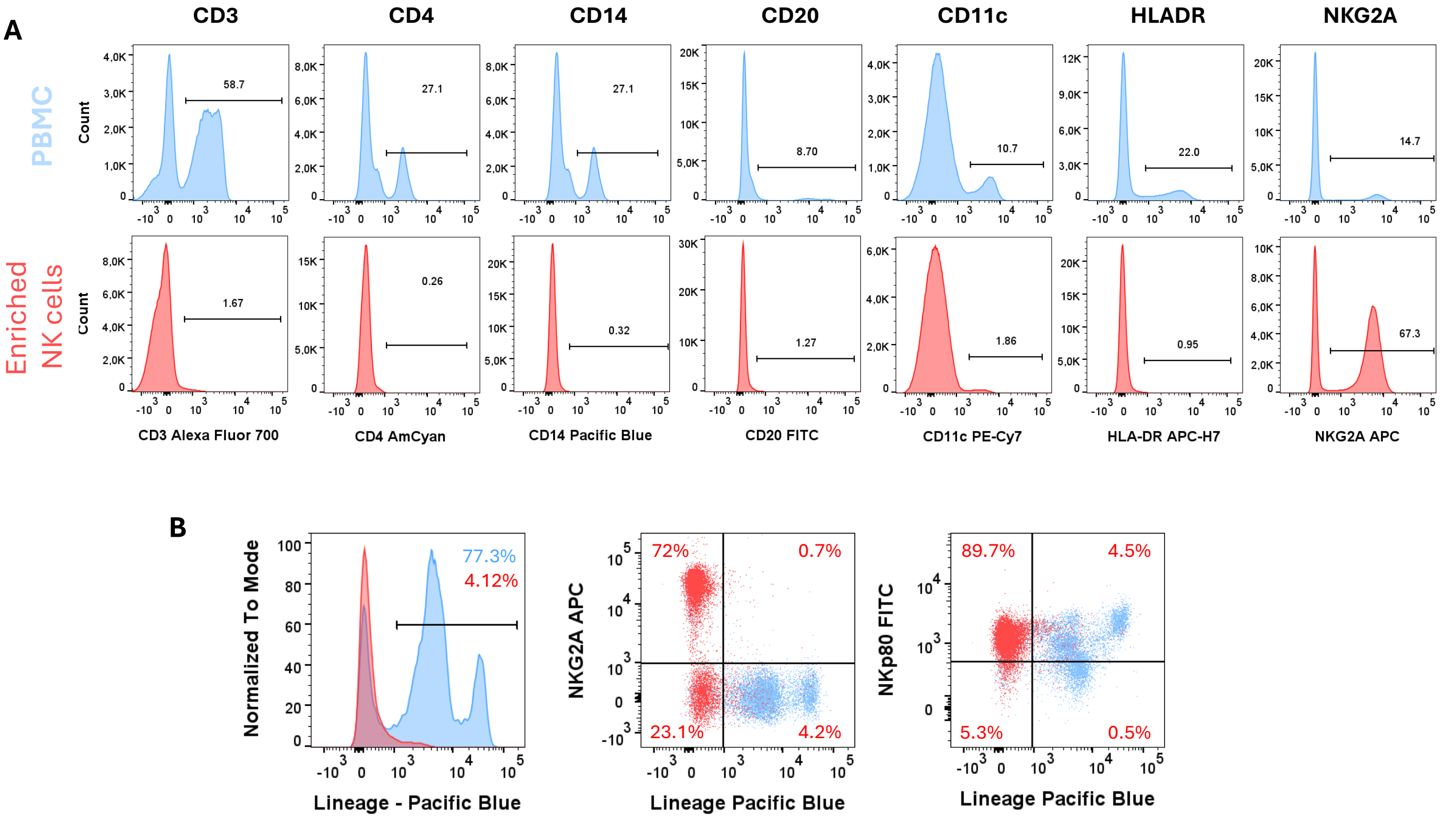

Figure S1. Efficacy of the custom NHP NK isolation kit. A custom kit for NK cell enrichment adapted to NHP was developed with Miltenyi Biotec

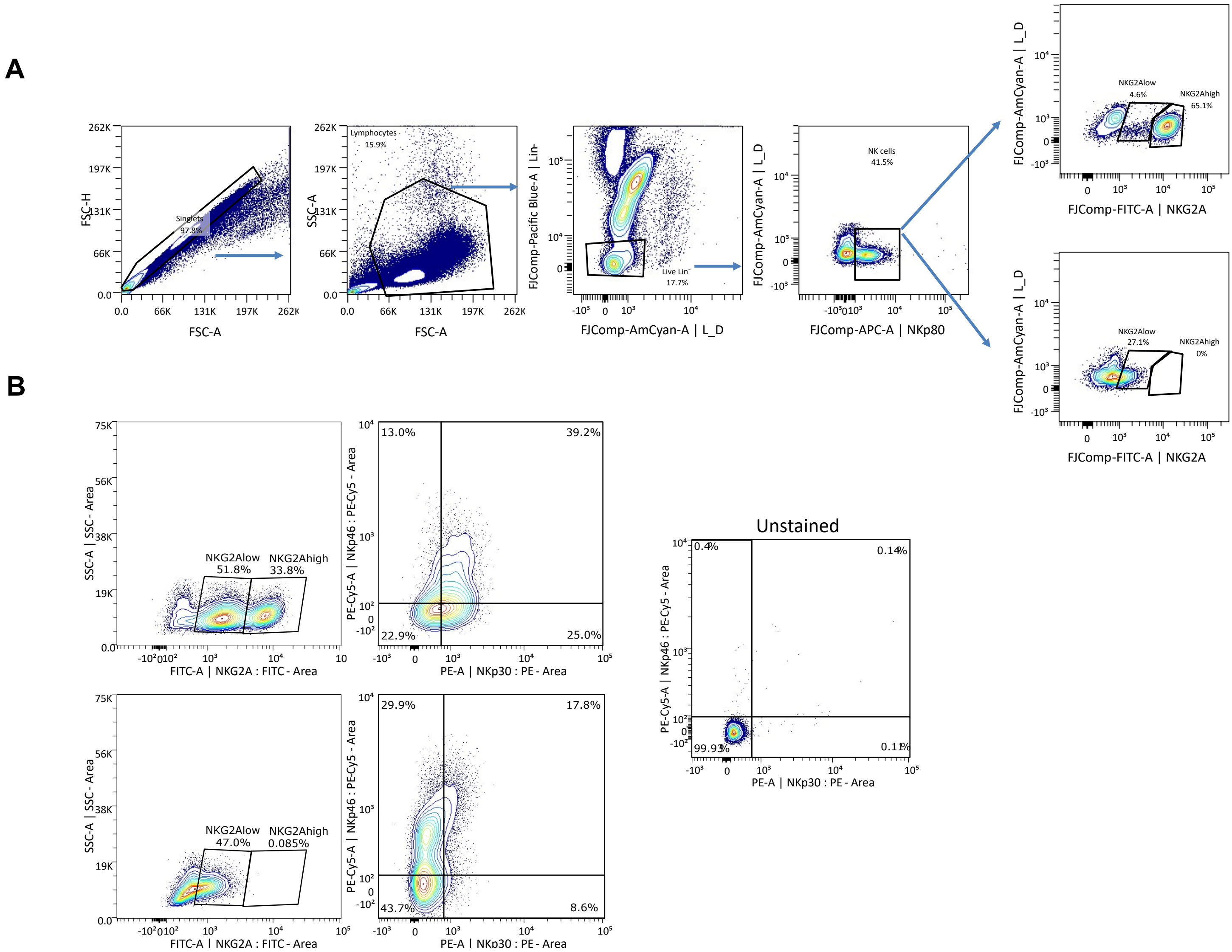

Figure S2. (A) Gating strategy for NKG2A^{high} cells (right, top) and NKG2A^{low} (right, bottom) on NKp80+ cells. (B) NK cells unstained for NKp30 and NKp46.

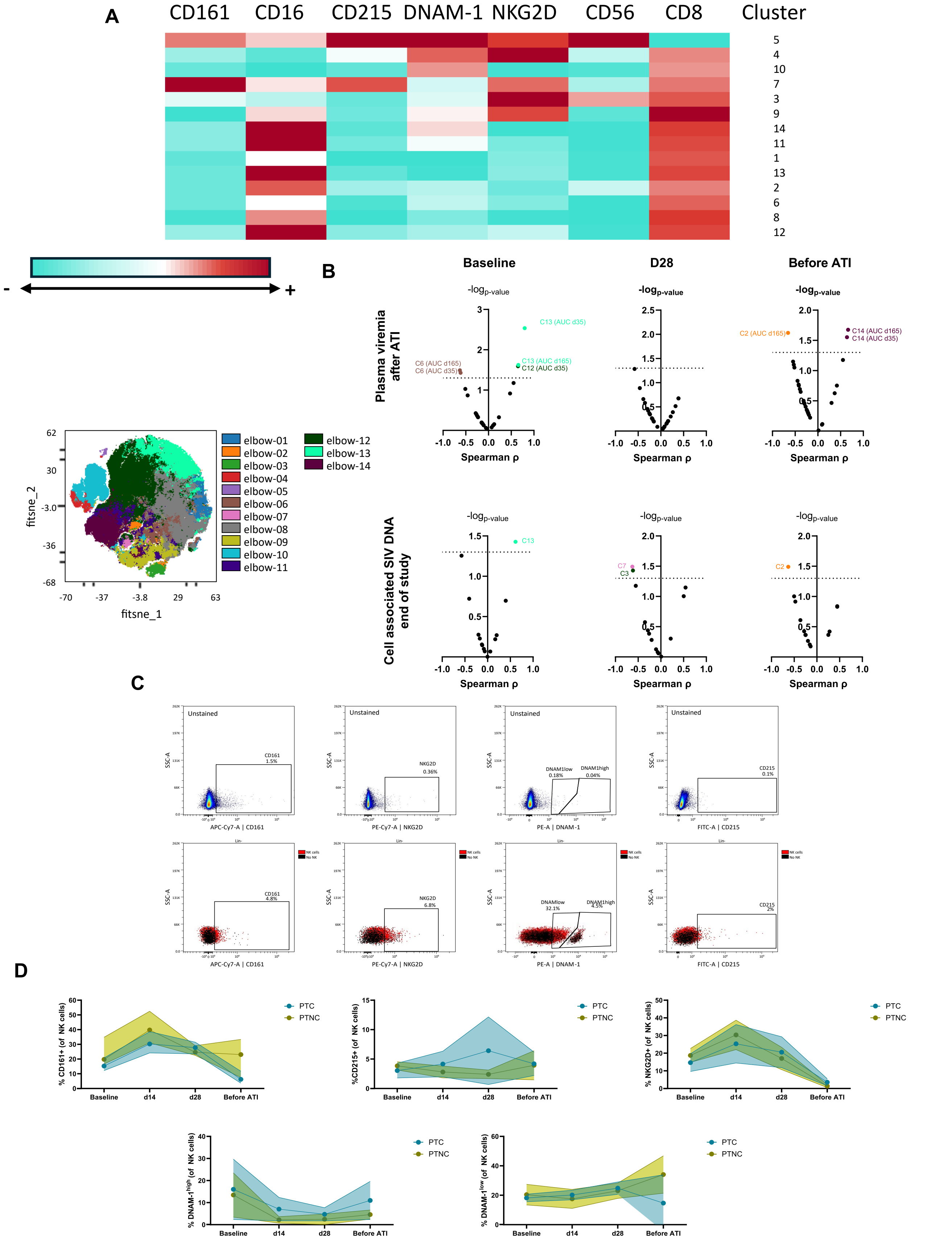

Figure S3. Longitudinal phenotyping of additional receptors on NK cells (n=12 macaques). (A) Relative expression of 7 NK cell markers in the unsupervised 14 clusters. (B) Volcano plots showing p-value and Spearman ρ of the correlation between cluster abundance and AUC of viral load during ATI (top) or total SIV DNA copies/10^6 $CD4^+$ T-cells in mesenteric lymph nodes (mLN) at euthanasia (bottom). **(C)** Manual gating of CD161, CD215, NKG2D and DNAM-1 expression on Macaques. **(D)** Frequency of $CD161^+$, $CD215^+$, $NKG2D^+$, DNAM-1^{high} and DNAM-1^{low} NK cells during acute infection and before ATI in PTC and PTNC. Dimensional reduction was performed by Fit-tSNE and clustering by FlowSOM. The darker line represents the median per group. Multiple comparison was performed by Two-way ANOVA and False Discovery Rate (FDR) correction (Benjamini, Krieger and Yekutieli). Correlations were assessed using Spearman's rank method. Significant values were considered when p-value<0.05 and q-value<0.05.

.

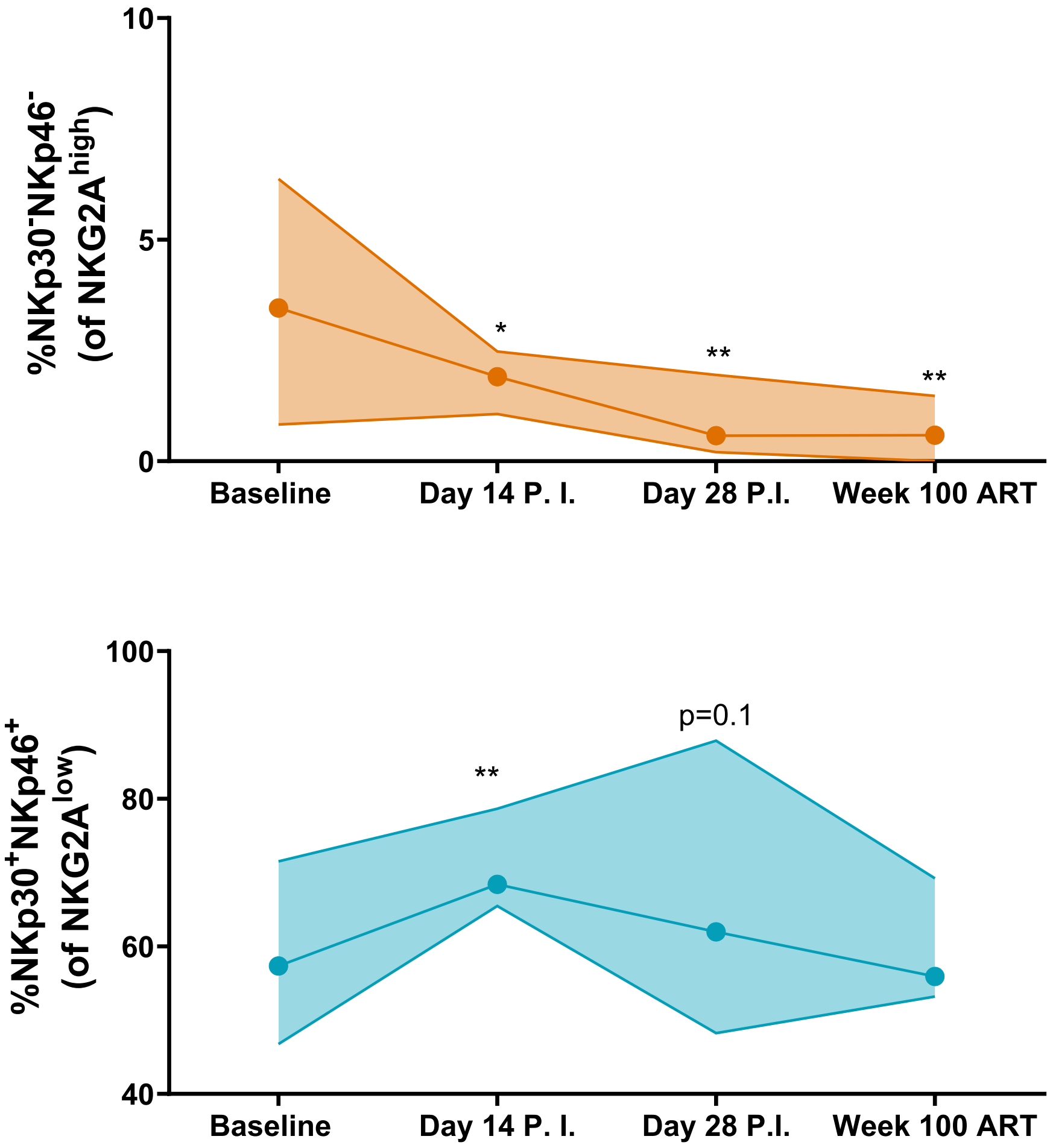

Figure S4. NKG2A^{high}NKp30^{-}NKp46^{-} and NKG2A^{low}NKp30^{+}NKp46^{+} dynamics in macaques (n=12 macaques). Frequency of NKG2A^{high}NKp30^{-}NKp46^{-} (top) or NKG2A^{low}NKp30^{+}NKp46^{+} (bottom) cells at the baseline, during acute infection and before ATI. The darker line represents the median per group. Multiple comparison was performed by Two-way ANOVA and False Discovery Rate (FDR) correction (Benjamini, Krieger and Yekutieli). Significant values were considered when p-value<0.05 and q-value<0.05.

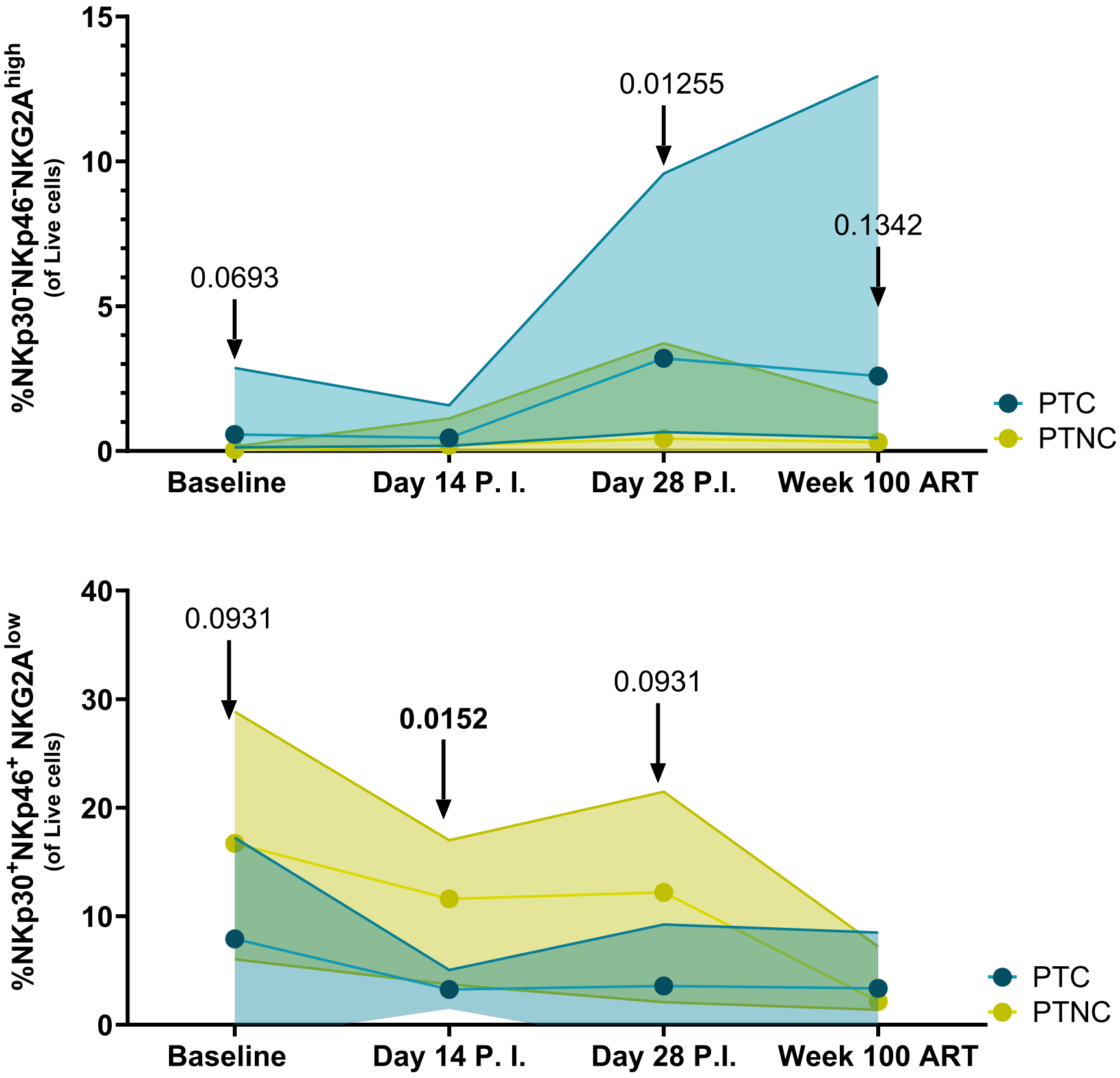

Figure S5. NKG2AhighNKp30-NKp46- and NKG2AlowNKp30+NKp46+ dynamics as % of total live cells. Frequency of NKG2AhighNKp30-NKp46- (top) or NKG2AlowNKp30+NKp46+ (bottom) cells during acute infection and before ATI in PTC and PTNC. The darker line represents the median per group (n=6 animals each, areas indicate the IQR)

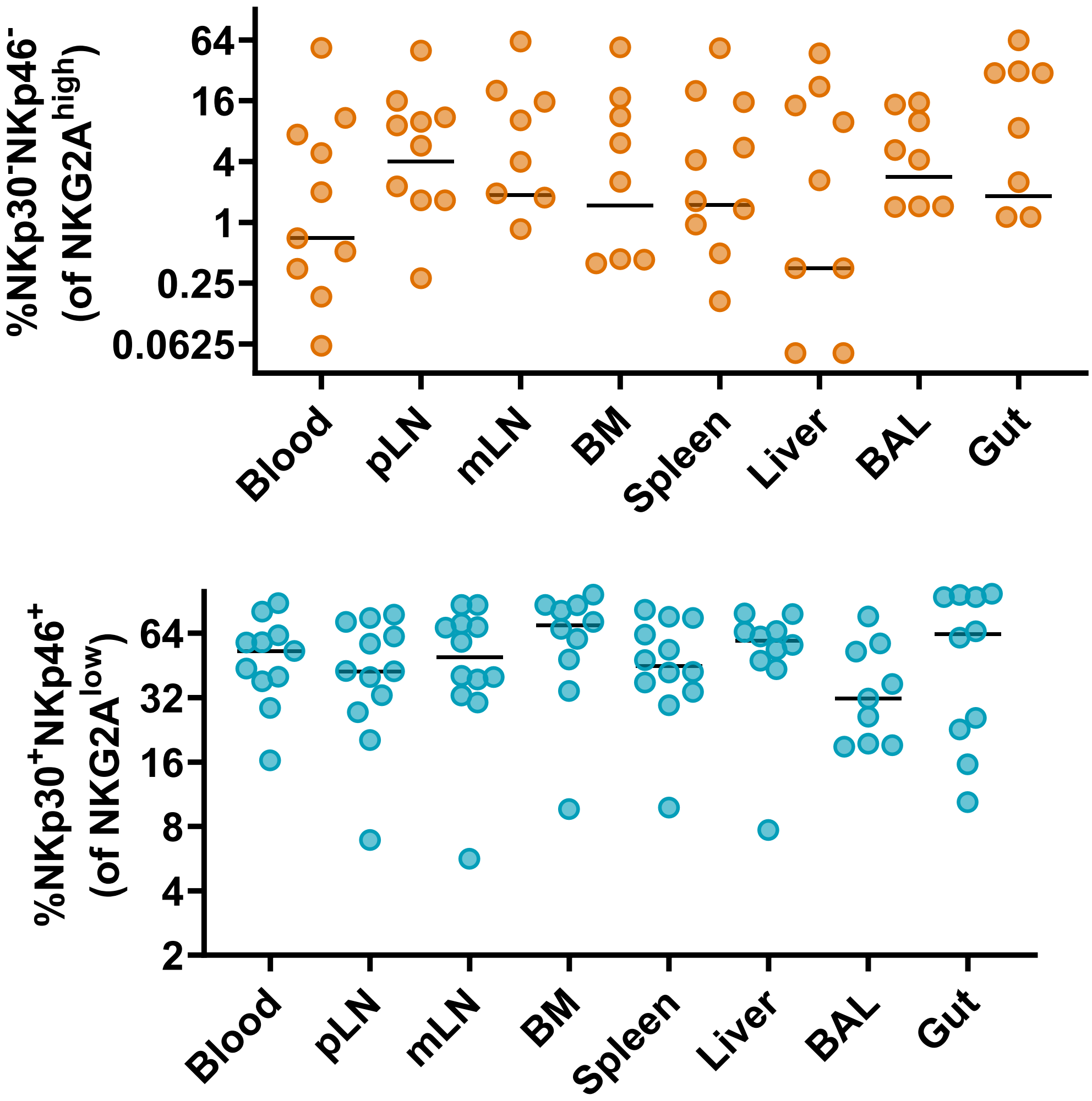

Figure S6. Abundance of NKG2A^{high}NKp30^{-}NKp46^{-} and NKG2A^{low}NKp30^{+}NKp46^{+} in different tissues at euthanasia. Each data point represents one individual macaque (n=12 independent biological replicates), horizontal lines represent the median. Multiple comparison was performed by Two-way ANOVA and False Discovery Rate (FDR) correction (Benjamini, Krieger and Yekutieli). Significant values were considered when p-value<0.05 and q-value<0.05.

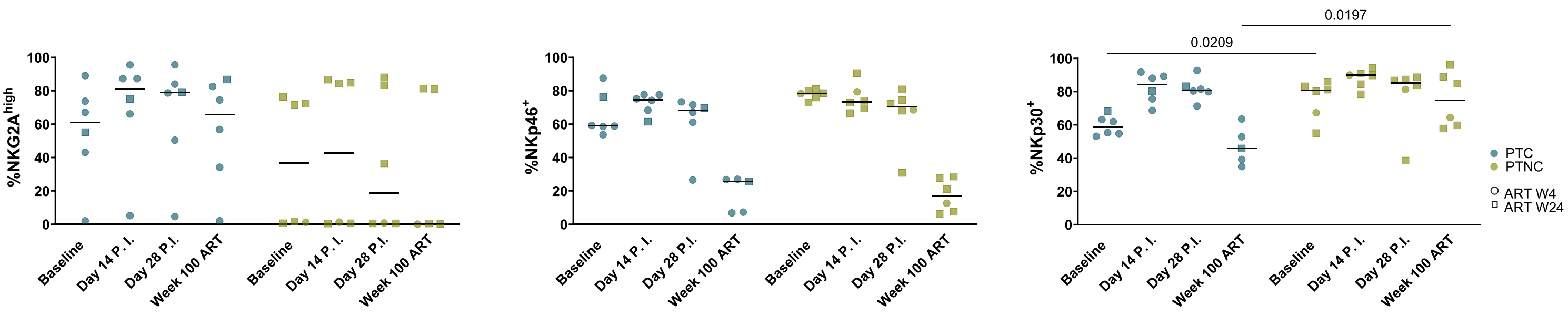

Figure S7. Frequency of NKG2A^{high} (left), NKp46^{+} (middle) and NKp30^{+} (right) cells during the study in PTC and PTNC at the baseline, during acute infection and before ATI. Each data point represents one individual macaque (n=12 independent biological replicates), horizontal lines represent the median. Multiple comparison was performed by Two-way ANOVA and False Discovery Rate (FDR) correction (Benjamini, Krieger and Yekutieli). Significant values were considered when p-value<0.05 and q-value<0.05.

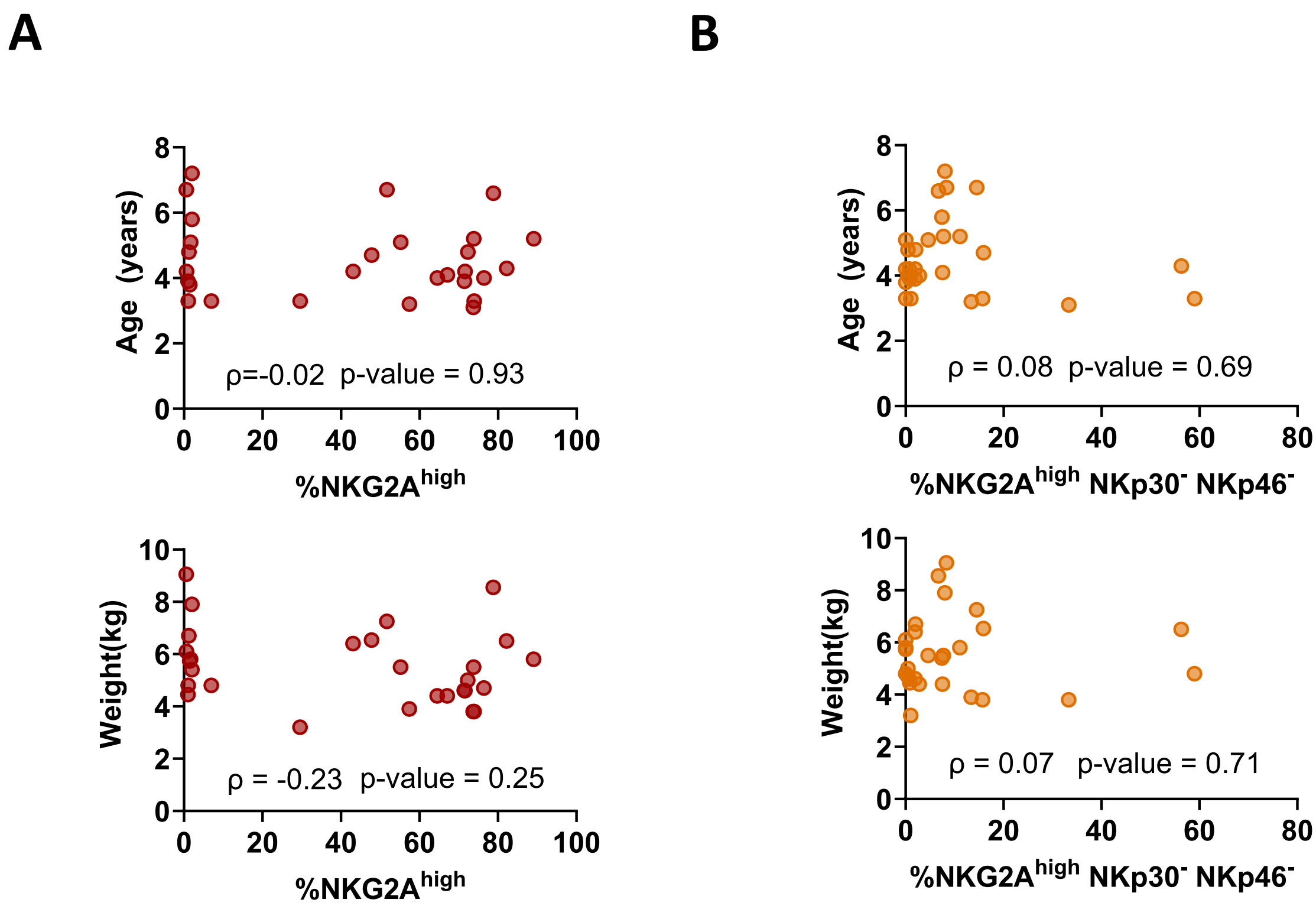

Figure S8. Correlation between the frequency of NKG2A^{high} cells (left) and NKG2A^{high}NKp30^{-}NKp46^{-} (right) at the baseline with age and weight. Each data point represents one individual macaque (n=28 independent biological replicates), Correlations were assessed using two-sided Spearman's rank method. Significant values were considered when p-value<0.05.

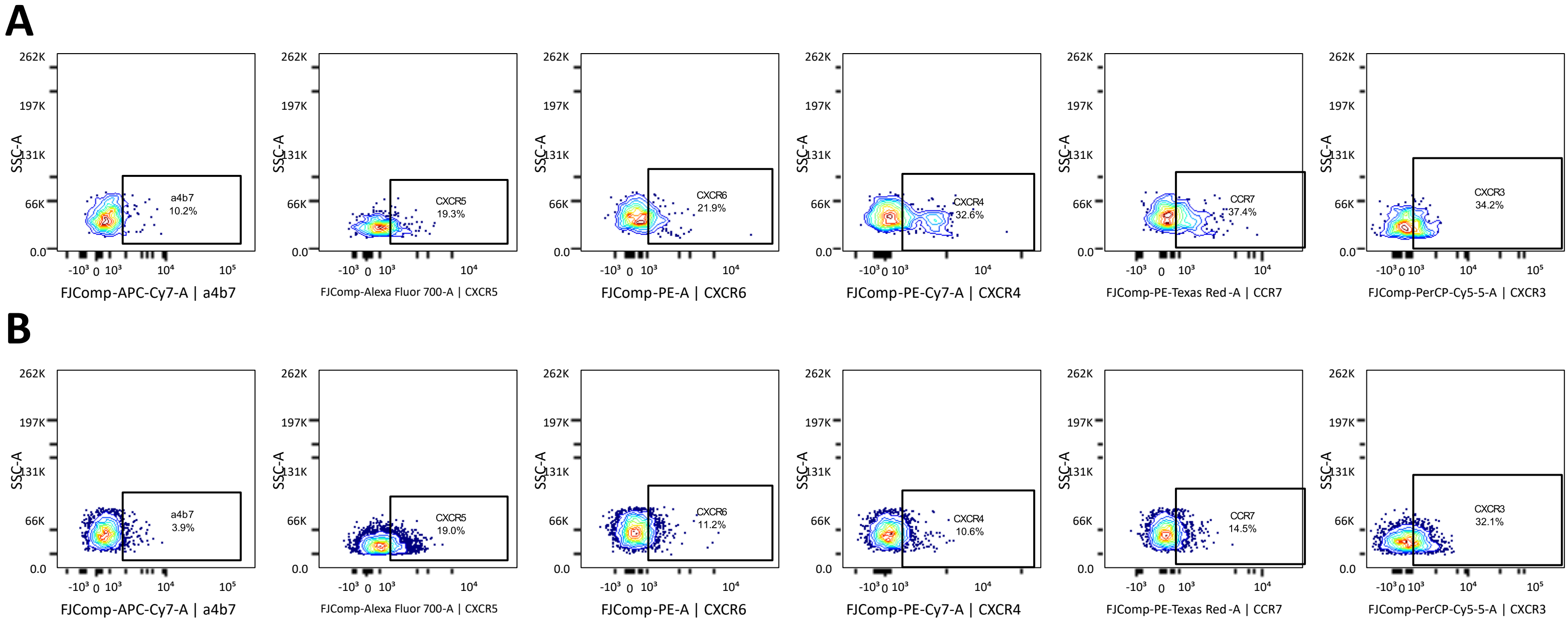

Figure S9. Gating strategy for homing markers on NKG2A^{high} cells (A) and NKG2A^{low} (B).

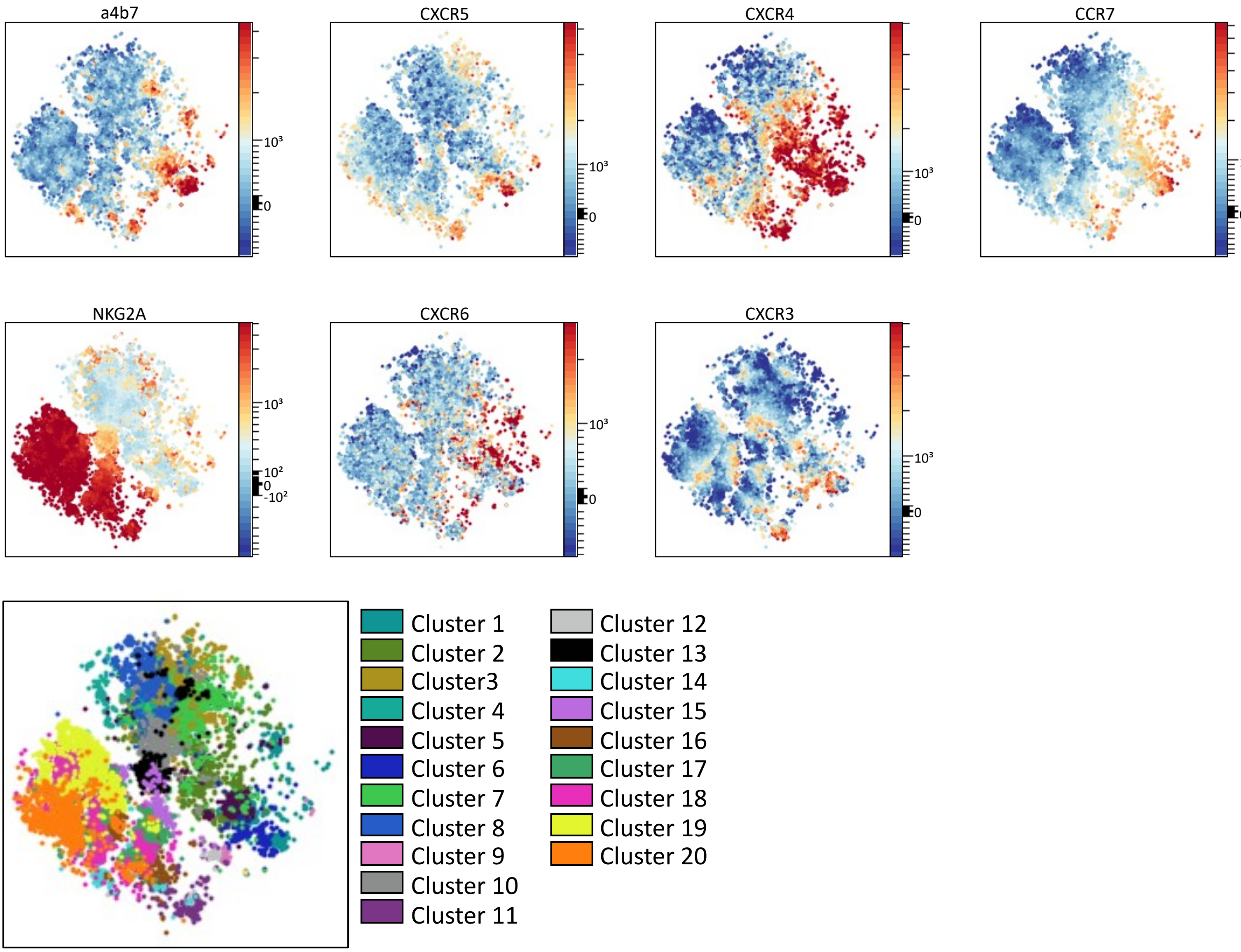

Figure S10. tSNE and expression of homing markers in NK cells before ATI.

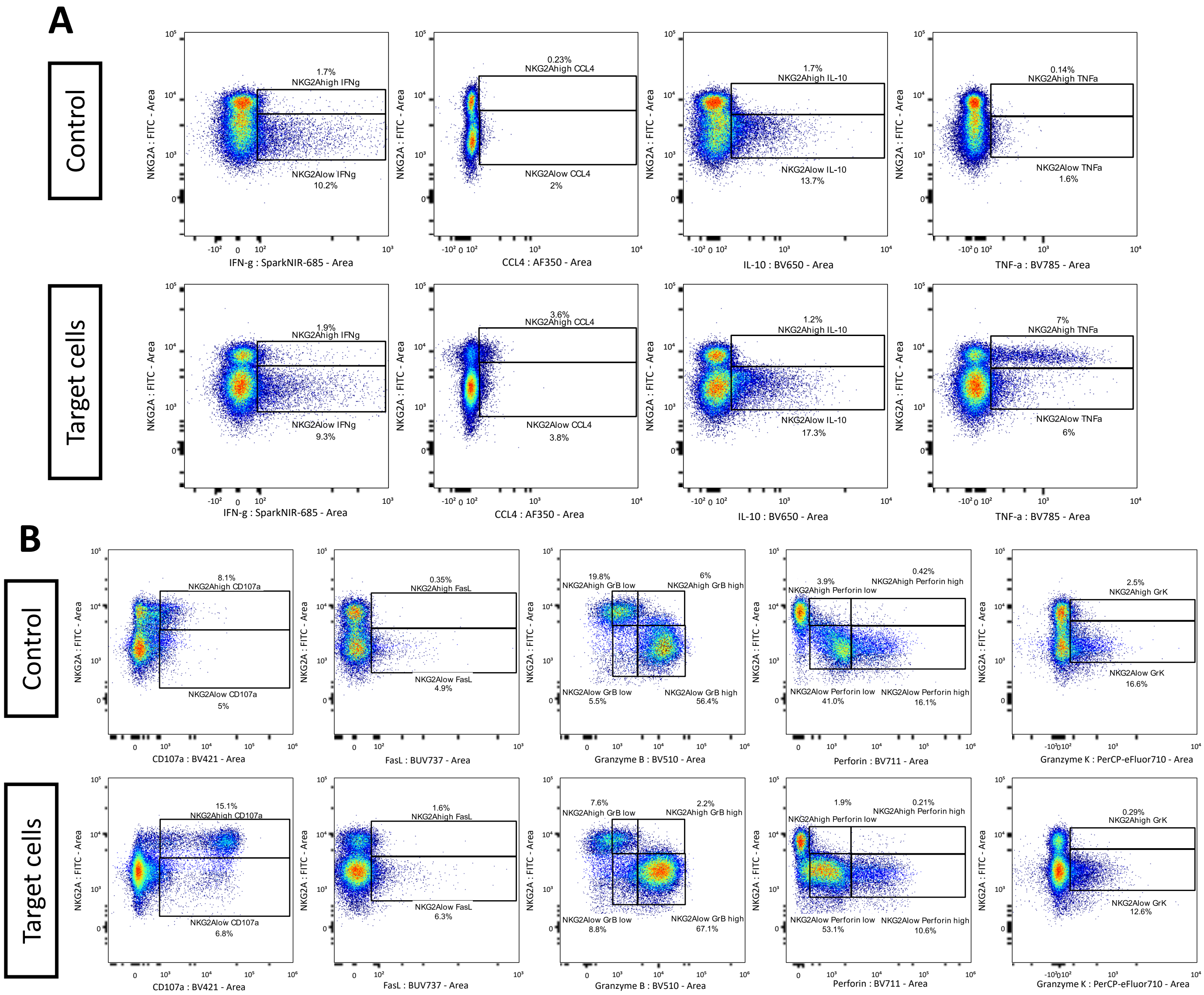

Figure S11. Gating strategy for (A) cytokine and (B) cytotoxic response to target cells regarding NKG2A expression.

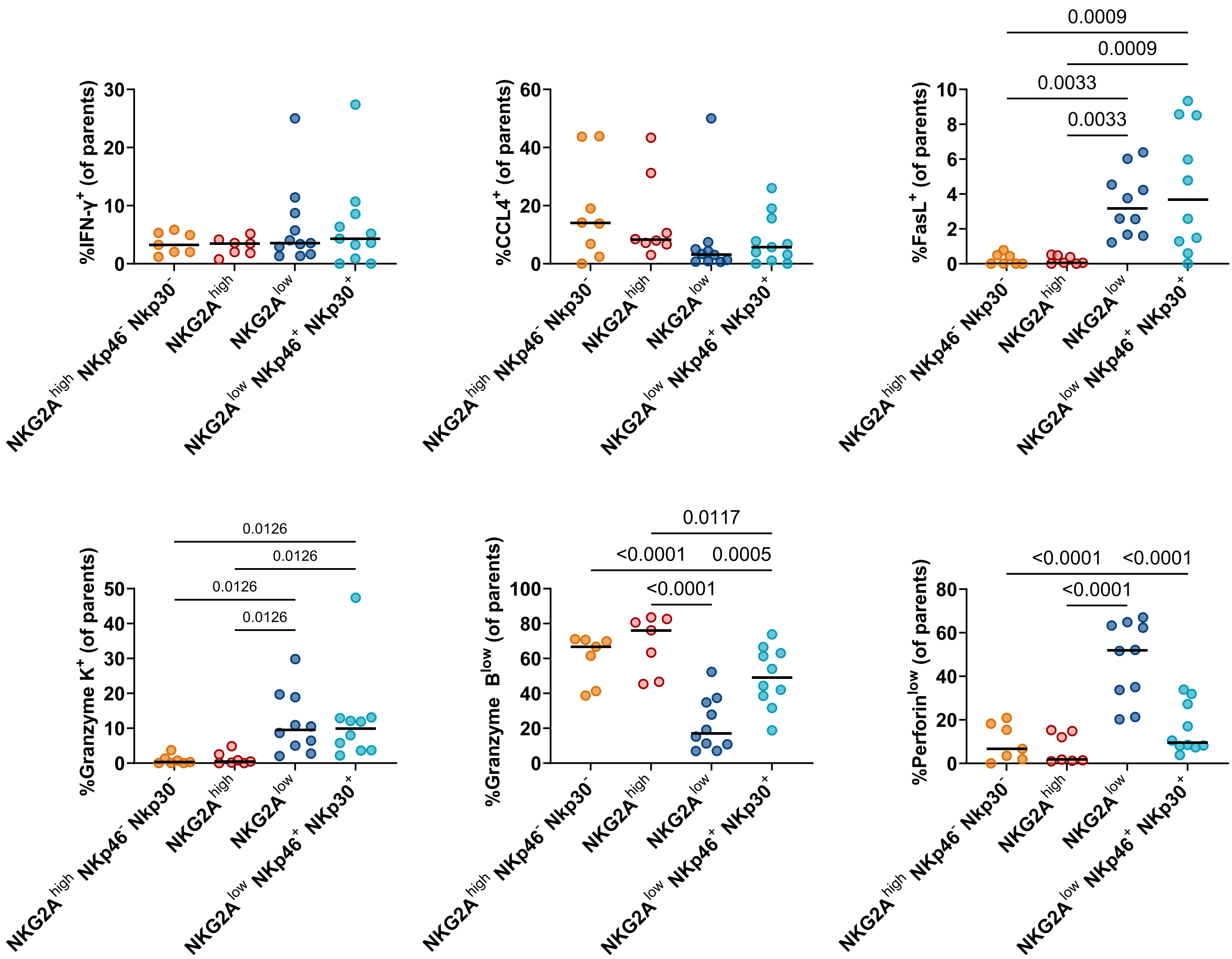

Figure S12. Comparison of IFN-γ (top, left), CCL4 (top, middle), FasL (top, right), Granzyme K (bottom, left) and Granzyme B^{low} (bottom, middle) and Perforin^{low} (bottom, right) levels in NKG2A^{high}, NKG2A^{high}NKp30^{-}NKp46^{-}, NKG2A^{low} and NKG2A^{low}NKp30^{+}NKp46^{+}. Each data point represents one individual macaque (n=12 independent biological replicates), horizontal lines represent the median. Multiple comparison was performed by Two-way ANOVA and False Discovery Rate (FDR) correction (Benjamini, Krieger and Yekutieli). Significant values were considered when p-value<0.05 and q-value<0.05.

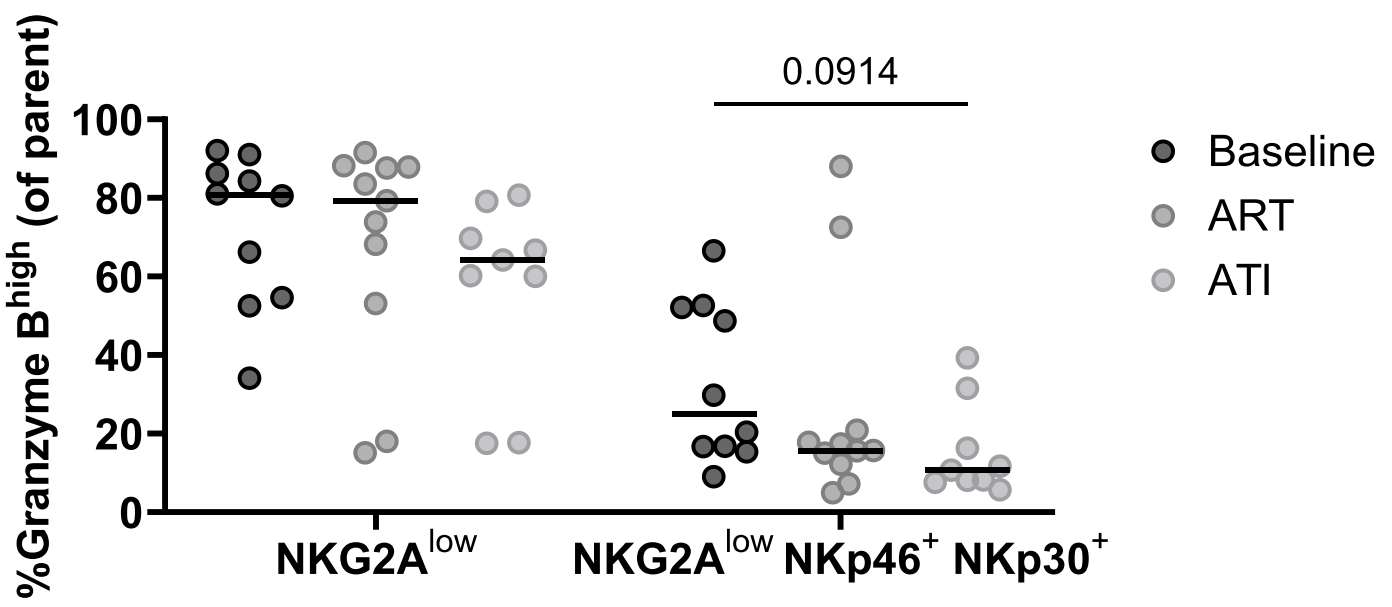

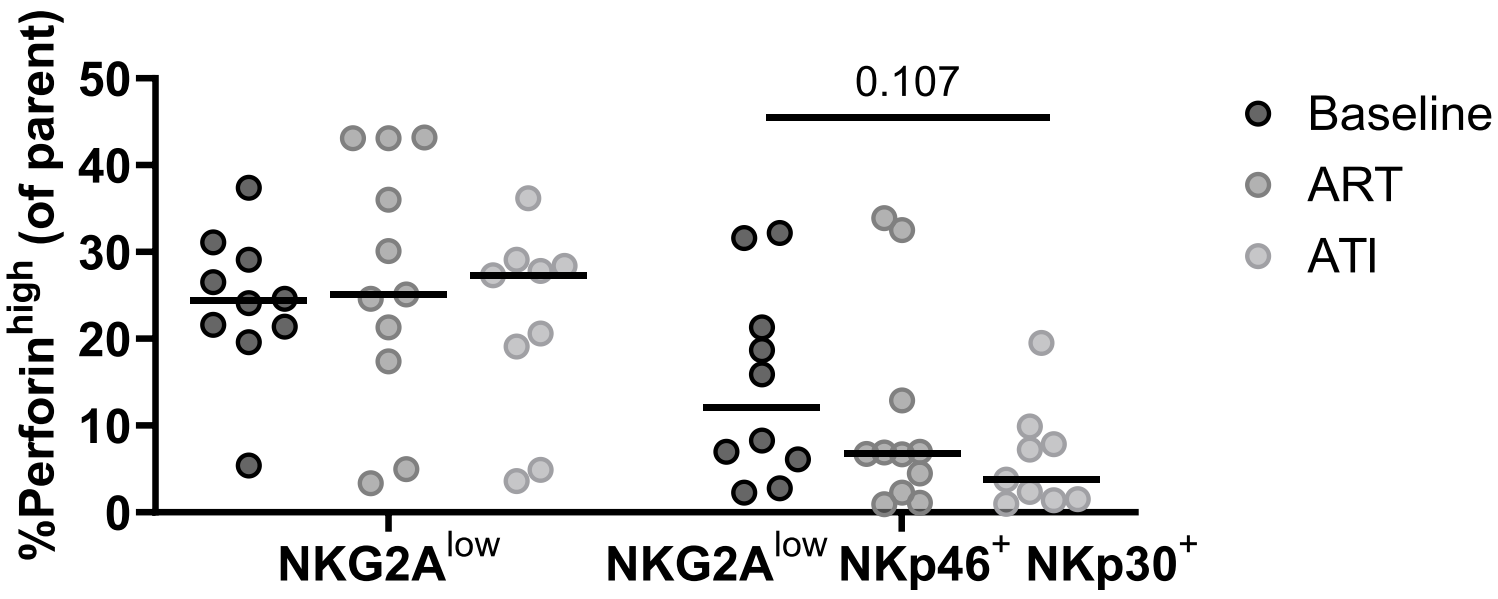

Fig. S13. Comparison of Granzyme B^{high} (left) and Perforin^{high} (right) levels in NKG2A^{low} and NKG2A^{low}NKp30^{+}NKp46^{+} cells over time. Each data point represents one individual macaque (n=12 independent biological replicates), horizontal lines represent the median. Multiple comparison was performed by Two-way ANOVA and False Discovery Rate (FDR) correction (Benjamini, Krieger and Yekutieli). Significant values were considered when p-value<0.05 and q-value<0.05.

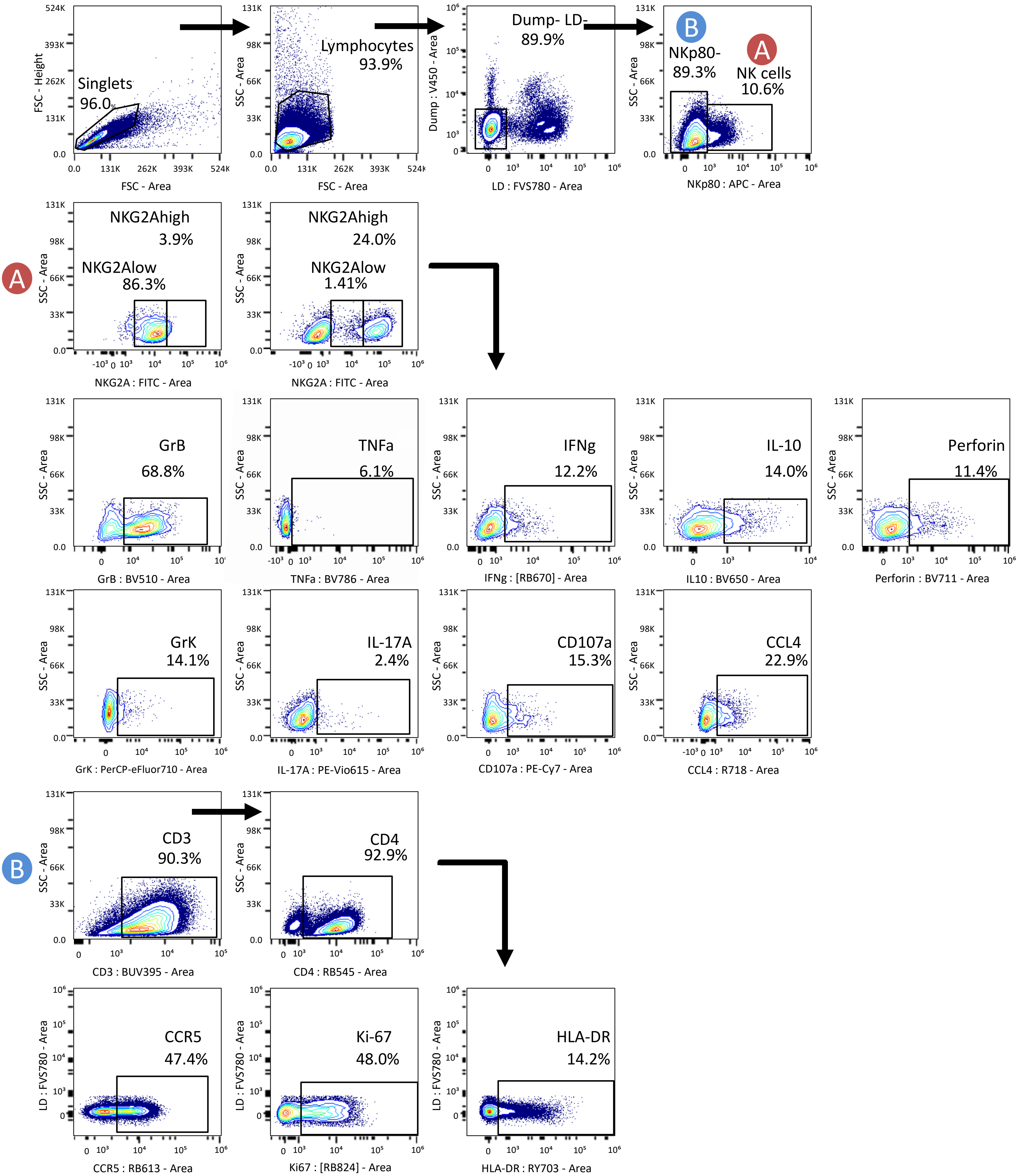

Fig. S14. Gating strategy for NK cell function (A) and CD4⁺ T-cell activation (B) in the coculture system.

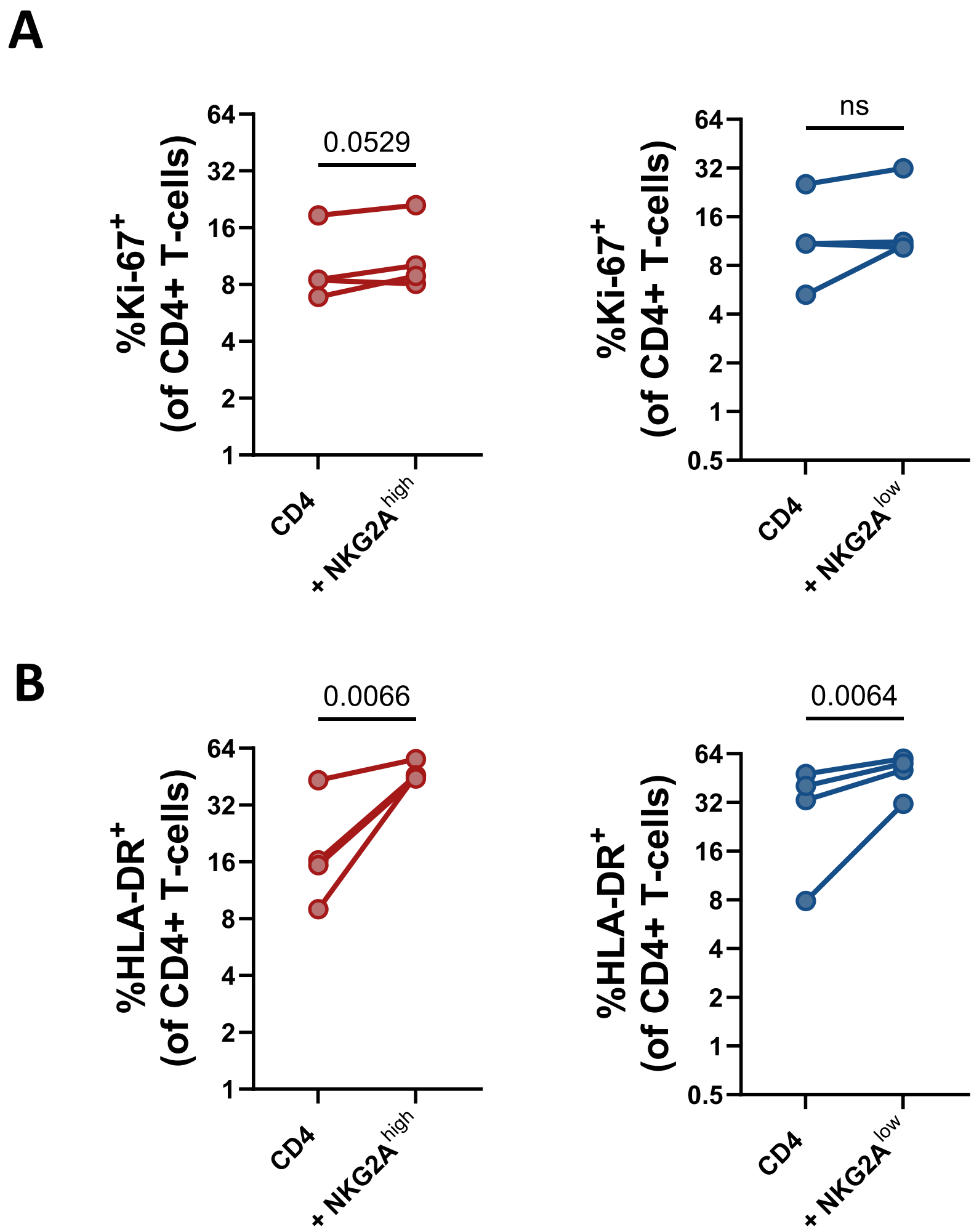

Fig. S15. Changes in activation markers on CD4+ T cells. Percentage of Ki-67+ (A) and HLA-DR+ (B) SIV-infected CD4+ T-cells upon culture alone or coculture with NKG2A^{high} NK cells (left, n=4 macaques) or NKG2A^{low} NK cells (right, n=4 macaques). Each data point represents one individual macaque. Comparisons were made by two-sided Wilcoxon signed-rank test and considered significant when p-value < 0.05.

ID	Age (years)	Weight (kg)	Haplotype	Microsatellite 277/278	Microsatellite 146	Microsatellite 213	Microsatellite 110	NKG2A	PTC
BA777K	4.8	6.7	M2/M1	+	-	-	-	low	No PTC
BA9221	5.8	5.4	M1/M3	-	-	-	-	low	PTC
BB425F	5.1	5.8	M1/M2M5	-	-	-	+	low	No PTC
CCB063	4.2	6.1	M1/M2M5	+	-	+	+	low	No PTC
CA706F	4.1	4.4	M5/M4M1M2	+	-	+	+	high	PTC
BA736J	5.2	5.5	M1M2M1/M4	-	+	-	-	high	PTC
CCB065	4.2	6.4	M3/M4	-	+	-	-	high	PTC
BB123J	5.2	5.8	M2M1/M3	-	+	-	-	high	PTC
BA912K	4.8	5	M1/M2	+	-	-	-	high	No PTC
BA987H	5.1	5.8	M4/M3	-	+	-	-	high	PTC
CCB114	4.2	4.6	M3M4/M2	-	+	-	-	high	PTC
CCE007	4	4.7	M1/M3	+	+	-	-	high	No PTC

Table S1. Characteristics of Cynomolgus macaques included in the study. PTC = Post-treatment controller, PTNC = Post-treatment non-controller.

ID	Age (years)	Weight (kg)	Haplotype	Microsatellite 277/278	Microsatellite 146	Microsatellite 213	Microsatellite 110	NKG2A
CCA035	3.3	4.8	M3/M1M4	+	+	-	-	low
CCA033	3.3	3.2	M5M1/M4M1	+	+	+	-	low
CCA100	3.3	4.8	M5/M1	+	-	-	+	low
CCC066	3.8	5.74	M1/M2	+	-	-	-	low
CCB028	3.9	4.45	M1/M1	+	-	-	-	low
BB103G	7.2	7.9	M4/M2	-	+	-	-	low
BB9I	6.7	9.05	M1/M2	+	-	-	-	low
CCA032	3.3	3.8	M3M2/M3	-	+	-	-	high
CCC039	3.1	3.8	M4/M3	-	+	-	-	high
CCB117	3.2	3.9	M1/M2	+	-	-	-	high
CCB070	3.9	4.61	M3/M3M4	-	+	-	-	high
CBK061	4	4.4	M4M1M2/M2M3	-	+	-	-	high
BA797I	6.6	8.55	M4/M2	+	+	-	-	high
BB799G	4.3	6.5	M3M5/M2M3	-	+	-	-	high
CB296A	6.7	7.25	M4/M1	+	+	-	-	high
CB806C	4.7	6.54	M1M5M3/M3	+	+	-	-	high

Table S2. Characteristics of additional Cynomolgus macaques included for MHC analyses

Table S3. Primers used to amplify the 20 MHC microsatellites

Microsatellite	Forward	Reverse
**D6S2972**	AAATGTGAGAATAAAGGAGA	GATAAAGGGGAACTACTACA
**D6S2970**	TCCCATGGTCAAGTTCTCAG	TCATGGATCTTATCAGCCTC
**D6S2854**	TCATGAGCGTGGCACTGCAC	CCGTATGTTGCAACCAGGAG
**D6S2704**	TTTTGCCACTCTGGAGGATGGG	GAGCATAATATCTGGTCTACTGC
**D6S2847**	TATTGGACAGCACTGCTCTGG	TGCCATTCAGATTGGTTTTTCTG
**C4-2-25**	ATGTTAGTTTTAGAAGATAACACTC	TCTTCTGTGCAAGCAAGCACTGTAC
**D6S2691**	GTAGCTGTGGAAACAGTGTCCATG	CTTGACTTGAAACTCAGAGACC
**B2**	GTAGGAGAATTGCTTGAACCCG	TTGCAAACATTAGGACCTTTCTTG
**P03-193435**	CAGGAGTGCTGAAGTCAT	CACTCCAGAACCTGGTGACA
**MICA**	CCTTTTTTTCAGGGAAAGTGC	CCTTACCATCTCCAGAAACTGC
**D6S2793**	CTACCTCCTTGCCAAACTTGCTATTTGT	AATAGCCATGAGAAGCTATGTGGGGGA
**D6S2782**	TTTACTTGCTCTCACTCTCAGGCC	GGAAGACATTAACTTGTTAGCA
**D6S2669**	TGCCTTCCGTAAGCCTCAGTCT	TTAAGGACAGCAAAGCCAGCAGCA
**D6S2892**	TGCATGTCCTGTGAGGTAAG	ACTCAACCCTGCTGTTGTAG
**DRACA**	TGGAATCTCATCAAGGTCAG	ACATTTGTATGCTTCAGATG
**D6S2876**	GGTAAAATTCCTGACTGGCC	GACAGCTCTTCTTAACCTGC
**D6S2747**	AGGAATCTAGTGCTCTCTCC	CTCTAGCAAAAGGAAGAGCC
**D6S2745**	CCTAGAGATTCCTCCACATTA	CCAATGTTTGATAGCAGACTGGGGT
**D6S2771**	ATTCCTTTCACTAGTTCTGG	CCACTTTAAGAAATTAGAAAAG
**D6S2741**	AGACTAGATGTAGGGCTAGC	CTGCACTTGGCTATCTCAAC
