# Supplementary material for: Constitutive natural killer cell features associated with post-treatment control of SIV infection in the pVISCONTI study
Source: Nat Commun. 2026 Aug 22;17:10059. doi: 10.1038/s41467-026-76699-7 (PMC13594178; doi:10.1038/s41467-026-76699-7)
Supplement: Supplementary file 2 [file 41467_2026_76699_MOESM2_ESM.pdf]

nature portfolio

Corresponding author(s): Asier Saez-Cirion

Last updated by author(s): Jul 15, 2026

Reporting Summary

Nature Portfolio wishes to improve the reproducibility of the work that we publish. This form provides structure for consistency and transparency in reporting. For further information on Nature Portfolio policies, see our Editorial Policies and the Editorial Policy Checklist.

Statistics

For all statistical analyses, confirm that the following items are present in the figure legend, table legend, main text, or Methods section.

n/a	Confirmed	
☐	☒	The exact sample size (*n*) for each experimental group/condition, given as a discrete number and unit of measurement
☐	☒	A statement on whether measurements were taken from distinct samples or whether the same sample was measured repeatedly
☐	☒	The statistical test(s) used AND whether they are one- or two-sided<br>*Only common tests should be described solely by name; describe more complex techniques in the Methods section.*
☐	☒	A description of all covariates tested
☐	☒	A description of any assumptions or corrections, such as tests of normality and adjustment for multiple comparisons
☐	☒	A full description of the statistical parameters including central tendency (e.g. means) or other basic estimates (e.g. regression coefficient) AND variation (e.g. standard deviation) or associated estimates of uncertainty (e.g. confidence intervals)
☐	☒	For null hypothesis testing, the test statistic (e.g. *F*, *t*, *r*) with confidence intervals, effect sizes, degrees of freedom and *P* value noted<br>*Give P values as exact values whenever suitable.*
☒	☐	For Bayesian analysis, information on the choice of priors and Markov chain Monte Carlo settings
☒	☐	For hierarchical and complex designs, identification of the appropriate level for tests and full reporting of outcomes
☐	☒	Estimates of effect sizes (e.g. Cohen's *d*, Pearson's *r*), indicating how they were calculated

Our web collection on statistics for biologists contains articles on many of the points above.

Software and code

Policy information about availability of computer code

Data collection	Flow cytometry data was collected with Diva (v6.1.2) and the SonyID7000 software (v2.2.1).
Data analysis	Graph and statistical analysis were performed using Prism version 11.0.2 (GraphPad Software) or R (version 4.5.2). High-dimensional data was analyzed with Qlucore software (version 3.10). For flow cytometry, data dimensionality was performed using fit-tSNE (OMIQ). Data normalization was applied by Cycombine. Unsupervised clustering was performed using FlowSOM. The optimal number of clusters was determined by the elbow method.

For manuscripts utilizing custom algorithms or software that are central to the research but not yet described in published literature, software must be made available to editors and reviewers. We strongly encourage code deposition in a community repository (e.g. GitHub). See the Nature Portfolio guidelines for submitting code & software for further information.

Data

Policy information about availability of data

All manuscripts must include a data availability statement. This statement should provide the following information, where applicable:
- Accession codes, unique identifiers, or web links for publicly available datasets
- A description of any restrictions on data availability
- For clinical datasets or third party data, please ensure that the statement adheres to our policy

All data generated in this study are provided in the Source Data file. The Source Data file contains the individual numerical values underlying all main and

supplementary figures, including plasma viral loads, cell-associated SIV-DNA, NK cell phenotypic and functional measurements, homing marker analyses, MHC microsatellite genotyping/haplotype assignments, and coculture assay results.

Research involving human participants, their data, or biological material

Policy information about studies with human participants or human data. See also policy information about sex, gender (identity/presentation), and sexual orientation and race, ethnicity and racism.

Reporting on sex and gender	NA
Reporting on race, ethnicity, or other socially relevant groupings	NA
Population characteristics	NA
Recruitment	NA
Ethics oversight	NA

Note that full information on the approval of the study protocol must also be provided in the manuscript.

Field-specific reporting

Please select the one below that is the best fit for your research. If you are not sure, read the appropriate sections before making your selection.

☒ Life sciences ☐ Behavioural & social sciences ☐ Ecological, evolutionary & environmental sciences

For a reference copy of the document with all sections, see nature.com/documents/nr-reporting-summary-flat.pdf

Life sciences study design

All studies must disclose on these points even when the disclosure is negative.

Sample size	The sample size determined in the pVISCONTI study followed ethical recommendations and good practices for the use of non-human primates in biomedical research. Sample size were defined in order to assume a statistical analyses with a level of significance of 5% (α = 0.05), a target power of 80% (1 - β = 0.8) and an effect size of 1.6 with the objective to use non-parametric tests (Kruskal-Wallis, Wilcoxon rank and Mann & Whitney tests) for group comparisons, with Dunn-Bonferroni correction) to adjust p-values when multiple comparisons are performed. 12 animals (6 treated early, W4 p.i. and 6 treated late W24p.i.) were dedicated to additional sampling for immunological studies and were analyzed in this substudy.
Data exclusions	Analyses were conducted in all available samples from the animal in the study at the timepoints indicated. No data was excluded.
Replication	The pVISCONTI study was performed in two independent experimental phases, each one including 6 W4- and 6 W24-treated macaques that were infected, treated, and monitored in parallel with reproducible results. This substudy includes all the animals from the first phase of the study.
Randomization	Animals were allocated to each group to closely match in terms of age, weight, and genotype.<br>Sample collection and analyses were performed in random order
Blinding	The investigators were not blinded as per study design: sampling calendar was adapted to the delay to treatment initiation/treatment interruption for the different groups

Reporting for specific materials, systems and methods

We require information from authors about some types of materials, experimental systems and methods used in many studies. Here, indicate whether each material, system or method listed is relevant to your study. If you are not sure if a list item applies to your research, read the appropriate section before selecting a response.

Materials & experimental systems

n/a	Involved in the study
☐	☒ Antibodies
☐	☒ Eukaryotic cell lines
☒	☐ Palaeontology and archaeology
☐	☒ Animals and other organisms
☒	☐ Clinical data
☒	☐ Dual use research of concern
☒	☐ Plants

Methods

n/a	Involved in the study
☒	☐ ChIP-seq
☐	☒ Flow cytometry
☒	☐ MRI-based neuroimaging

Antibodies

Antibodies used

TThe following antibodies were used:

anti-CD3–V450 (clone SP34-2, BD Biosciences), Cat# 560351, Cross-reactivity: Technical Datasheet, Dilution: 1/30; anti-CD14–V450 (clone M5E2, BD Biosciences), Cat# 561390, Cross-reactivity: NHP Reagents Database, Dilution: 1/300; anti-CD20–V450 (clone L27, BD Biosciences), Cat# 642283, Cross-reactivity: NHP Reagents Database, Dilution: 1/40; anti-NKG2A–FITC (clone REA110, Miltenyi), Cat# 130-115-978, Cross-reactivity: Technical Datasheet and Laboratory control (FMO+Positive control), Dilution: 1/50; anti-NKp46–PC7 (clone BAB281, Beckman Coulter), Cat# B38703, Cross-reactivity: Technical Datasheet, Dilution: 1/6; anti-CD8–PerCP-Vio700 (clone BW135/80, Miltenyi), Cat# 130-113-161, Cross-reactivity: Technical Datasheet, Dilution: 1/100; anti-CD56–PE-CF594 (clone NCAM16.2, BD Biosciences), Cat# 564849, Cross-reactivity: NHP Reagents Database, Dilution: 1/100; anti-NKp30–PE (clone AF29-4D12, Miltenyi), Cat# 130-092-483, Cross-reactivity: Technical Datasheet, Dilution: 1/30; anti-CD69–APC-Cy7 (clone FN50, BioLegend), Cat# 310914, Cross-reactivity: NHP Reagents Database, Dilution: 1/30; anti-CD16–AF700 (clone 3G80, BioLegend), Cat# 302026, Cross-reactivity: NHP Reagents Database, Dilution: 1/20; anti-NKp80–APC (clone 4A4.D10, Miltenyi), Cat# 130-128-949, Cross-reactivity: Technical Datasheet, Dilution: 1/10; anti-CD215–FITC (clone eBioJM7A4, Thermofisher), Cat# 11-7159-42, Cross-reactivity: Technical Datasheet, Dilution: 1/20; anti-NKG2D–PE-Vio770 (clone BAT221, Miltenyi), Cat# 130-125-098, Cross-reactivity: Technical Datasheet, Dilution: 1/10; anti-DNAM-1–PE (clone DX11, Miltenyi), Cat# 130-124-232, Cross-reactivity: Technical Datasheet, Dilution: 1/30; anti-CD161–APC-Cy7 (clone HP3G10, BioLegend), Cat# 339904, Cross-reactivity: Technical Datasheet, Dilution: 1/20; anti-CXCR4–PE-Cy7 (clone 12G5, BD Biosciences), Cat# 560669, Cross-reactivity: Technical Datasheet, Dilution: 1/20; anti-CXCR3–PerCP-Cy5.5 (clone 1C6, BD Biosciences), Cat# 560832, Cross-reactivity: Technical Datasheet, Dilution: 1/20; anti-CCR7–PE-Dazzle594 (clone G043H7, BioLegend), Cat# 353236, Cross-reactivity: Technical Datasheet, Dilution: 1/10; anti-CXCR6–PE (clone 568, R&D), Cat# FAB699P-100, Cross-reactivity: Technical Datasheet, Dilution: 1/10; anti-α4/β7–APC-Fire750 (clone FIB504, BioLegend), Cat# 321224, Cross-reactivity: Technical Datasheet, Dilution: 1/20; anti-CXCR5–APC-R700 (clone RF8B2, BD Biosciences), Cat# 565191, Cross-reactivity: Laboratory control (FMO+Positive control), Dilution: 1/20; anti-CD107a–BV421 (clone H4A3, BioLegend), Cat# 328626, Cross-reactivity: NHP Reagents Database, Dilution: 1/100; anti-FasL–BUV737 (clone NOK-1, BD Biosciences), Cat# 748972, Cross-reactivity: Laboratory control (FMO+Positive control), Dilution: 1/20; anti-CCL4–AF350 (clone 24006, R&D), Cat# AF-271-NAU-100UG, Cross-reactivity: NHP Reagents Database, Dilution: 1/40; anti-TNF-α–BV785 (clone MAb11, BioLegend), Cat# 502948, Cross-reactivity: Technical Datasheet, Dilution: 1/20; anti-IL-10–BV650 (clone JES3-9D7, BD Biosciences), Cat# 564051, Cross-reactivity: NHP Reagents Database, Dilution: 1/20; anti-Granzyme-B–BV510 (clone GB11, BioLegend), Cat# 396432, Cross-reactivity: Technical Datasheet, Dilution: 1/40; anti-Granzyme K–PerCP-eFluor710 (clone G3H69, Thermofisher), Cat# 46-8897-42, Cross-reactivity: Laboratory control (FMO+Positive control), Dilution: 1/100; anti-Perforin–BV711 (clone dG9, BioLegend), Cat# 308130, Cross-reactivity: Laboratory control (FMO+Positive control), Dilution: 1/40; anti-IL-17A–PE-Vio615 (clone CZ8-23G1, Miltenyi), Cat# 130-120-412, Cross-reactivity: Technical Datasheet, Dilution: 1/20; anti-IFN-γ–Spark NIR 685 (clone 4S.B3, BioLegend), Cat# 502552, Cross-reactivity: NHP Reagents Database, Dilution: 1/40; anti-CD107a–PE-Cy7 (clone H4A3, BD Biosciences), Cat# 561348, Cross-reactivity: NHP Reagents Database, Dilution: 1/20; anti-CD3–BUV395 (clone SP34-2, BD Biosciences), Cat# 564117, Cross-reactivity: Technical Datasheet, Dilution: 1/40; anti-CD4–RB545 (clone L200, BD Biosciences), Cat# 756250, Cross-reactivity: Technical Datasheet, Dilution: 1/20; anti-CCR5–RB613 (clone 3A9, BD Biosciences), Cat# 758898, Cross-reactivity: NHP Reagents Database, Dilution: 1/20; anti-HLA-DR–RY703 (clone L203.rMAb, BD Biosciences), Cat# 770034, Cross-reactivity: Technical Datasheet, Dilution: 1/100; anti-CCL4–R718 (clone D21-1351, BD Biosciences), Cat# 567249, Cross-reactivity: Laboratory control (FMO+Positive control), Dilution: 1/20; anti-IFN-γ–RB670 (clone B27, BD Biosciences), Cat# 571942, Cross-reactivity: NHP Reagents Database, Dilution: 1/40.

Validation

Manufacturer's Specifications: We reviewed the manufacturer's specifications and technical data sheets for each antibody. This information included details such as clone names, isotypes and references. Antibody dilution was determined by titration using the same number of target cells and decreasing dilutions. Optimal titer was determined by calculating the Stain Index (SI) for each antibody.
Positive and Negative Controls: To validate antibody performance, we used positive and negative controls. Positive controls included cells known to express the target antigen, while negative controls were cells lacking the antigen.
Fluorescence Minus One (FMO) Controls: FMO controls were utilized to assess background fluorescence.
Non-Human Primate (NHP) Reagent Controls: NHP reagent controls were used to ensure the compatibility and specificity of the antibodies in the primate model.

Eukaryotic cell lines

Policy information about cell lines and Sex and Gender in Research

Cell line source(s)

K562 cell line (human, female) was obtained from the ATCC

Authentication	The K562 cell line was not authenticated.
Mycoplasma contamination	The K562 cell line was tested negative for mycoplasma
Commonly misidentified lines (See ICLAC register)	ATCC Number: CCL-243; no commonly misidentified cell lines were used in the study.

Animals and other research organisms

Policy information about studies involving animals; ARRIVE guidelines recommended for reporting animal research, and Sex and Gender in Research

Laboratory animals	Cynomolgus macaques (Macaca fascicularis), imported from Mauritius (median age = 4.8 years at inclusion, IQR = 3.9-7.2).
Wild animals	No wild animals were used in the study
Reporting on sex	In the present study only male macaques were used.
Field-collected samples	No field collected samples were used in the study
Ethics oversight	CyMs were imported from Mauritius and housed in facilities at the Infectious Disease Models and Innovative Therapies (IDMIT) center (CEA site at Fontenay-aux-Roses, France). All non-human primate studies at IDMIT are conducted in accordance with French National Regulations under the supervision of National Veterinary Inspectors (CEA Permit Number D92-032-02). IDMIT complies with the Standards for Human Care and Use of Laboratory Animals of the Office for Laboratory Animal Welfare under Assurance Number #A5826-01 and F20-00448. All experimental procedures were conducted according to European Directive 2010/63 (Recommendation Number 9). The pVISCONTI study was approved and accredited under the statement A15 035 from the "Comité d'Ethique en Expérimentation Animale du CEA", registered and authorized under Number 2453-2015102713323361v3 by the French Ministry of Education and Research. CyMs were studied with veterinary guidance, housed in adjoining individual cages allowing social interactions, and maintained under controlled conditions with respect to humidity, temperature, and light. Water was available ad libitum. Animals were monitored and fed with commercial monkey chow, vegetables and fruits once or twice daily by trained personnel. Environmental enrichment was provided in the form of toys, novel foodstuffs, and music under the supervision of IDMIT Animal Welfare Body. Experimental procedures (animal handling, viral inoculations, and samplings) were conducted after sedation with ketamine chlorhydrate (Imalgene 1000®, 10 mg/kg, intravenously (i.v.), Merial). Tissues were collected during follow up and at necropsy. Animals were euthanized after ketamine chlorhydrate sedation followed by a bolus of sodium pentobarbital (Doléthal, 180 mg/kg, i.v., Laboratoire Vetoquinol).

Note that full information on the approval of the study protocol must also be provided in the manuscript.

Plants

Seed stocks	NA
Novel plant genotypes	NA
Authentication	NS

Flow Cytometry

Plots

Confirm that:

☒ The axis labels state the marker and fluorochrome used (e.g. CD4-FITC).

☒ The axis scales are clearly visible. Include numbers along axes only for bottom left plot of group (a 'group' is an analysis of identical markers).

☒ All plots are contour plots with outliers or pseudocolor plots.

☒ A numerical value for number of cells or percentage (with statistics) is provided.

Methodology

Sample preparation	PBMCs and tissue samples were analyzed by Flow Cytometry in the present study. A detailed protocol for sample processing is indicated in the methods section, as follows:

Blood collection and processing
Peripheral blood was collected by venous puncture into Vacutainer Plus Plastic K3EDTA Tubes or Vacutainer CPT Mononuclear Cell Preparation Tubes with Sodium Heparin (BD Biosciences). Complete blood counts were monitored at all time points from the Vacutainer Plus Plastic K3EDTA Tubes. Plasma was isolated either from Vacutainer Plus Plastic K3EDTA Tubes by centrifugation for 10 min at 1,500 rpm, or from Vacutainer CPT Mononuclear Cell Preparation Tubes after centrifugation for 40 min at 3,000 rpm, and stored at −80°C. Peripheral blood mononuclear cells (PBMCs) were isolated from Vacutainer CPT Mononuclear Cell Preparation Tubes with Sodium Heparin according to manufacturer's instructions (BD Biosciences), and red blood cells were lysed in Ammonium-Chloride-Potassium (ACK) buffer (0.15 M NH4Cl, 10 mM KHCO3, 0.1 mM EDTA, pH 7.4).

Tissue collection and processing
Axillary or inguinal lymph nodes (peripheral PLNs), bone marrow samples (BM), and broncho-alveolar lavages (BAL) were collected longitudinally (Figure S1). In addition, spleen, mesenteric lymph nodes (MLNs), colon and liver were collected at necropsy. Tissue samples were collected in RPMI medium at 2–8°C. Lymph node cells were isolated into RPMI medium via mechanical disruption using a gentleMACS Dissociator (Miltenyi Biotec). The cell suspension was filtered (70 µm), and red blood cells were lysed in ACK. Bone marrow cells were purified using Lymphocyte Separation Medium (Eurobio Scientific) diluted to 90% in DPBS, centrifuged for 20 min at 350 g, and separated from red cells in ACK. Spleen cells were processed via mechanical disruption in RPMI medium using a gentleMACS Dissociator (Miltenyi Biotec), purified as described for BM cells, and separated from red cells in ACK. Colonic lymphocytes were obtained from mucosa taken from approximately 10 cm of tissue. Colonic tissue was washed extensively in PBS and R10 medium (RPMI medium supplemented with 10% fetal calf serum and penicillin/streptomycin), and then digested for 45 min with collagenase II prior to mechanical disruption. Lymphocytes were isolated over a Percoll 67/44 gradient (Sigma-Aldrich). Liver tissue was mechanically disrupted using the gentleMACS Dissociator (Miltenyi Biotec) and cell suspension was sequentially filtered (300µm > 100µm > 70µm), then lymphocytes were obtained over an OptiPrep gradient (Sigma-Aldrich). T cell activation, proliferation and exhaustion phenotyping and the measurements of SIV-suppressive activity ex vivo were performed using freshly isolated cells. T cell CFSE proliferation assay and cytokine intracellular staining were performed using viable cells frozen at −196°C in DMSO/FCS.

Instrument

Data were acquired using either a LSRII flow cytometer (BD Biosciences) or a SONY ID700 analyzer (Sony).

Software

Data was analyzed with OMIQ.

Cell population abundance

NA

Gating strategy

The general gating strategy used in the analyses explored in the current study was as follows:
Singlets (FSC-A/FSC-H)>Morphology (FSC-A/SSC-A)> Viable cells and Dump- (CD3-CD14-CD20-)> NK cells (NKp80+)> NKG2Ahigh or NKG2Alow > Dual expression of NKp30 and NKp46
Expression of homing receptors on on NKG2Ahigh, NKG2AhighNKp30-Nkp46-, NKG2Alow and NKG2AlowNKp30+NKp46+ was determined by gating on: a4B7, CXCR5, CXCR6, CXCR4, CCR7, CXCR3
NK cell function was determined on NKG2Ahigh, NKG2AhighNKp30-Nkp46-, NKG2Alow and NKG2AlowNKp30+NKp46+ by gating on: Granzyme B, Granzyme K, Perforin, CD107a, IL-17A, IL-10, IFN-g, TNF-a, CCL4
In the suppressive capacity analysis, CD4+ T cells were gated as follows;
Singlets (FSC-A/FSC-H)>Morphology (FSC-A/SSC-A)> Viable cells and Dump- (CD14-CD20-)>CD3+>CD4+
We analyzed the expression of CCR5, KI-67 and HLA-DR on CD4+ T-cells

☒ Tick this box to confirm that a figure exemplifying the gating strategy is provided in the Supplementary Information.
