# Supplementary material for: Constitutive natural killer cell features associated with post-treatment control of SIV infection in the pVISCONTI study
Source: Nat Commun. 2026 Aug 22;17:10059. doi: 10.1038/s41467-026-76699-7 (PMC13594178; doi:10.1038/s41467-026-76699-7)
Supplement: Supplementary file 3 [file 41467_2026_76699_MOESM3_ESM.pdf]

nature portfolio

Peer Review File

Constitutive Natural Killer cell features associated with post-treatment control of SIV infection in the pVISCONTI study

Corresponding Author: Dr Asier Saez-Cirion

This file contains all reviewer reports in order by version, followed by all author rebuttals in order by version.

Version 0:

Reviewer comments:

Reviewer #1

(Remarks to the Author)
The paper by Chapel et al. investigates an important issue regarding NK cell features and their influence on the treatment of retroviral infection using an animal model. The authors show that the NK cell phenotype observed in SIV-infected monkeys prior to the initiation of ART therapy may influence the course and outcomes of treatment. However, the study is very limited in terms of the range of NK cell receptors analyzed. Although the figures appear visually impressive, the study lacks functional NK cell assays and is largely limited to PBMC/NK cell phenotyping and correlations with viral load. Therefore, we believe that, in its current form, the manuscript does not meet the standards expected for publication in a high-impact journal and would require substantial improvements, if possible.

Major comments:
1. What is the rationale for selecting the specific set of NK cell receptors analyzed in this study, namely the natural cytotoxicity receptors NKp30 and NKp46 and a single inhibitory receptor, NKG2A? This represents a very limited panel of NK cell receptors. On the one hand, several key activating receptors are missing, such as NKG2D, DNAM-1, and 2B4, which have been shown to play important roles in viral infection and immune surveillance. In addition, other relevant inhibitory receptors, including TIGIT, Tim-3, CD161, and PD-1, were not included in the analysis (DOI:10.1093/cei/uxae045; DOI:10.1016/j.jhep.2010.11.030). Recent studies have also shown that DNAM-1–expressing NK cells are upregulated in HIV controllers (DOI:10.1016/j.ebiom.2025.105948), highlighting the potential importance of this receptor in the context of HIV infection. On the other hand, the inhibitory receptor NKG2A recognizes HLA-E, which is also a ligand for the activating receptor NKG2C (DOI:10.1016/j.humimm.2015.09.003). However, NKG2C was not included in the receptor panel analyzed in this study.
2. Why were NK cells defined based on NKp80 expression, whereas NKp46 has been widely described as a lineage marker for NK cells (DOI:10.1189/jlb.0903428; DOI:10.1084/jem.186.7.1129)? Importantly, NKp80 expression has been reported to change during viral infections (DOI:10.4049/jimmunol.174.9.5695; DOI:10.1016/j.imbio.2021.152097). Therefore, it is not clear if the use of NKp80 as the defining marker for NK cells influence the identification of NK cell populations in this study and potentially affect the results.
3. Why NK cells were not divided on the basis of CD56 and CD16 expression, which was mentioned to be analysed along the cell phenotyping, yet is abandoned throughout the study?
4. We have concerns regarding the use of a xenogeneic culture system, in which monkey NK cells were co-cultured with the human K562 cell line. First, K562 cells are commonly used in NK cell cytotoxicity assays (Doi:10.3791/56191) rather than as a general stimulus for NK cell activation. Second, it is unclear how the stimulation of monkey NK cells would occur in this xenogeneic model, particularly if it relies on ligand engagement of activating NK cell receptors. The cross-species compatibility of these receptor–ligand interactions is not addressed. Finally, a functional cytotoxicity assay might be more informative than the analysis of intracellular protein expression alone. If the intention was only to stimulate NK cells, why were commonly used NK cell stimulators, such as PMA+ionomycin, not used instead?
5. Some of the observations presented in the manuscript do not appear to be novel. For example, the upregulation of NCRs expression during viral infection and their downregulation after ART treatment (lines 344-346) or that NKG2A-expressing NK cells exhibit higher cytotoxicity potential than NKG2Ahigh cells (line 415). In the latter case, lack the verification of a corelation with CD16/CD56 expression level. Moreover, if the cytotoxicity assay was not performed, how can any conclusions about cytotoxicity be made? Granzyme B expression level or degranulation CD107a marker expression is not enough.
6. Lack of several important information in the Methods section, which must be added, such as about additional 16 non-

infected animals (lines 357-358); animal genotyping methods (lines 366-367); MHC haplotypes (lines 370-371.

Minor:
1. Line 208 – shouldn't there be Live+ instead of Live-?
2. The plots lack error bars (SEM for mean).
3. The applied statistical test used for the analysis should be clearly stated in the figure legend (i.e. whether Spearman or Pearson correlation assay was performed).

Reviewer #2

(Remarks to the Author)
Overall, the manuscript by Chapel et al. that investigate the contribution of NK-cell phenotypes in 12 Mauritian cynomolgus macaques from the primate equivalent (pVISCONTI) of the VISCONTI study in humans is interesting. The authors demonstrate a higher pre-infection (baseline) NKG2AhighNKp30^{-}NKp46^{-} NK cell population linked to improved post-ART interruption SIV control. They also demonstrate that, in contrast, NKG2Alow NKp30^{+}NKp46^{+} cells correlate with poorer SIV control and a larger reservoir.
Innate immunity is understudied in the HIV field.
There are some imitations in this study, that needs to be acknowledged, such as small number of animals and different time of ART initiation which complicates comparison. Nevertheless, this limitation is mitigated by the novel finding that animals with better outcomes post-ART suspension have, as a correlate, a distinct baseline NK cell population, whose frequency is linked to HLA-E.
This correlative and descriptive study would greatly benefit by mechanistic studies addressing how the NKG2Ahigh population influence short- and long-term viral rebound. The investigation on whether the NKG2Ahigh cells affects CD4+T-cell activation and CCR5 expression would be novel and of great interest.
.

Major /Minor

1. More mechanistic studies are needed. Additional experiments such as coculture studies examining whether the NKG2Ahigh and NKG2Alow subsets from naïve animals with the appropriate MHC-IE, affect T-cell activation following SIV envelope stimulation in vitro (expression of CD4 markers, such as Ki67, CCR5 etc)

2. The control (e.g., FMO or isotype) for NKG2A, NKp30 and NKp46 staining should be provided.

4. It should be clarified whether subset frequencies are calculated relative to total NK cells (e.g., NKp80^{+}) or within the NKG2Ahigh and NKG2Alow parent populations. For instance, in Fig. 3B the frequencies are shown within the parent, meaning an increase in one subset will necessarily coincide with a decrease in the other. It would be important to also present these frequencies relative to total live cells or lymphocytes to determine whether the same trends are maintained.

5. Oddly, NKG2Alow cells show greater granzyme B, (which potentially could kill virus infected cells) are positively correlated with viral load. Is the K562 assay irrelevant ? worth a discussion point

6. Are subsets frequencies calculated relative to total NK cells (e.g., NKp80^{+}) or within the NKG2Ahigh and NKG2Alow parent populations. For instance, in Fig. 3B the frequencies are shown within the parent, meaning an increase in one subset will necessarily coincide with a decrease in the other. It would be important to also present these frequencies relative to total live cells or lymphocytes to determine whether the same trends are maintained.

7. In lines 255 and 263, only human references are cited, whereas this study is conducted in Macaca fascicularis. In nonhuman primates, including Macaca nemestrina (pigtailed macaque), NKp80 has been described as an NK-cell marker (doi: 10.1182/blood-2004-12-4762). The authors should complement the current human citations with appropriate macaque-specific references. And if reference for Macaca fascicularis does not exist, it should be clarified by explaining why NKp80-based gating was chosen.

8. The statement in the Methods that "the conclusions are not sex-based" is not supported when only male animals were included. This should be removed or clearly reframed as a study limitation.

9. Figure legends should specify whether lines or bars indicate means or medians, and what denominator is used for each percentage.
10. Line 249: a p-value of 0.0080 does not represent a small difference… may be should be described accordingly.

Reviewer #3

(Remarks to the Author)
I co-reviewed this manuscript with one of the reviewers who provided the listed reports. This is part of the Nature Communications initiative to facilitate training in peer review and to provide appropriate recognition for Early Career Researchers who co-review manuscripts.

Reviewer #4

(Remarks to the Author)
I co-reviewed this manuscript with one of the reviewers who provided the listed reports. This is part of the Nature Communications initiative to facilitate training in peer review and to provide appropriate recognition for Early Career Researchers who co-review manuscripts.

Version 1:

Reviewer comments:

Reviewer #1

(Remarks to the Author)
We thank the authors for their careful revision of the manuscript and for addressing our comments. The manuscript has been substantially improved. However, we would appreciate an additional minor revision before publication.
1. Please include representative control plots (e.g., FMO or isotype controls) for all presented flow cytometry stainings to support the gating strategy and data interpretation. The gating of positive populations for CD161, NKG2D, and DNAM-1 is not sufficiently convincing in its current form.
2. We appreciate the addition of results for other NK cell receptors, DNAM-1, NKG2D, CD161, and CD215, especially for NKG2D and DNAM-1which are among the most important activating receptors in NK biology. However, the rationale for including CD161 and CD215 requires clarification. CD161 is an inhibitory receptor that has attracted attention only recently (DOI: 10.3389/fimmu.2023.1123155; DOI: 10.1111/sji.13090) and is not among the classical NK checkpoint receptors such as TIGIT or Tim-3, which were not analyzed. Similarly, the choice of CD215 should be explicitly justified in the manuscript.

Reviewer #2

(Remarks to the Author)
Thank you for the substantial revisions and for adding new data to address the reviewers' concerns. Few remaining points should be addressed before the manuscript is finalized.

The manuscript appropriately states that the exact mechanism linking NKG2AhighNKp30⁻NKp46⁻ cells to post-treatment control remains to be established. This point should be highlighted more explicitly as a study limitation in the Discussion, especially because the current data remain largely associative and do not directly demonstrate an NKG2A/MHC-E dependent mechanism.
The authors showed in the "response to reviewer" that the subset frequencies were recalculated relative to total live cells or lymphocytes and that the same trends were observed. However, those data were not included in the supplementary figure. They should be added.
In Figure 6E, x-axis seems wrong. It should be NKG2Ahigh and NKG2Alow. Now it says CD4 and NKG2Alow.
The resolution of the FMO/unstained control plots is currently too low. Please provide higher-resolution images. Please include an NKG2A FMO control as well.
The axes in Fig. S2B and Fig. S3C are difficult to read compared with the rest of the manuscript. Please replace these panels with higher-resolution versions.
Both supplemental tables are currently labeled Table S1.
In the Figure 6 legend, "NKp36" appears where it should be "NKp46."Please correct this in the figure legend and check for typo elsewhere in the manuscript.

Reviewer #4

(Remarks to the Author)
I co-reviewed this manuscript with one of the reviewers who provided the listed reports. This is part of the Nature Communications initiative to facilitate training in peer review and to provide appropriate recognition for Early Career Researchers who co-review manuscripts.

Open Access This Peer Review File is licensed under a Creative Commons Attribution 4.0 International License, which permits use, sharing, adaptation, distribution and reproduction in any medium or format, as long as you give appropriate credit to the original author(s) and the source, provide a link to the Creative Commons license, and indicate if changes were made.
In cases where reviewers are anonymous, credit should be given to 'Anonymous Referee' and the source.
The images or other third party material in this Peer Review File are included in the article's Creative Commons license, unless indicated otherwise in a credit line to the material. If material is not included in the article's Creative Commons license and your intended use is not permitted by statutory regulation or exceeds the permitted use, you will need to obtain permission directly from the copyright holder.
To view a copy of this license, visit https://creativecommons.org/licenses/by/4.0/

REVIEWER COMMENTS

Reviewer #1 (Remarks to the Author):

The paper by Chapel et al. investigates an important issue regarding NK cell features and their influence on the treatment of retroviral infection using an animal model. The authors show that the NK cell phenotype observed in SIV-infected monkeys prior to the initiation of ART therapy may influence the course and outcomes of treatment. However, the study is very limited in terms of the range of NK cell receptors analyzed. Although the figures appear visually impressive, the study lacks functional NK cell assays and is largely limited to PBMC/NK cell phenotyping and correlations with viral load. Therefore, we believe that, in its current form, the manuscript does not meet the standards expected for publication in a high-impact journal and would require substantial improvements, if possible.

Major comments:

1. What is the rationale for selecting the specific set of NK cell receptors analyzed in this study, namely the natural cytotoxicity receptors NKp30 and NKp46 and a single inhibitory receptor, NKG2A? This represents a very limited panel of NK cell receptors. On the one hand, several key activating receptors are missing, such as NKG2D, DNAM-1, and 2B4, which have been shown to play important roles in viral infection and immune surveillance. In addition, other relevant inhibitory receptors, including TIGIT, Tim-3, CD161, and PD-1, were not included in the analysis (DOI:10.1093/cei/uxae045; DOI:10.1016/j.jhep.2010.11.030). Recent studies have also shown that DNAM-1–expressing NK cells are upregulated in HIV controllers (DOI:10.1016/j.ebiom.2025.105948), highlighting the potential importance of this receptor in the context of HIV infection.

We thank the reviewer for this important point and agree that a broader assessment of NK cell receptors (including additional activating and inhibitory molecules) can provide complementary information. Our initial panel focused on NKp30 and NKp46 as major natural cytotoxicity receptors and on NKG2A as a key inhibitory receptor that is well characterized in macaques and is reliably detected across longitudinal timepoints using a validated flow cytometry workflow. This choice was also driven by constraints of the cytometer's configuration, sample availability and the need to preserve a consistent multicolor panel across all animals and all timepoints included in the longitudinal analyses.

To directly address the reviewer's concern regarding the limited receptor coverage, we would like to clarify that we used an additional longitudinal panel (not included in the original submission) in which we assessed the surface expression of DNAM-1, NKG2D, CD161, and CD215. We analyzed these data using the same analytical pipeline as for the

main figures (unsupervised clustering), which identified 14 NK-cell clusters, and we evaluated the same key timepoints described in the original manuscript (baseline, day 14 post-infection, day 28 post-infection, pre-ATI, and week 100).

We did not observe phenotypic differences by unsupervised clustering between post-treatment controllers and post-treatment non-controllers (or association with viral loads at the different stages of infection) using this second panel with samples from baseline, day 14, day 28, or pre-ATI. The only differences using this panel were observed at the end of the antiretroviral treatment period, and were associated with the delay to ART initiation: clusters 8 and 10 were increased in early-treated animals, whereas clusters 11, 12, and 14 were increased in late-treated animals.

Importantly, when we examined DNAM-1, NKG2D, CD161, and CD215 by manual gating, we did not detect significant differences between groups, consistent with the unsupervised results.

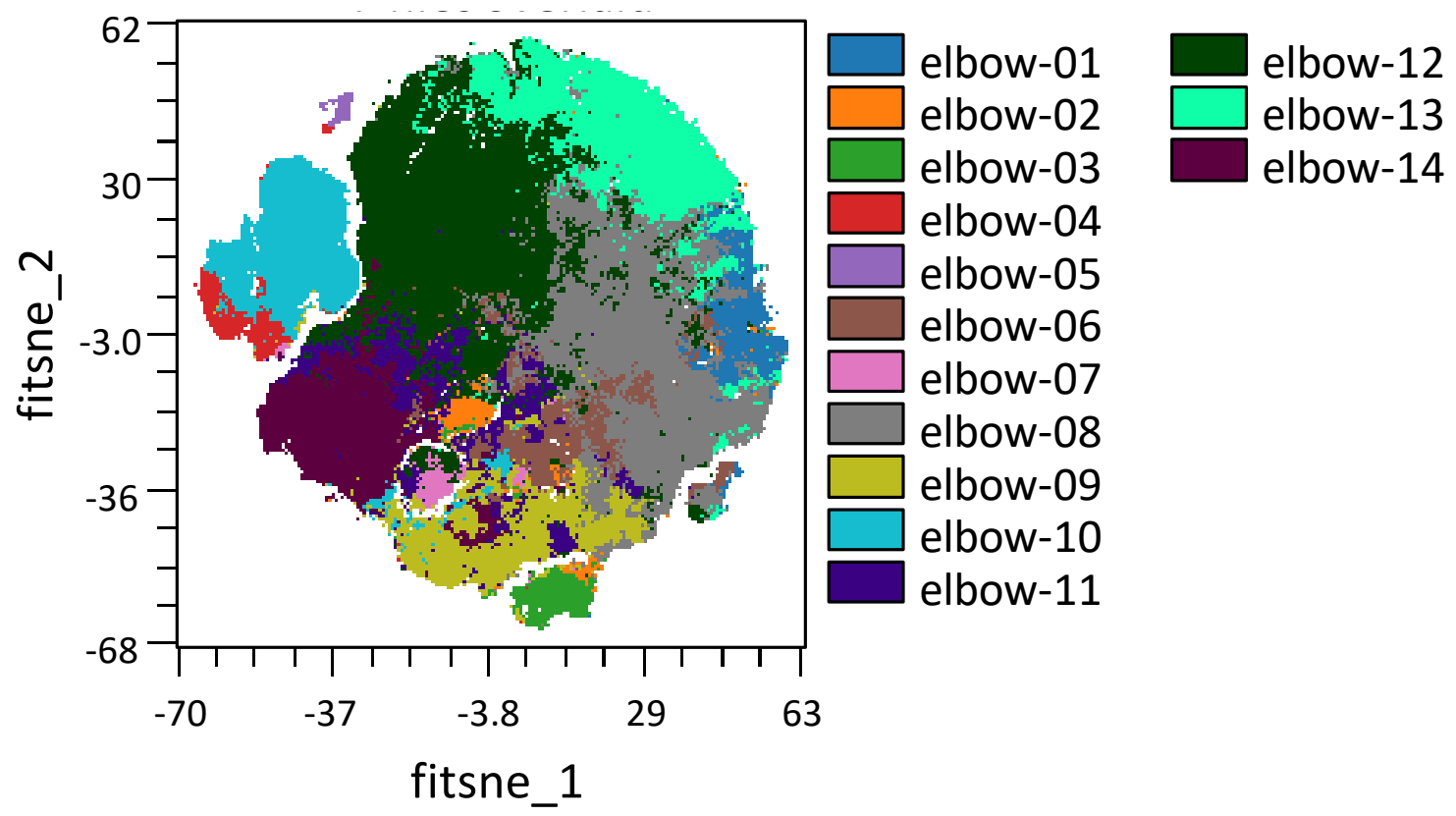

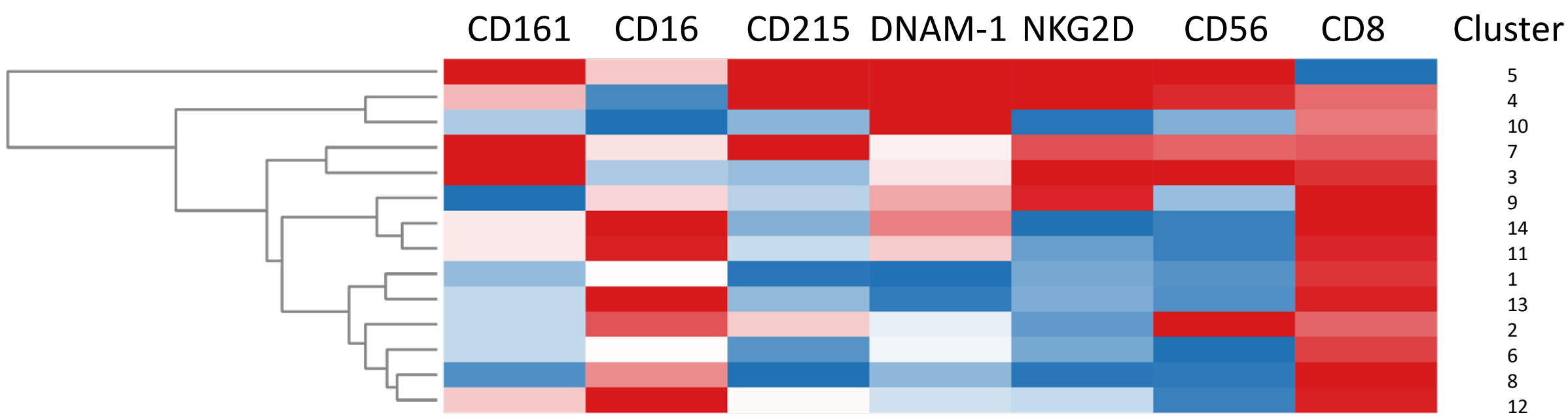

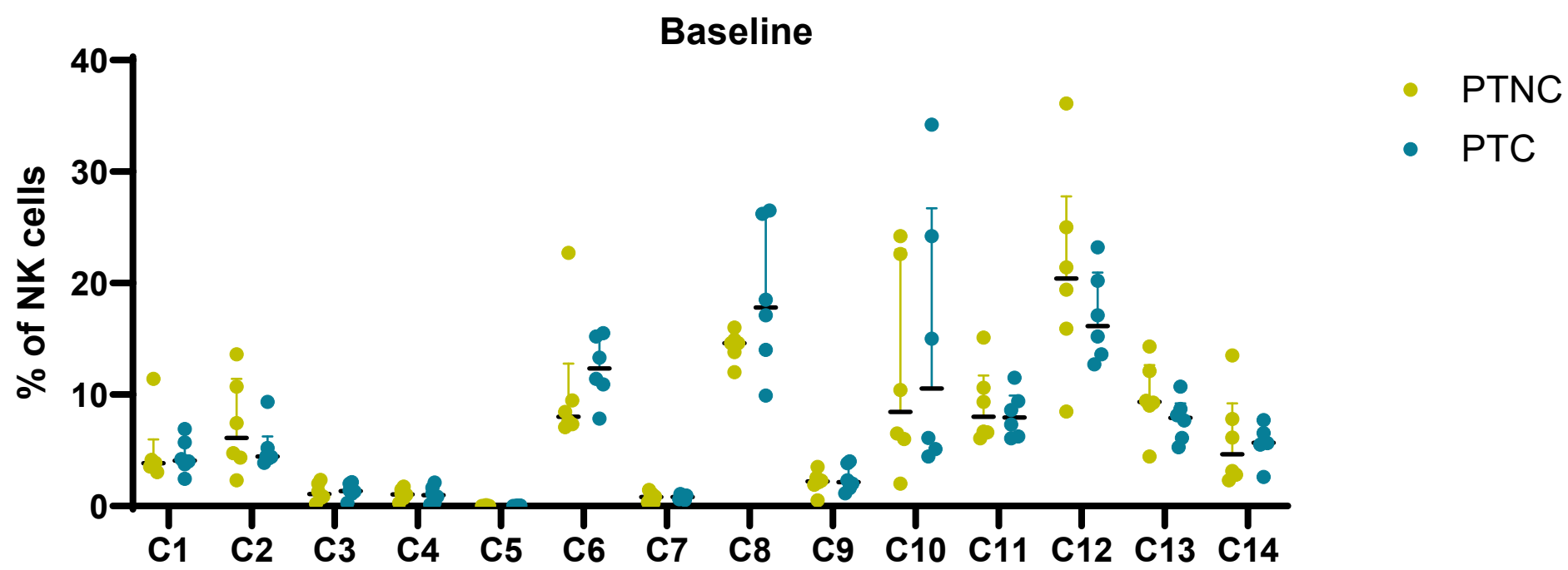

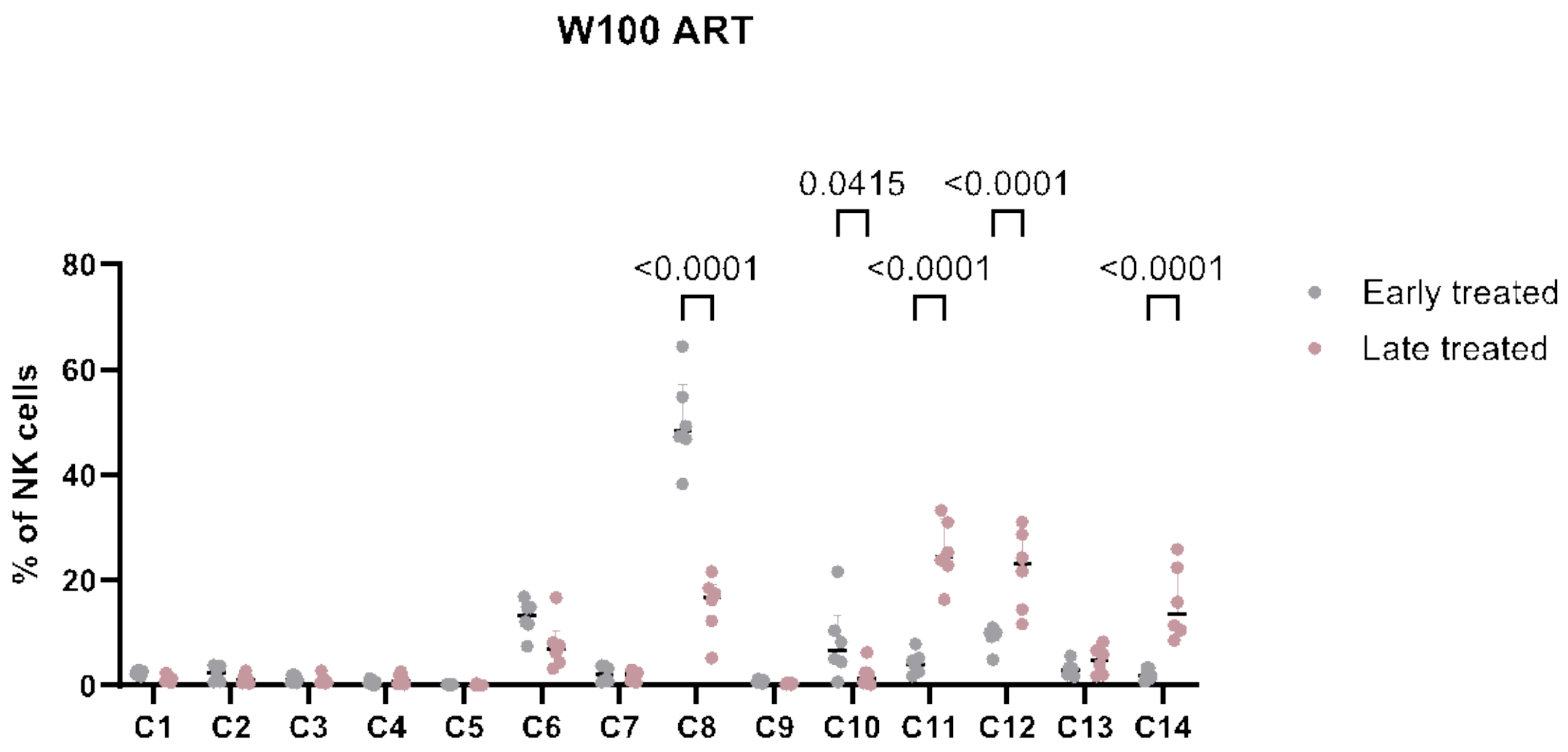
W100 ART
% of NK cells
0.0415
<0.0001
Early treated
Late treated
C1 C2 C3 C4 C5 C6 C7 C8 C9 C10 C11 C12 C13 C14

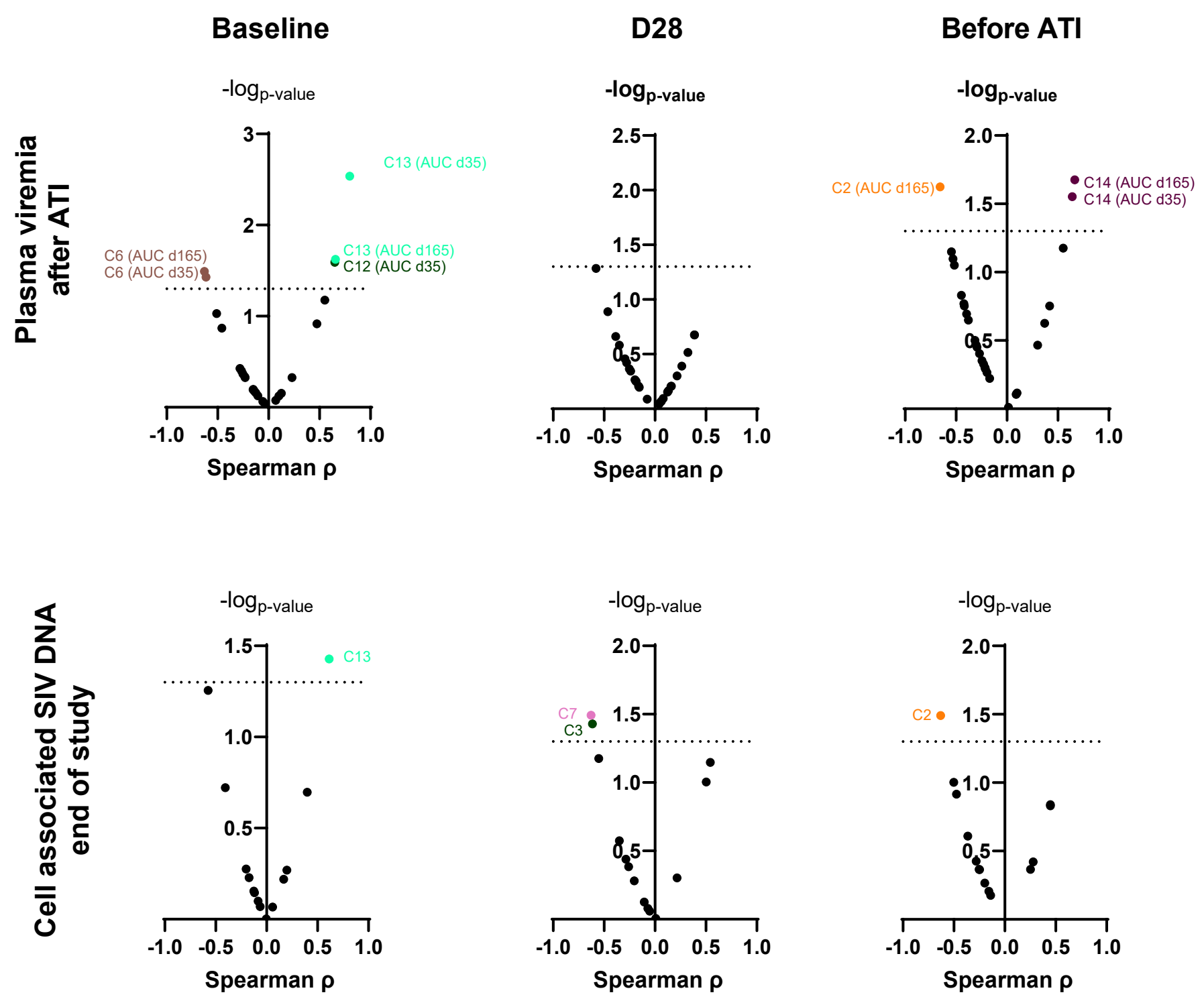
Baseline
D28
Before ATI
Plasma viremia after ATI
Cell associated SIV DNA end of study
-log p-value
Spearman ρ
C13 (AUC d35)
C13 (AUC d165)
C12 (AUC d35)
C6 (AUC d165)
C6 (AUC d35)
C2 (AUC d165)
C14 (AUC d165)
C14 (AUC d35)
C13
C7
C3
C2

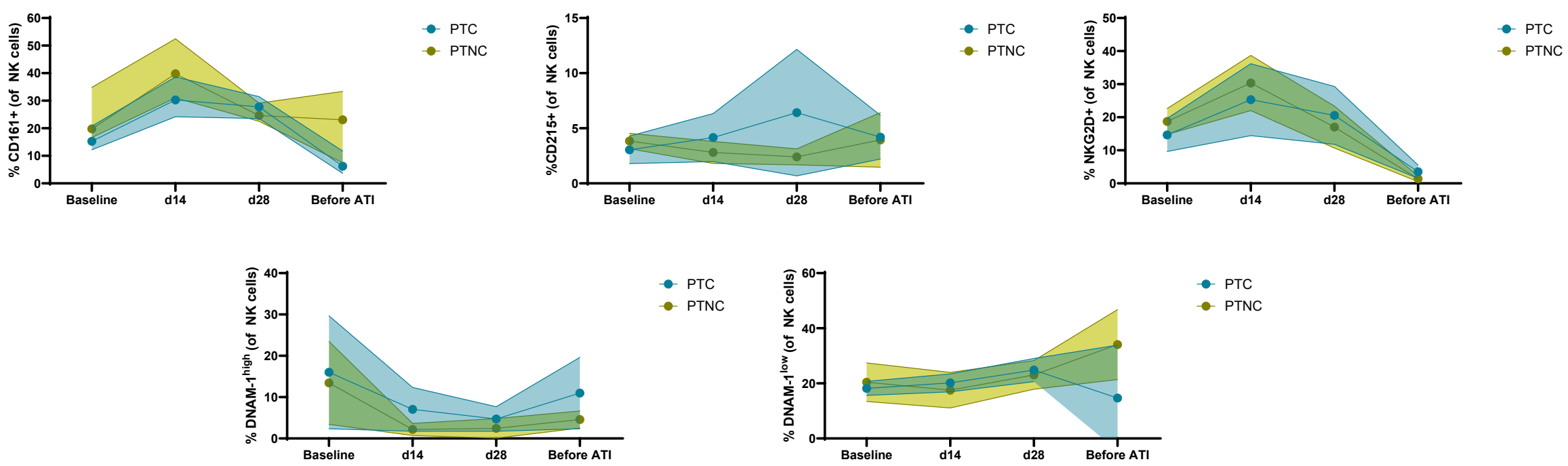
PTC
PTNC
% CD161+ (of NK cells)
%CD215+ (of NK cells)
% NKG2D+ (of NK cells)
% DNAM-1high (of NK cells)
% DNAM-1low (of NK cells)
Baseline
d14
d28
Before ATI

We agree that this information, despite the absence of association with the outcome after ART interruption, is important and offers a more complete description of the NK cell dynamics in this model. We have incorporated this new data as a supplementary figure (Fig. S3) in the revised manuscript.

> On the other hand, the inhibitory receptor NKG2A recognizes HLA-E, which is also a ligand for the activating receptor NKG2C (DOI:10.1016/j.humimm.2015.09.003). However, NKG2C was not included in the receptor panel analyzed in this study.

We agree with the reviewer that NKG2C is highly relevant given its shared ligand (HLA-E) with NKG2A. Unfortunately, a macaque-specific NKG2C antibody is not currently available, preventing reliable inclusion of NKG2C in our panel.

2. Why were NK cells defined based on NKp80 expression, whereas NKp46 has been widely described as a lineage marker for NK cells (DOI:10.1189/jlb.0903428; DOI:10.1084/jem.186.7.1129)? Importantly, NKp80 expression has been reported to change during viral infections (DOI:10.4049/jimmunol.174.9.5695; DOI:10.1016/j.imbio.2021.152097). Therefore, it is not clear if the use of NKp80 as the defining marker for NK cells influence the identification of NK cell populations in this study and potentially affect the results.

We thank the reviewer for this important question. We agree that NK cell identification in NHP is less standardized than in humans, and that NKp46 has been widely used as a lineage-associated marker in several species. However, NKp46 expression is labile during infection (DOI: 10.1002/eji.200324141 or Figure S6 in our manuscript). In our study, we defined NK cells using NKp80 within a broader lineage-exclusion strategy. This was first based on empirical validation in our hands during the development and optimization of a customized magnetic Non-Human Primate NK cell enrichment kit with the Miltenyi Biotec technical team. We produced a depletion cocktail targeting T cells, B cells, monocytes, and dendritic cells. After antibody titration and optimization, we observed an appreciable fraction of cells with NK-like characteristics, that were NKG2D and NKG2A negative but expressed NKp80. Moreover, NKp80 expression uniformly captured NK cell populations independently of NKG2A or NKG2D expression. The use of NKp80 to identify NK cells was comforted by literature supporting NKp80 as a robust specific NK cell marker, including in other species of macaques (DOI: 10.3389/fimmu.2024.1423689, 10.1016/j.isci.2020.101298, 10.1182/blood-2004-12-4762).

We have included as Fig. S1 results showing the validation of the NK cell enrichment kit and the use of NKp80 for identifying NK cells.

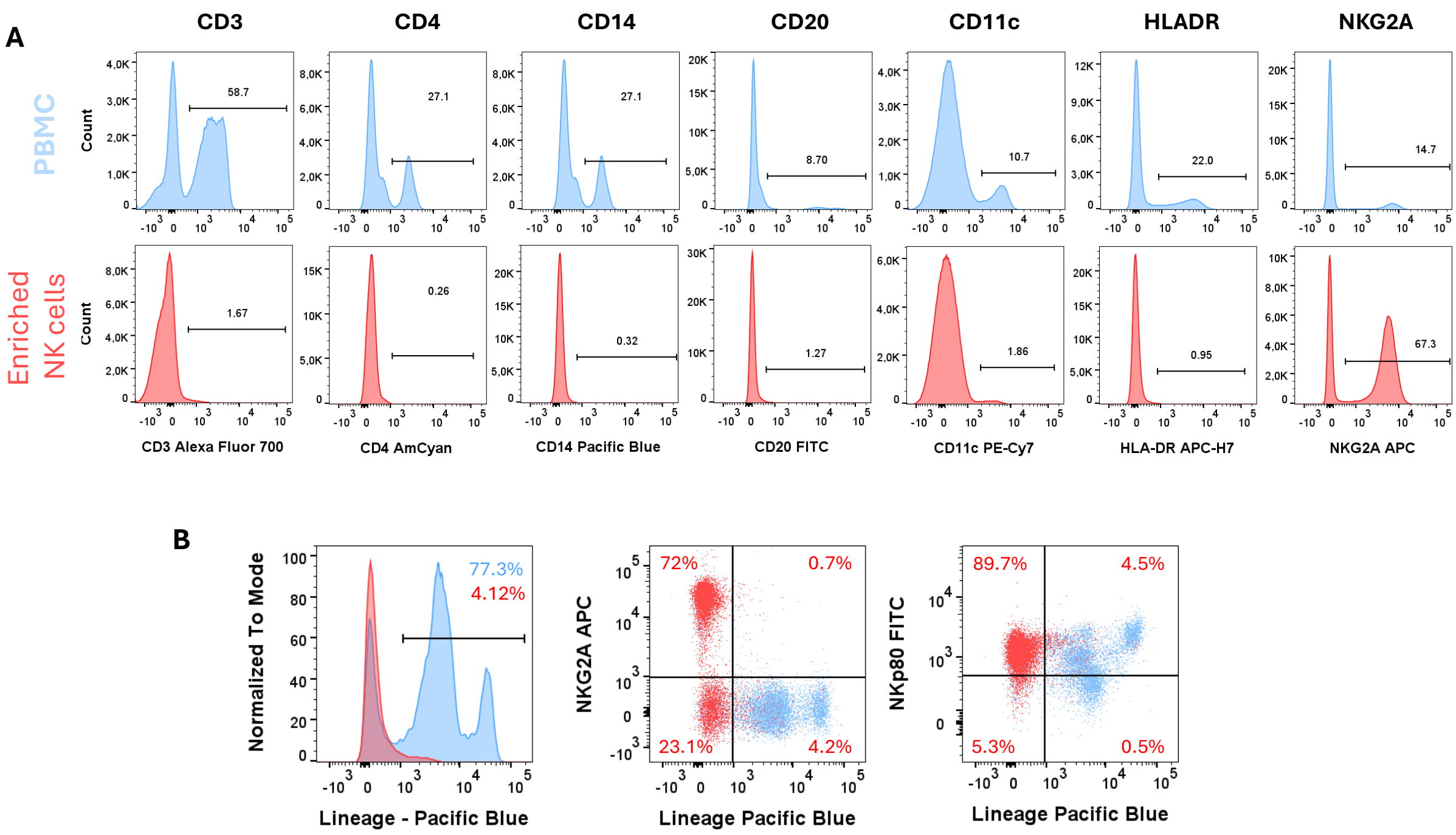

3. Why NK cells were not divided on the basis of CD56 and CD16 expression, which was mentioned to be analysed along the cell phenotyping, yet is abandoned throughout the study?

We thank the reviewer for allowing us to clarify this point. Although CD56 and CD16 were included in our phenotyping panels, we did not use them as primary markers to subdivide NK cells because CD56 and CD16 do not provide a robust or NK-specific framework in macaques and can lead to misleading subset definitions.

Specifically, CD56 is not a reliable NK-cell marker in macaques, where it has been shown to predominantly identify monocytes rather than NK cells (Carter et al., Cytometry, 1999 PMID: 10451505). This limitation is also consistent with later work emphasizing that CD56 is not an appropriate surface antigen for NK-cell identification in most simian species (DOI: 10.1182/blood-2004-12-4762). In addition, CD16 is not NK-cell specific in nonhuman primates, as $CD16^+$ monocyte subsets can phenotypically overlap with NK populations (DOI: 10.1182/blood.V88.4.1215.bloodjournal8841215), and $CD16^+$ monocytes have been reported across multiple simian studies (10.1182/blood-2004-12-4762).

As an illustration, we provide below a figure showing a comparison of CD56/CD16 staining in cynomolgus macaque vs human samples, showing that CD56 marks only a small fraction of NK cells in cynomolgus macaques, consistent with the published limitations of CD56 in NHPs.

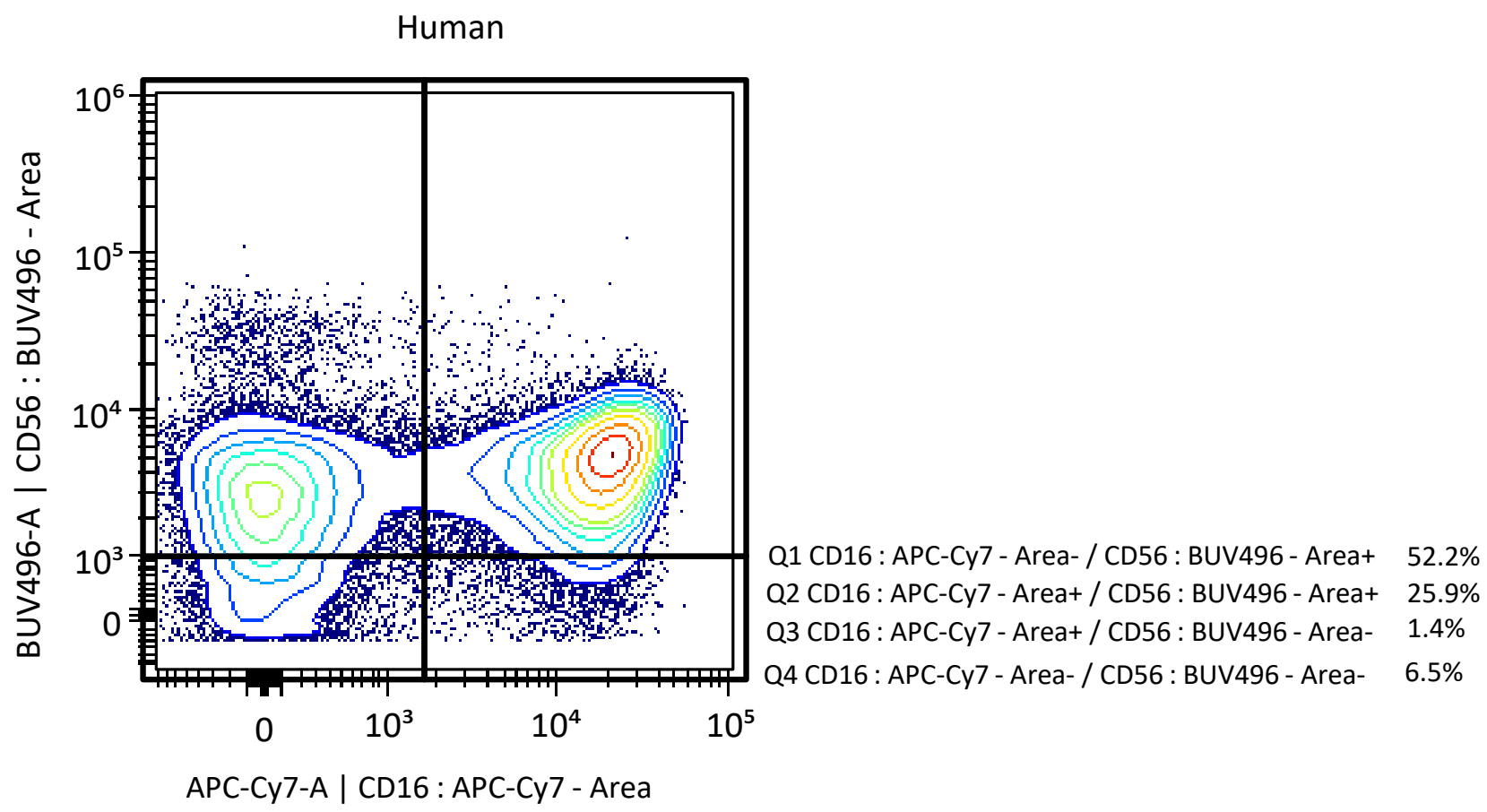

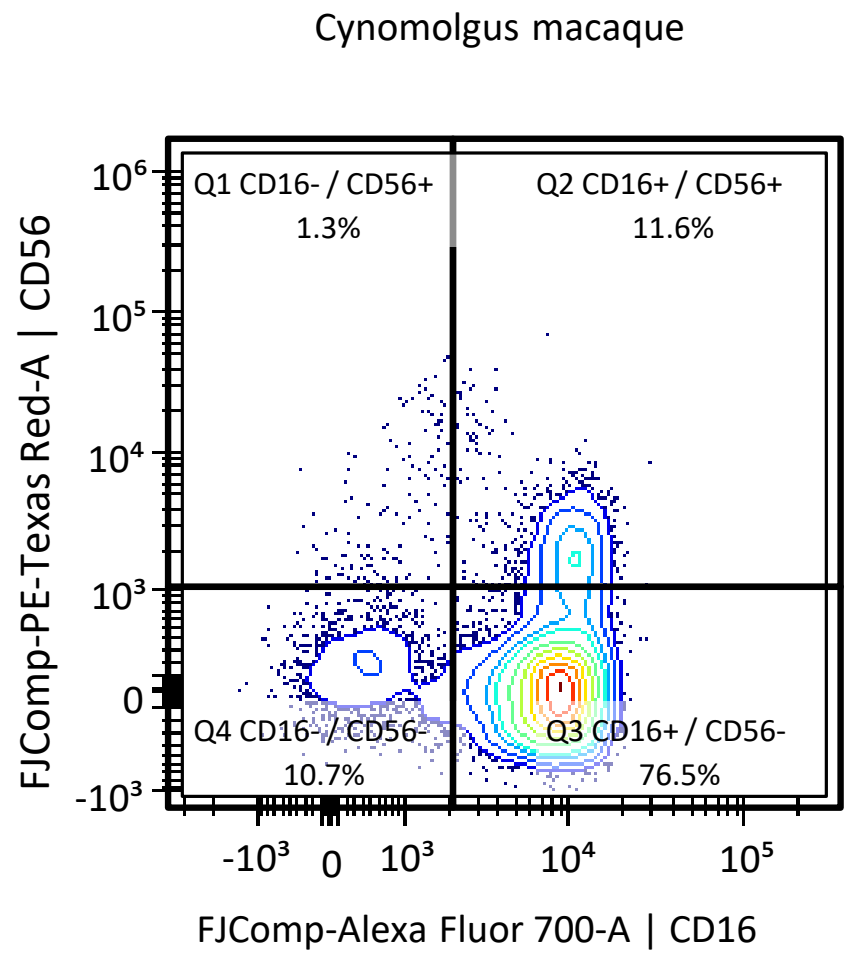

4. We have concerns regarding the use of a xenogeneic culture system, in which monkey NK cells were co-cultured with the human K562 cell line. First, K562 cells are commonly used in NK cell cytotoxicity assays (Doi:10.3791/56191) rather than as a general stimulus for NK cell activation. Second, it is unclear how the stimulation of monkey NK cells would occur in this xenogeneic model, particularly if it relies on ligand engagement of activating NK cell receptors. The cross-species compatibility of these receptor–ligand interactions is not addressed. Finally, a functional cytotoxicity assay might be more informative than the analysis of intracellular protein expression alone. If the intention was only to stimulate NK cells, why were commonly used NK cell stimulators, such as PMA+ionomycin, not used instead?

We thank the reviewer for this comment and for the opportunity to clarify our experimental rationale. K562 co-culture is a long-established, widely used functional assay to evaluate NK-cell responsiveness (degranulation and cytokine production) in both humans and non-human primates, and it is commonly used not only for cytotoxicity readouts but also as a physiologic target-cell stimulus to induce NK degranulation and

effector cytokine production measured by flow cytometry (e.g., CD107a mobilization and intracellular cytokines), as described in standard protocols (e.g., DOI: 10.3791/56191 for degranulation; DOI: 10.1182/blood-2009-08-238469 for cytokine production), including in NHP (DOI: 10.1038/s41590-023-01661-4; 10.1128/JVI.00731-19).

K562 targets are indeed classically used for cytotoxicity assays; however, the same co-culture system is also routinely used to trigger NK effector functions that are then quantified by flow cytometry, including CD107a mobilization and intracellular accumulation of effector molecules/cytokines in the presence of secretion inhibitors. In our study, we used K562 precisely as an NK-sensitive target to elicit a standardized, biologically relevant activation/degranulation response in the absence of the inhibitory signals (the "missing self" paradigm).

The reviewer's concern about cross-species ligand engagement is understandable. However, K562-mediated NK activation does not rely on a single activating receptor–ligand pair. On the contrary, K562 cells are highly susceptible to NK cells primarily because they do not express (or only at very low levels) MHC class I ligands, thereby failing to engage inhibitory NK receptors ("missing-self" DOI: 10.1038/ni0508-477). In this setting, relief from major inhibitory signaling is a major driver of NK activation. Robust activation of rhesus/cynomolgus NK cells by K562 has been demonstrated extensively.

In our dataset, K562 co-culture produced the expected functional readouts (increased degranulation/effector signals compared with unstimulated controls), which is shown in the gating strategy/representative plots. We provide below for the reviewer consideration an illustrative human PBMC example processed in the same way, showing that K562 similarly induced cytokine production and degranulation. In particular, we observe reduced intracellular granzyme levels after 4 hours, consistent with granule release, in agreement with the concurrent degranulation readouts shown in the manuscript with macaque NK cells.

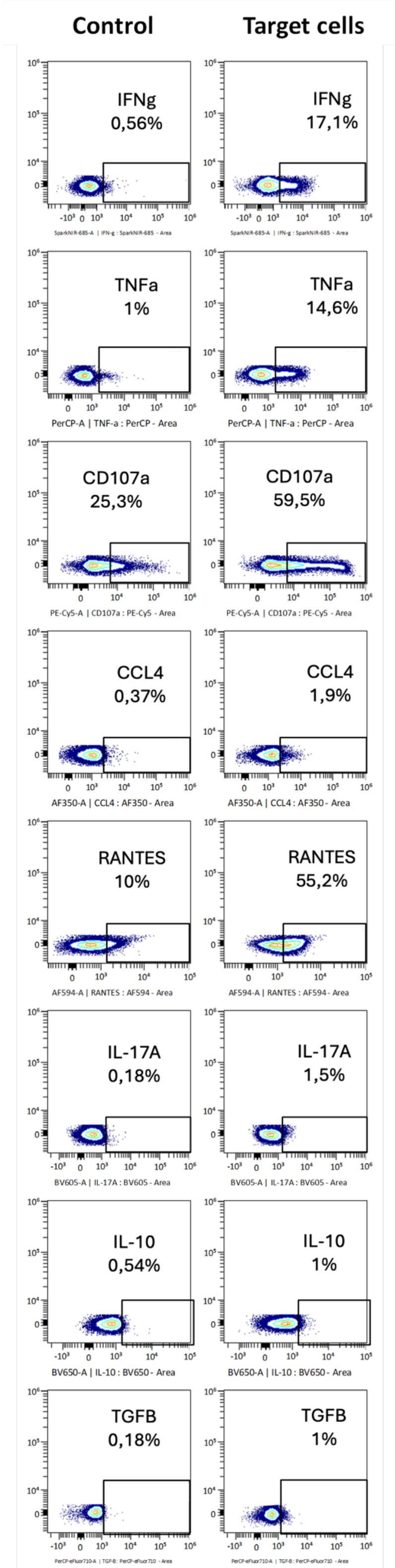

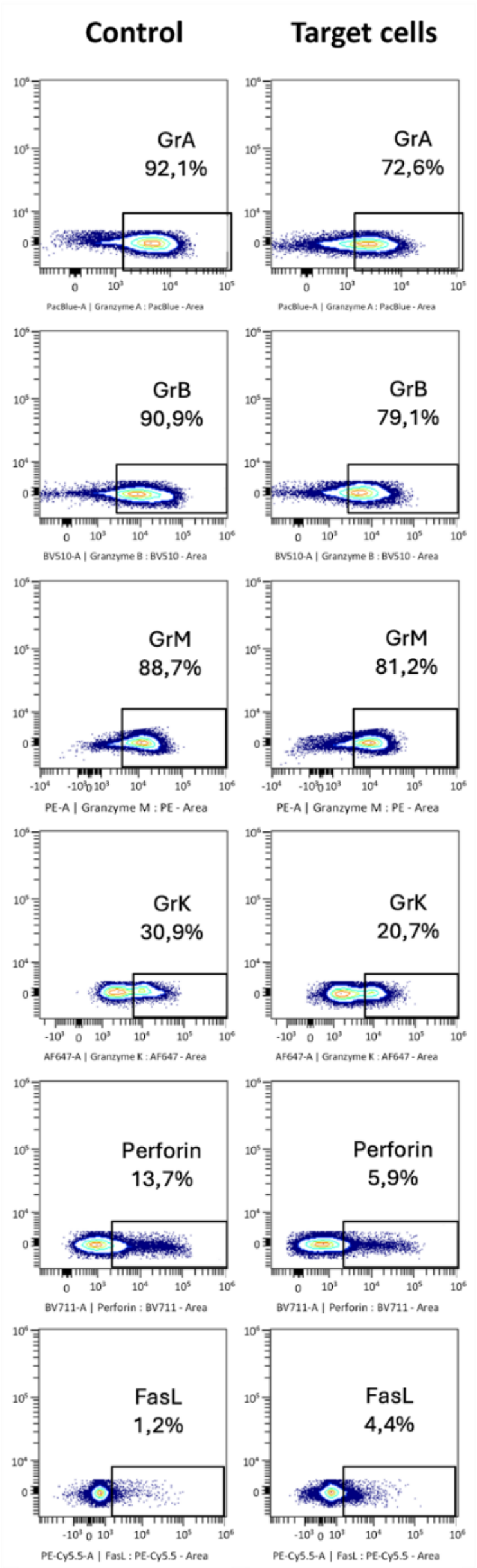

We agree that direct killing assays (e.g., chromium release, flow-based killing of labeled targets) provide complementary information. Our goal here, however, was to quantify NK functional potential at the single-cell level in a manner compatible with limited longitudinal sample volumes and multiparametric phenotyping. CD107a mobilization

and intracellular cytokine staining are widely accepted functional correlates of NK effector responses (DOI: 10.1016/j.cellimm.2008.08.007), and they provide mechanistic insight into degranulation/cytokine capacity even when cell numbers are limiting.

Finally, PMA/ionomycin is a powerful, non-physiological pharmacologic stimulus that bypasses receptor-proximal signaling and can mask biologically meaningful differences in upstream activation thresholds. Because our objective was to evaluate NK responses elicited by a target-cell encounter (as infected autologous CD4+ T-cells would be), we chose K562 co-culture as a more physiologically relevant and commonly used stimulus for NK effector function. Critically, to validate that this K562-induced response reflects true antiviral potential, we have now performed in vitro SIV suppressive capacity assays, coculturing sorted NK cells with autologous SIV-infected CD4+ T-cells (as the physiological target). We observed that NK cells cocultured with infected CD4+ T-cells showed upregulation of specific effector molecules (CCL4, GrB, CD107a, IL-10, and Perforin) similar to the K562 assay (new Fig. 6D). Overall the results obtained with the K562 system were consistent with the NK behavior against virus-infected targets in our experiments, justifying its use over pharmacologic stimulants.

5. Some of the observations presented in the manuscript do not appear to be novel. For example, the upregulation of NCRs expression during viral infection and their downregulation after ART treatment (lines 344-346) or that NKG2A-expressing NK cells exhibit higher cytotoxicity potential than NKG2Ahigh cells (line 415). In the latter case, lack the verification of a corelation with CD16/CD56 expression level. Moreover, if the cytotoxicity assay was not performed, how can any conclusions about cytotoxicity be made? Granzyme B expression level or degranulation CD107a marker expression is not enough.

We thank the reviewer for this thoughtful comment. We agree that some of the directional changes we describe (e.g., modulation of NCR expression during untreated infection and after ART) have been reported in prior HIV/SIV studies, which is important to validate our approach. We have revised the text to more clearly link these results with previous observations. We would like however to highlight the novelty of our work, namely the longitudinal, pre-ART/early infection NK phenotype associations with subsequent outcomes after ART interruption in this macaque model (and the specific timing-dependent trajectories we report).

We acknowledge that we did not perform a direct killing assay (e.g., target cell lysis) and we have revised the text throughout to refer to “degranulation and cytotoxic effector potential” or “functional responsiveness”, as supported by our data (e.g., CD107a mobilization and granzyme/perforin content, where applicable).

As discussed in our response to Comment 3, CD56 is not a reliable marker for NK cells in macaques, and CD16 is also not NK-specific in NHPs. Therefore, CD56/CD16 cannot be used as primary stratification variables.

6. Lack of several important information in the Methods section, which must be added, such as about additional 16 non-infected animals (lines 357-358); animal genotyping methods (lines 366-367); MHC haplotypes (lines 370-371.

We thank the reviewer for pointing out this oversight concerning methodological details. We have revised the manuscript accordingly. We have added the missing individual animal information (including age and sex) in Table S2.

We have added a description of the animal genotyping procedures in the Methods section (Line 527-532) and the genotype for all the animals (Tables S1 and S2).

Minor:

1. Line 208 – shouldn't there be Live+ instead of Live-?

We thank the reviewer for catching this. This is a labeling issue. In our gating strategy we used a fixable viability dye to exclude dead cells. We gated on the viability-dye^{negative} (Live/Dead^{-}) population, which corresponds to live cells. To avoid any ambiguity, we will revise the text at line 555 to explicitly state "Viable".

2. The plots lack error bars (SEM for mean).

We thank the reviewer for this suggestion. In the current figures we intentionally display all individual data points and use a horizontal line to indicate the median, now clarified in the legend, which we felt represents better the distribution and variability given the relatively small group sizes.

3. The applied statistical test used for the analysis should be clearly stated in the figure legend (i.e. whether Spearman or Pearson correlation assay was performed).

- We thank the reviewer for emphasizing this point. The correlation test used (Spearman) is now specified in the figure legends in the revised version of the manuscript.
- To further improve clarity and avoid any possibility of confusion, we have verified that all the figure legends explicitly state now the test used for statistic analyses.

Reviewer #2 (Remarks to the Author):

Overall, the manuscript by Chapel et al. that investigate the contribution of NK-cell phenotypes in 12 Mauritian cynomolgus macaques from the primate equivalent (pVISCONTI) of the VISCONTI study in humans is interesting. The authors demonstrate a higher pre-infection (baseline) NKG2AhighNKp30^{-}NKp46^{-} NK cell population linked to improved post-ART interruption SIV control. They also demonstrate that, in contrast, NKG2Alow NKp30^{+}NKp46^{+} cells correlate with poorer SIV control and a larger reservoir.
Innate immunity is understudied in the HIV field.
There are some imitations in this study, that needs to be acknowledged, such as small number of animals and different time of ART initiation which complicates comparison. Nevertheless, this limitation is mitigated by the novel finding that animals with better outcomes post-ART suspension have, as a correlate, a distinct baseline NK cell population, whose frequency is linked to HLA-E.
This correlative and descriptive study would greatly benefit by mechanistic studies addressing how the NKG2Ahigh population influence short- and long-term viral rebound. The investigation on whether the NKG2Ahigh cells affects CD4+T-cell activation and CCR5 expression would be novel and of great interest.

Major /Minor

1. More mechanistic studies are needed. Additional experiments such as coculture studies examining whether the NKG2Ahigh and NKG2Alow subsets from naïve animals with the appropriate MHC-IE, affect T-cell activation following SIV envelope stimulation in vitro (expression of CD4 markers, such as Ki67, CCR5 etc)

We agree with the reviewer that additional mechanistic experiments are important to strengthen the conclusions. Despite important sample limitations, we could perform ex vivo coculture experiments using sorted NKG2Ahigh and NKG2Alow NK-cell subsets and autologous SIV-infected CD4^{+} T cells with samples from 8 animals (new Fig. 6D–G and Fig. S13 & S14). In these assays, we evaluated viral replication (p27 in supernatants), NK-cell effector responses, and CD4+ T-cell activation and phenotype, including CCR5 and Ki-67 expression. We observed that NKG2Ahigh NK cells produced higher levels of CCL4 and lower levels of IL-10 than NKG2Alow NK cells in response to infected targets, which is highly coherent with our previous observation using K562 cells as targets. Coculture of infected cells with NKG2Ahigh, but not with NKG2Aneg or NKG2Alow NK cells, was consistently associated with reduced p27 levels. The capacity of NK cell subsets to suppress infection was positively correlated with their capacity to

produce CCL4 and negatively correlated with their IL-10 production. In addition, the presence of NKG2Ahigh NK cells was associated with a sharp reduction of CCR5 expression on CD4+ T cells. We have incorporated this new data and the corresponding interpretation into the Results (Lines 494-515) and Discussion (Lines 315-335, 400-419) sections, and updated the figures accordingly.

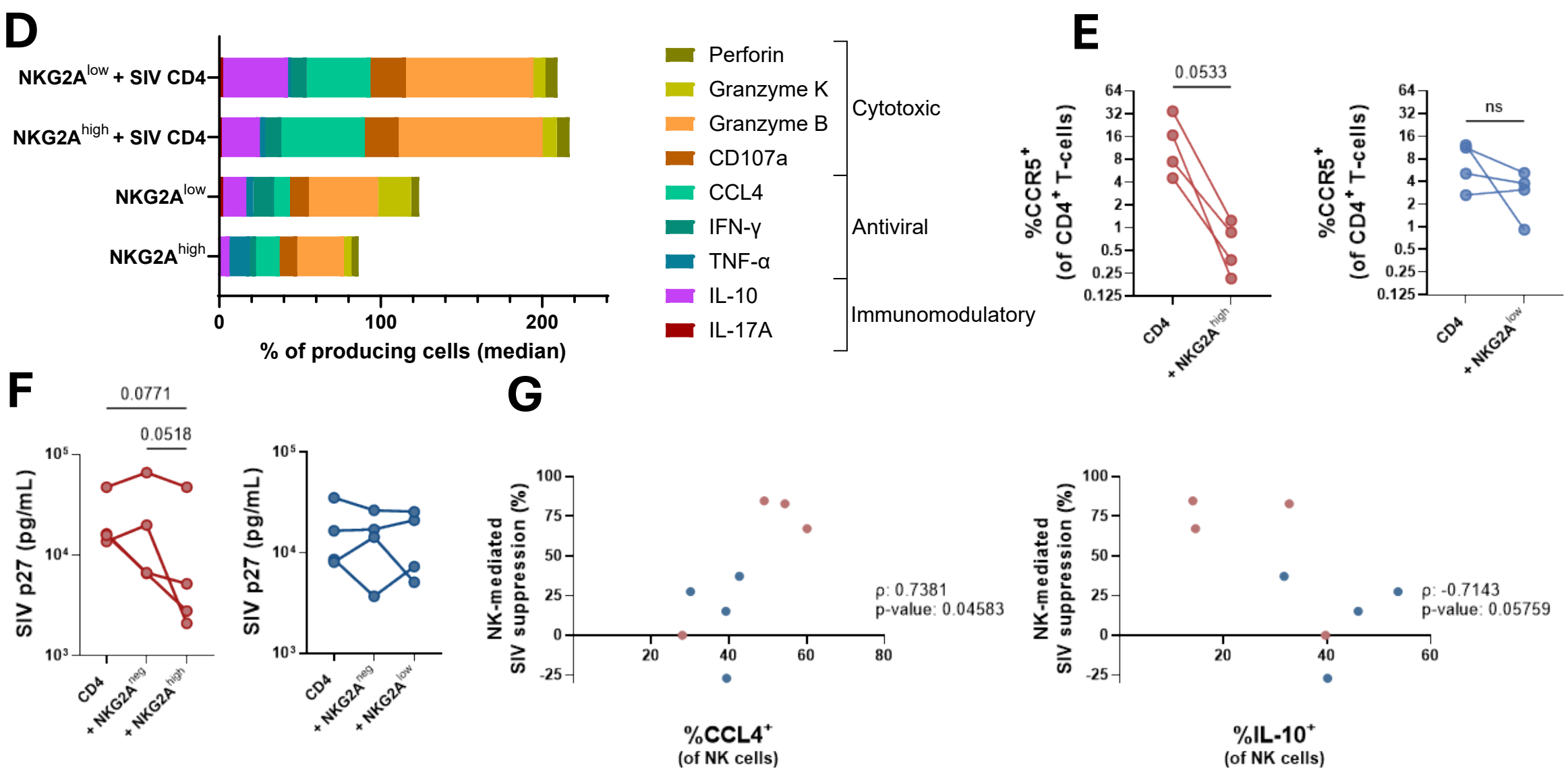

2. The control (e.g., FMO or isotype) for NKG2A, NKp30 and NKp46 staining should be provided.

We appreciate this suggestion. We have now included unstained controls for NKp30 and NKp46 to document gate placement (Fig. S2). For NKG2A, we used a well-resolved bimodal staining pattern across samples and set gates based on the negative population; we now clarify this gating approach in the Methods. If the reviewer prefers, we can also add an NKG2A FMO in the revised supplement.

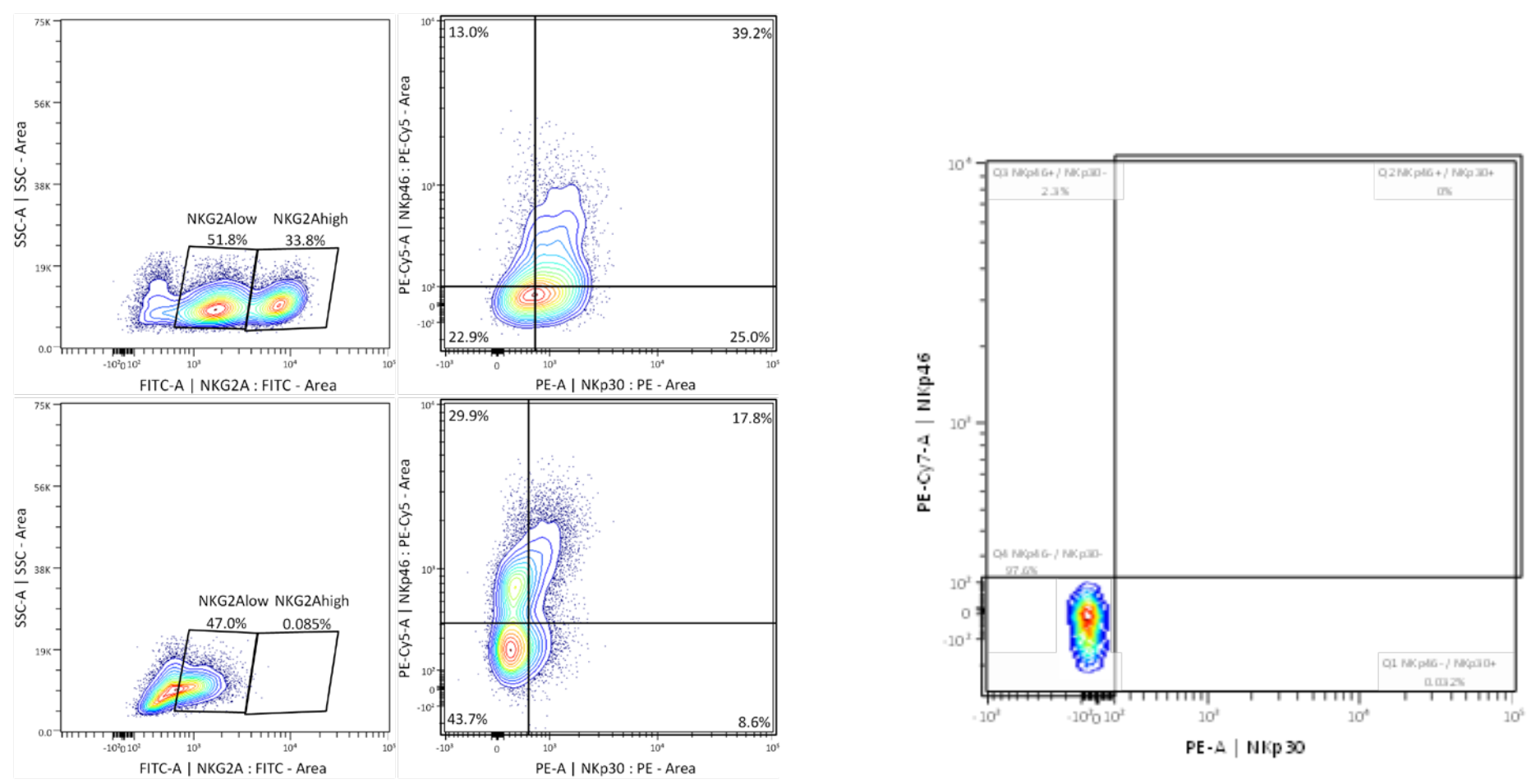

3. It should be clarified whether subset frequencies are calculated relative to total NK cells (e.g., NKp80$^+$) or within the NKG2Ahigh and NKG2Alow parent populations. For instance, in Fig. 3B the frequencies are shown within the parent, meaning an increase in one subset will necessarily coincide with a decrease in the other. It would be important to also present these frequencies relative to total live cells or lymphocytes to determine whether the same trends are maintained.

In Fig. 3B, the two subsets are not mutually exclusive within a single parent gate. Specifically, the NKp30$^-$NKp46$^-$NKG2A^{high} population is quantified within the NKG2A^{high} parent, whereas the NKp30$^+$NKp46$^+$NKG2A^{low} population is quantified within the NKG2A^{low} parent. Therefore, an increase in one subset does not mathematically require a decrease in the other, since they are referenced to different parent populations.

To directly address the reviewer's request, we recalculated both populations as frequencies relative to total live lymphocytes. This alternative normalization shows the same trends as the parent-normalized representation. We have explicitly stated the denominator used in the corresponding figure legends.

% of Live cells (instead of parent)

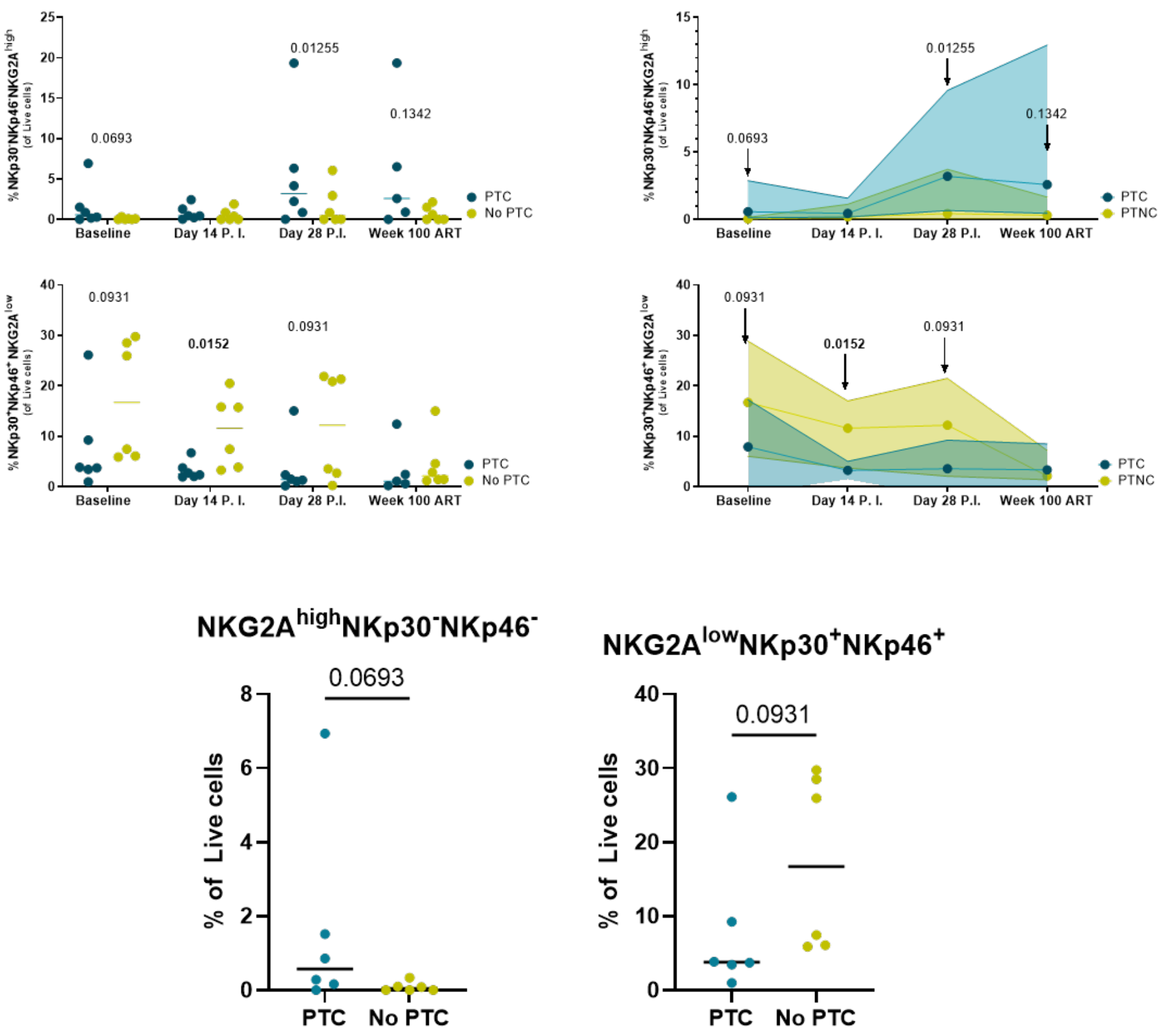

4. Oddly, NKG2Alow cells show greater granzyme B, (which potentially could kill virus infected cells) are positively correlated with viral load. Is the K562 assay irrelevant ? worth a discussion point

The K562 assay remains relevant as a standardized cellular stimulus that quantifies NK functional responsiveness (e.g., degranulation and cytokine production capacity) at the single-cell level, and the granzyme B content has been shown as a good correlate for cytotoxic potential in multiple settings. We agree that our results may appear paradoxical in this regard, but they reflect a complex environment where direct cytotoxic potential would not be the sole factor associated with viral control. NKG2A^{high} cells were associated with better control of infection after ART interruption, and we show in the new co-culture data that this cell subset has direct antiviral activity against infected autologous CD4+ T cells. We found that NKG2A^{high} cells, although having low basal GrzB content, were not only able to strongly upregulate this molecule in response to infected cells but also abundantly produced CCL4, which was associated with CCR5 downregulation. Moreover, we have also shown that NKG2A^{high} cells express lymph node homing markers and may potentially exert antiviral activities in tissues relevant for SIV infection. In addition, this relatively immature cell subset may favor the honing of adaptive immune responses, reinforcing the mechanisms of viral control. In contrast, NKG2A^{low} cells, in particular those co-expressing NKp30 and NKp46 may provide a negative environment for the development of adaptive immunity through the elimination of key T helper or antigen presenting cell subsets, or the production of IL-10, which has been also associated with the establishment and persistence of the HIV-1 reservoir. Our results underline the functional plasticity of NK cells and that they need to be considered beyond their classic cytotoxic potential. The mechanisms (and the characteristics of cells involved) of viral control after ART interruption, when the virus is already suppressed, may differ from those necessary to actively control ongoing viral replication.

5. Are subsets frequencies calculated relative to total NK cells (e.g., NKp80^{+}) or within the NKG2Ahigh and NKG2Alow parent populations. For instance, in Fig. 3B the frequencies are shown within the parent, meaning an increase in one subset will necessarily coincide with a decrease in the other. It would be important to also present these frequencies relative to total live cells or lymphocytes to determine whether the same trends are maintained.

We thank the reviewer for emphasizing this point. As clarified in our response to Comment 3, the subsets in Fig. 3B are quantified within different parent gates (NKG2A^{high} vs NKG2A^{low}) and therefore are not mutually exclusive within a single parent population; an increase in one does not mathematically require a decrease in the other.

To address the reviewer's request, we additionally calculated the same subset frequencies relative to total live lymphocytes (and/or total live cells) and observed the same directional trends as in the parent-normalized analysis (see response above).

6. In lines 255 and 263, only human references are cited, whereas this study is conducted in Macaca fascicularis. In nonhuman primates, including Macaca nemestrina (pigtailed macaque), NKp80 has been described as an NK-cell marker (doi: 10.1182/blood-2004-12-4762). The authors should complement the current human citations with appropriate macaque-specific references. And if reference for Macaca fascicularis does not exist, it should be clarified by explaining why NKp80-based gating was chosen.

We thank the reviewer for this important question. We agree that NK-cell identification in nonhuman primates is less standardized than in humans, and that NKp46 has been widely used as a lineage-associated NK marker in several species. In our study, however, NK cells were defined using NKp80 within a broader lineage-exclusion strategy. This choice was guided by published work supporting NKp80 as a robust NK marker, including in macaques (doi: 10.1182/blood-2004-12-4762) and humans (doi: 10.1016/j.isci.2020.101298; doi: 10.3389/fimmu.2024.1423689), and empirical validation during optimization of our customized Miltenyi (MACS) magnetic enrichment/sorting workflow. In addition, we appreciate the reviewer highlighting the macaque-specific evidence in *Macaca nemestrina* (doi: 10.1182/blood-2004-12-4762), and we have now included this reference in the revised manuscript to complement the human-focused citations.

Specifically, during development of our custom MACS NK enrichment kit, we used a depletion cocktail targeting T cells, B cells, monocytes, and dendritic cells, followed by antibody titration/optimization and flow-based assessment of the remaining "NK-like" compartment. We observed that, despite effective depletion of classical monocytes, an appreciable fraction of NK-like cells was NKG2A−. Importantly, these NKG2A− NK-like cells were uniformly NKp80+, indicating that NKp80 captured an NK population that would have been missed by an NKG2A-based definition alone. We have added the macaque-specific reference and clarified this rationale in the revised manuscript.

7. The statement in the Methods that "the conclusions are not sex-based" is not supported when only male animals were included. This should be removed or clearly reframed as a study limitation.

The reviewer is right to pint out that we cannot draw any conclusions regarding sex as a biological variable, because only male animals were included. We have removed the statement that "the conclusions are not sex-based".

8. Figure legends should specify whether lines or bars indicate means or medians, and what denominator is used for each percentage.

We have updated the figure legends to explicitly state whether the lines/bars represent means or medians, and to specify the denominator used for each percentage.

9. Line 249: a p-value of 0.0080 does not represent a small difference… may be should be described accordingly.

We have revised the wording in the manuscript (line 115) to avoid describing this result as a "small difference" and instead report it as a statistically significant difference ($p = 0.0080$) without implying a small effect size.

Reviewer #1 (Remarks to the Author):

We thank the authors for their careful revision of the manuscript and for addressing our comments. The manuscript has been substantially improved. However, we would appreciate an additional minor revision before publication.

1. Please include representative control plots (e.g., FMO or isotype controls) for all presented flow cytometry stainings to support the gating strategy and data interpretation. The gating of positive populations for CD161, NKG2D, and DNAM-1 is not sufficiently convincing in its current form.

To better support the gating strategy and data interpretation, we have now included unstained samples for CD161, NKG2D, DNAM-1, and CD215. In addition to the unstained controls, we have used the expression of these markers on non-NK cell lymphocytes as an internal reference to further validate and adjust the gating thresholds. Both strategies are now presented and described in the updated Figure S3, providing a more rigorous justification of the gates applied throughout the study.

2. We appreciate the addition of results for other NK cell receptors, DNAM-1, NKG2D, CD161, and CD215, especially for NKG2D and DNAM-1which are among the most important activating receptors in NK biology. However, the rationale for including CD161 and CD215 requires clarification. CD161 is an inhibitory receptor that has attracted attention only recently (DOI: 10.3389/fimmu.2023.1123155; DOI: 10.1111/sji.13090) and is not among the classical NK checkpoint receptors such as TIGIT or Tim-3, which were not analyzed. Similarly, the choice of CD215 should be explicitly justified in the manuscript.

We appreciate this thoughtful comment and agree that the rationale for the selection of each receptor warrants explicit justification in the manuscript. We have now added the appropriate references and explanatory text in the revised manuscript. DNAM-1, NKG2D and CD161 have been described as upregulated in NK cells from HIV-1 elite controllers compared to non-controllers in high-dimensional analyses (DNAM-1 and NKG2D: doi: 10.1016/j.ebiom.2025.105948; CD161: doi: 10.1128/JVI.01790-18), providing direct relevance to the context of our study. CD215 (IL-15Rα) was included because IL-15 signaling is essential for NK cell development, homeostasis, and effector function (doi: 10.3389/fimmu.2021.610789), making it a biologically relevant marker to assess in this setting. These justifications have been incorporated into the manuscript text to ensure the rationale is transparent to the reader.

Reviewer #2 (Remarks to the Author):

Thank you for the substantial revisions and for adding new data to address the reviewers' concerns. Few remaining points should be addressed before the manuscript is finalized.

The manuscript appropriately states that the exact mechanism linking NKG2AhighNKp30⁻NKp46⁻ cells to post-treatment control remains to be established. This point should be highlighted more explicitly as a study limitation in the Discussion, especially because the current data remain largely associative and do not directly demonstrate an NKG2A/MHC-E dependent mechanism.

We thank the reviewer for this important remark. We have now added an explicit statement in the Discussion (lines 595–597) emphasizing that our findings are associative in nature and that future mechanistic studies will be required to directly demonstrate an NKG2A/MHC-E-dependent mechanism underlying the observed association with post-treatment control.

The authors showed in the "response to reviewer" that the subset frequencies were recalculated relative to total live cells or lymphocytes and that the same trends were observed. However, those data were not included in the supplementary figure. They should be added.

We apologize for this omission. As suggested, we have now included the subset frequencies calculated relative to total live cells as a new Figure S5 in the revised manuscript. This addition ensures full transparency regarding the robustness of our findings across different normalization strategies.

In Figure 6E, x-axis seems wrong. It should be NKG2Ahigh and NKG2Alow. Now it says CD4 and NKG2Alow.

We thank the reviewer for raising this point, which we are happy to clarify. The x-axis labeling is intentional: the left panel represents CCR5 expression on CD4+ T cells alone (CD4) or in presence of autologous NKG2A^{high} cells (+NKG2A^{high}), and the right panel corresponds to similar experiments with CD4+ T cells cultured in absence (CD4) or presence of autologous NKG2A^{low} cells (+NKG2A^{low}). Therefore, the axis reflects the two conditions of culture.

The resolution of the FMO/unstained control plots is currently too low. Please provide higher-resolution images. Please include an NKG2A FMO control as well.

We thank the reviewer for this comment. We have replaced the FMO and unstained control plots with higher-resolution versions in the updated Figure S2.

The axes in Fig. S2B and Fig. S3C are difficult to read compared with the rest of the manuscript. Please replace these panels with higher-resolution versions.

We apologize for this inconsistency in image quality. We have replaced both panels with higher-resolution versions to ensure readability and visual consistency with the rest of the manuscript.

Both supplemental tables are currently labeled Table S1.

We apologize for this error. We have corrected the labeling so that the two supplemental tables are now properly designated as Table S1 and Table S2, respectively.

In the Figure 6 legend, “NKp36” appears where it should be “NKp46.”Please correct this in the figure legend and check for typo elsewhere in the manuscript.

We sincerely thank the reviewer for spotting this typographical error. We have corrected "NKp36" to "NKp46" in the Figure 6 legend and have carefully reviewed the entire manuscript for similar typos, correcting all instances identified (Figure S12).
